# Recent Advances in Multifunctional Reticular Framework Nanoparticles: A Paradigm Shift in Materials Science Road to a Structured Future

**DOI:** 10.1007/s40820-023-01180-9

**Published:** 2023-09-22

**Authors:** Maryam Chafiq, Abdelkarim Chaouiki, Young Gun Ko

**Affiliations:** Materials Electrochemistry Group, School of Materials Science and Engineering, Yeungnam University, Gyeongsan, 38541 Republic of Korea

**Keywords:** Porous organic framework, Reticular chemistry, Reticular framework nanoparticle, Environmental remediation, Multifunctional material

## Abstract

Porous organic frameworks (POFs) have become a highly sought-after research domain that offers a promising avenue for developing cutting-edge nanostructured materials, both in their pristine state and when subjected to various chemical and structural modifications. Metal–organic frameworks, covalent organic frameworks, and hydrogen-bonded organic frameworks are examples of these emerging materials that have gained significant attention due to their unique properties, such as high crystallinity, intrinsic porosity, unique structural regularity, diverse functionality, design flexibility, and outstanding stability. This review provides an overview of the state-of-the-art research on base-stable POFs, emphasizing the distinct pros and cons of reticular framework nanoparticles compared to other types of nanocluster materials. Thereafter, the review highlights the unique opportunity to produce multifunctional tailoring nanoparticles to meet specific application requirements. It is recommended that this potential for creating customized nanoparticles should be the driving force behind future synthesis efforts to tap the full potential of this multifaceted material category.

## Highlights

- This review summarizes the quarter-century of reticular chemistry.
- Preparation strategies and characterization of reticular framework nanoparticles (RF-NPs) are systematically reviewed.
- Biomedicine, gas valorization, energy storage and other newer applications of RF-NPs are involved
- Future potential and challenges of RF-NPs are prospected.

## Graphical abstract

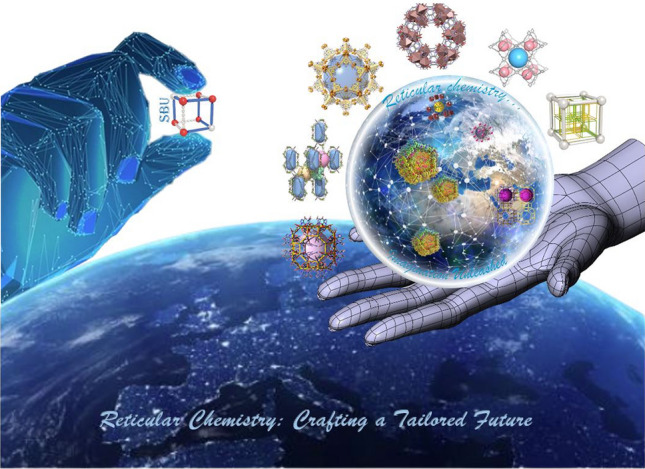

## Introduction

At its core, chemistry, first and foremost, is a fascinating science that delves into the captivating world of atoms and molecules on their spatial arrangement. As a material science community, assuming we find ourselves engaged in the intricate art of concocting chemical interactions or dedicatedly scrutinizing the nuanced characteristics of various materials, there exists an inherent allure that compels us to delve into the depths of understanding and ultimately conquer the intricate topological features that define molecular frameworks [1, 2]. In fact, being able to manipulate these structures is the key to unlocking a whole new realm of possibilities in the purview of chemical materials science. However, the discovery of solid-state structures has often been a matter of serendipity rather than predictable planning. While this approach has resulted in significant breakthroughs and will persist in pursuit of the goal, there is an imminent requirement for regulating materials at the atomic scale to create customized “on-demand” materials [3–8].

Indeed, chemists have always been fascinated by how atoms combine to form molecules and how they interact. Despite their focus on practical applications, they can't ignore these fundamental questions which underlie all their observations and discoveries. Even while developing materials for societal benefit, chemists find themselves returning to atomic and molecular structure, trying to control nature's puzzles. In this vein, the exploration of reticular chemistry is a promising approach toward creating unique architectural sequences of periodic spatial arrangement backbone materials that serves as a bridge between our ability to comprehend and manipulate molecules in dimensions 0D and 1D, and the potential for manipulating molecules in higher dimensions, such as 2D and 3D keeping in mind the noble aim of “bending them to our will” [9].

**Fig. 1:**
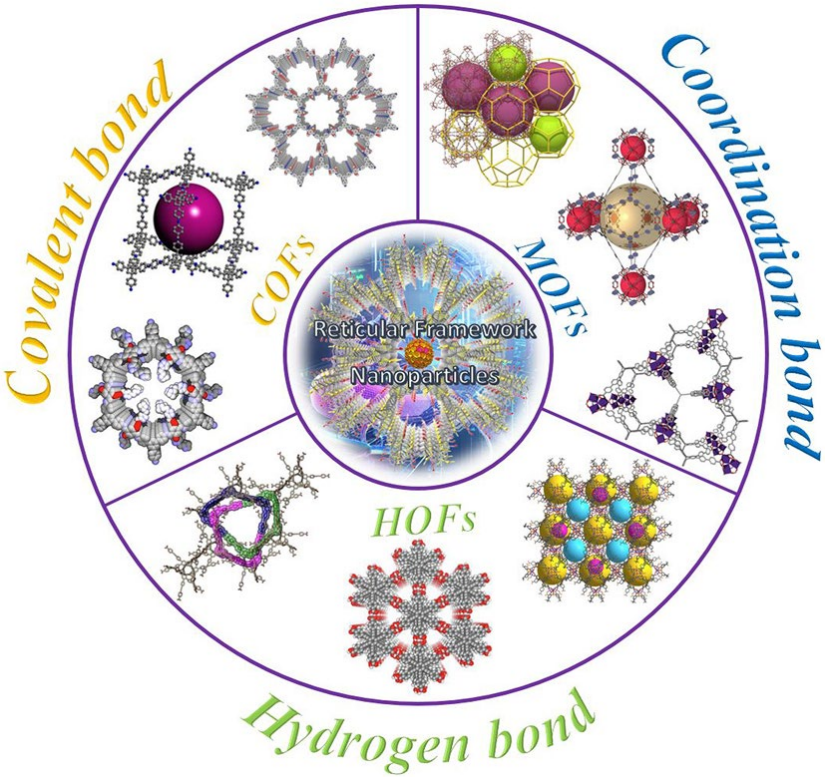
Classification diagram of POF-derived nanomaterials

**Fig. 2:**
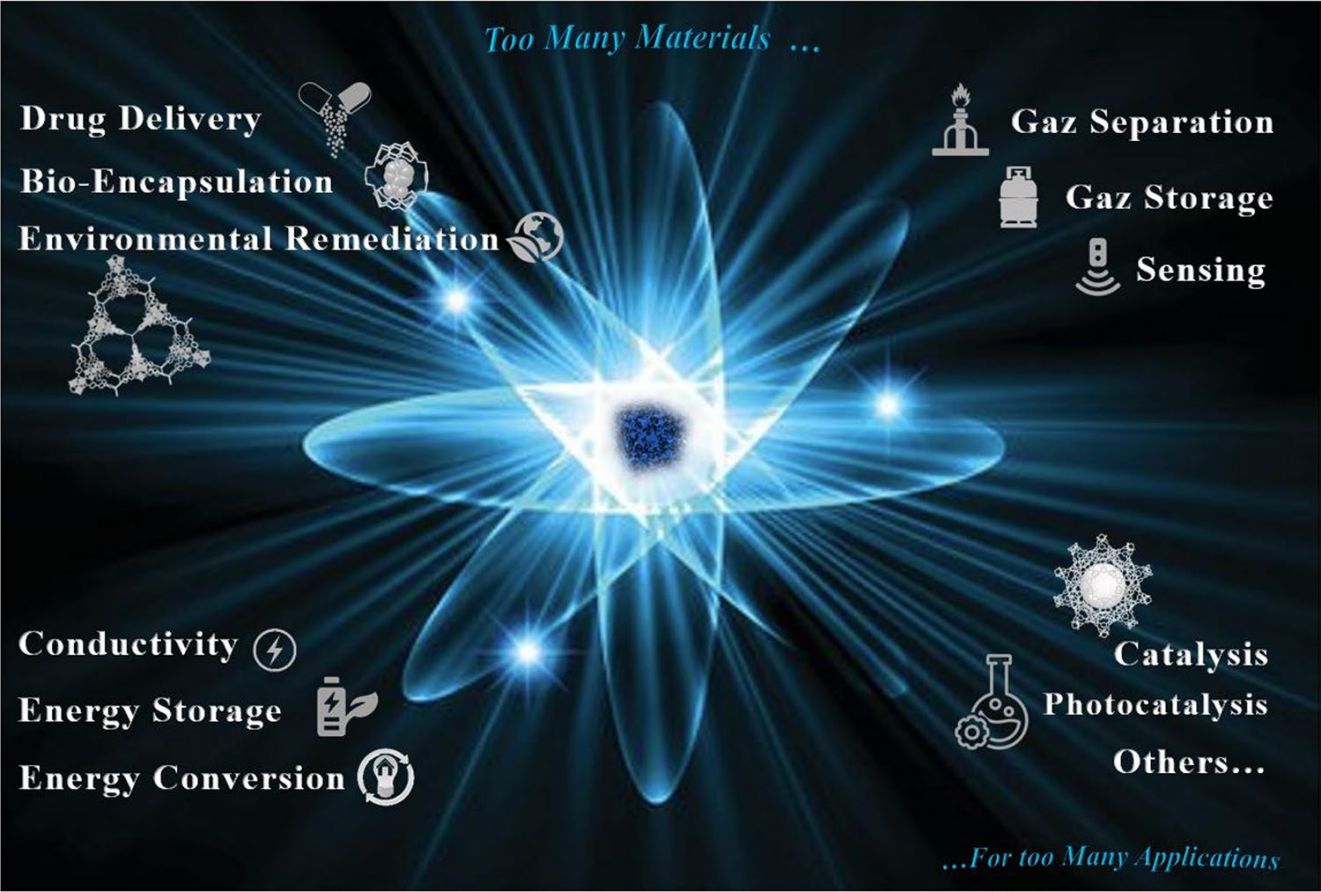
Overview of the diverse applications of RF-NPs

**Fig. 3:**
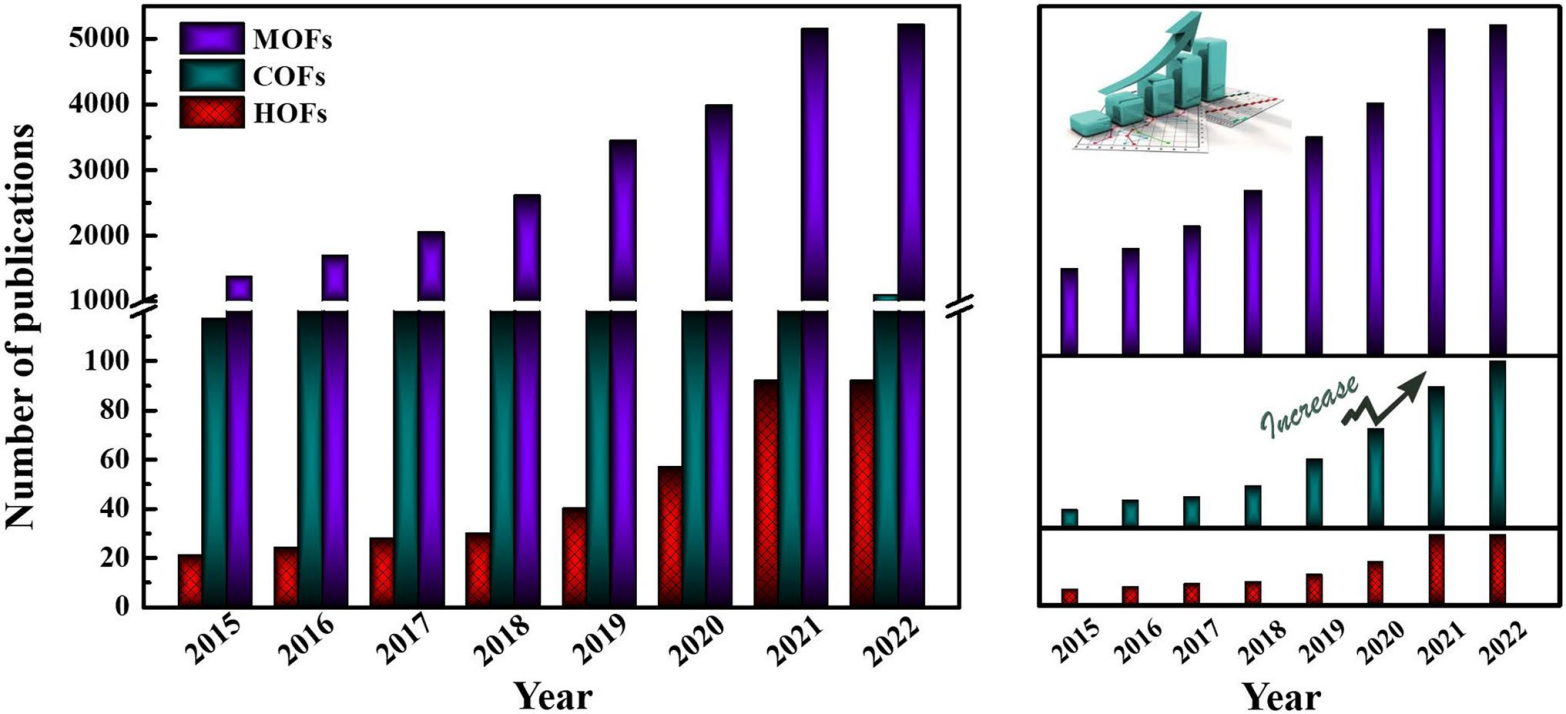
Body of scientific research papers exploring scholarly studies on RF-NPs in the last 8 years, as of April 2023. The data is based on a Web of Science keyword search using the terms “MOFs” (violet), “COFs” (green), and “HOFs” (red). (Color figure online)

Reticular chemistry, derived from the Latin term “reticulum” meaning “net-like” involves the connection of individual building units such as molecules and clusters using robust bonding to form extensive and coherent architectures with highly ordered arrangement in a designed manner [10–12]. By combining the best of inorganic chemistry, organic chemistry, and materials science and engineering, reticular chemistry is producing a treasure trove of novel materials with remarkable properties. Considering the number of unique structures catalogued so far in the Cambridge Structural Database surpassing an impressive milestone [13], this field is at the forefront of scientific discovery and innovation [14–18]. Indeed, within the field of reticular chemistry, 2D and 3D porous organic frameworks (POFs) are among the most significant categories of reticular materials (Fig. 1). These structured materials are characterized by extended crystalline solids which are formed by linking secondary building units (SBUs) joining metal-containing sequences through powerful directional bonds [19–21]. The exceptional capacity of reticular chemistry to create bespoke materials based on POFs structures positions this area as a leading contender for addressing a wide range of issues linked to energy [22–25], separation [26, 27], water capture [28, 29], catalysis [30, 31], gas storage [32, 33], sensing [34, 35], diagnosis [36, 37], and therapy [38, 39] (Fig. 2). This outstanding success makes the field a subject of extensive research and development, with an abundance of scholarly articles dedicated to POFs synthesis, characterization, and prospective practical uses. In this context, the scientific community is actively engaged in studying various aspects of POFs, leading to a substantial and continually thriving literature base on this topic (Fig. 3).

**Fig. 4:**
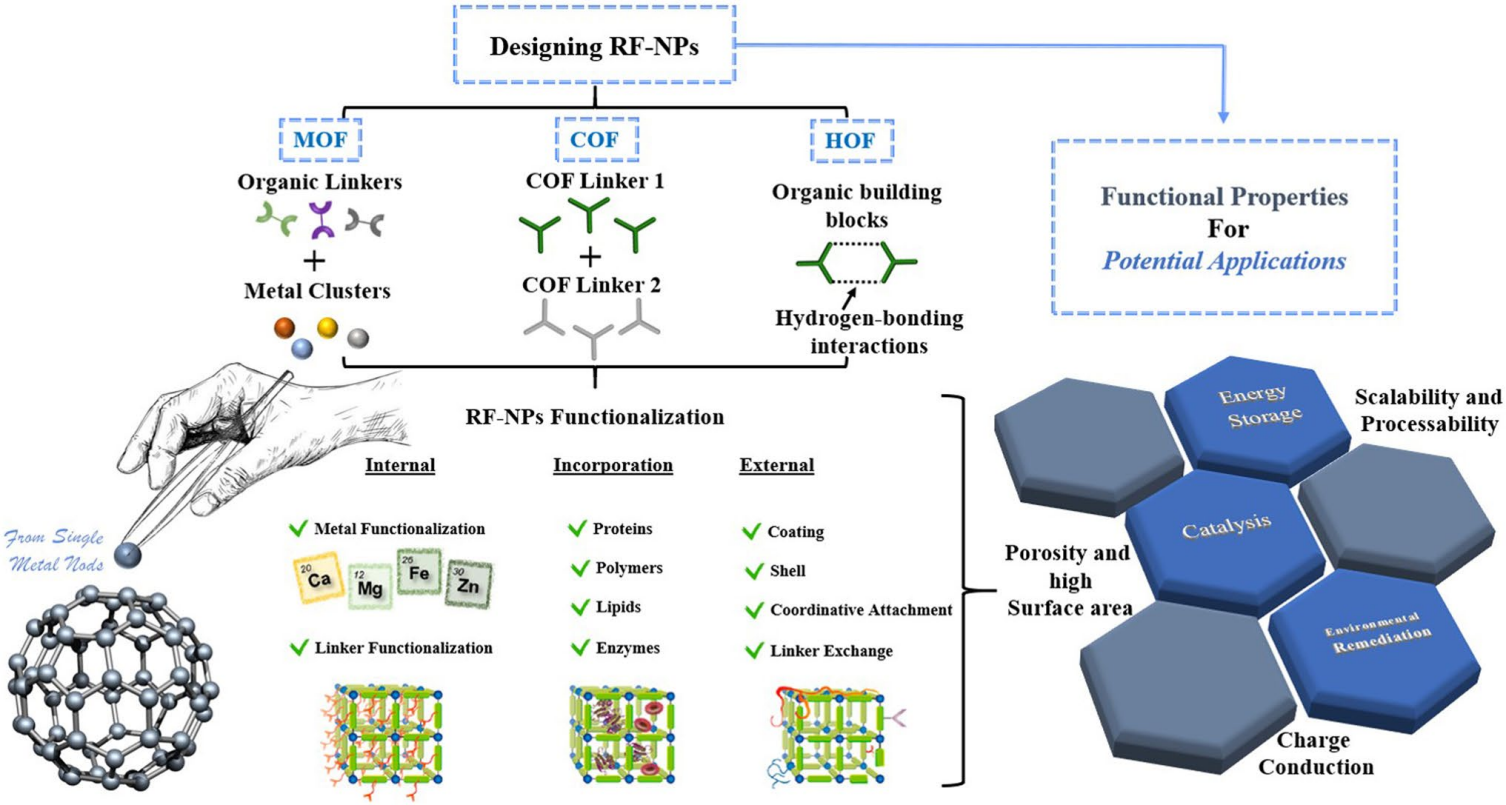
Overview of functionalization pathways for RF-NPs. Internal and external surface modification as well as guest incorporation for various intrinsic and extrinsic properties enabling diverse applications

The evolution of porous materials with diverse topologies has a rich history, traced back to the assembly of inorganic clusters into extended frameworks [40]. This was followed by the synthesis of metal–organic frameworks (MOFs), which involved linking organic molecules and metal ions. Subsequently, covalent organic frameworks (COFs) emerged, which linked organic molecules together, and hydrogen-bonded organic frameworks (HOFs) represent the latest generation of such materials (Fig. 4). MOFs are an intriguing class of inorganic–organic hybrid porous materials constructed from metal containing nodes (metal ions or clusters) and organic linkers. MOFs are also known as porous coordination polymers (PCPs) or porous coordination networks (PCNs) due to their interconnected coordination bonds and polymeric nature [41–43]. COFs are a type of porous materials characterized by their non-metallic composition. These materials are formed by arranging pre-designed organic-molecular building units into a well-ordered and repetitive structure through reversible covalent bonds. These frameworks have also garnered significant interest as promising platforms for creating a class of amazing organic materials in a 2D or 3D fashion [44–46]. Compared with MOFs and COFs, HOFs have emerged as a novel category of crystalline porous materials. They are constructed by assembling organic units, comprising both pure organic and metal-containing organic moieties, through robust hydrogen bonding interactions. The stability of these frameworks can be further reinforced through framework interpenetration and various weak intermolecular interactions, including π-π interactions, van der Waals interactions, etc. [47, 48]. MOFs are typically synthesized using a one-pot self-assembly approach, where the metal-containing nodes are formed in situ. Throughout the reaction, the ligand remains a consistent component, although in some cases, the ligand itself is generated during the solvothermal procedure. The solvothermal or hydrothermal method is a process that involves conducting a chemical reaction under specific conditions of high temperatures and pressures, utilizing either aqueous or organic solvents as well as reactants to facilitate the reaction in a heterogeneous system. The resulting MOF's topology is determined by the geometry of the ligand. Functional groups are commonly attached to the linker through pre- or post-synthetic organic synthesis. To control the reaction rate and facilitate the growth of single crystals, the slow evaporation of solvents at room temperature is often employed [49].

The development of COFs is driven by the principles of reticular chemistry, which involves careful consideration of the topology and connection modules of the building blocks. These building blocks are connected through reversible covalent bonds to create crystalline and highly porous structures. COFs employ step-growth polymerization to propagate chains in a 2D or 3D manner. This approach ensures that the rigid backbones of COFs are strategically guided, allowing for the formation of highly ordered and structured frameworks. The formation of COFs entails the incorporation of both covalent bonds and noncovalent interactions, which play a crucial role in shaping the extended and well-defined crystalline structures. What sets COFs apart is that their framework structure is solely determined by the choice of monomers. This unique characteristic allows COFs to be fully pre-designed and synthetically controlled, offering a level of structural predictability that is rarely achievable with traditional polymers and other molecular frameworks. The design of 2D COFs is relatively straightforward. These structures typically exhibit a π–π layer-stacking arrangement along the *z*-axis direction. Therefore, the key factors to consider are the length and angle of the building units, along with their connections to form a flat structure on the *X*–*Y* plane. Meanwhile, crystallization challenges are more significant in 3D COFs compared to 2D structures [50]. Synthesis conditions for 3D frameworks are more rigid and precise, especially for those monomers with functional moieties. The increase in dimensions poses significant challenges in the synthesis and structural determination of 3D COFs. In addition to considering the length, angle, and connection of the building units in three dimensions, achieving repetitive interconnected 3D space presents notable difficulties. Therefore, the widely strategy for constructing 3D COFs mainly involves the combination of tetrahedral-type (Td) building units (generally composed of a tetrahedral core and four identical functional groups) with suitable building blocks.

HOFs are formed through hydrogen bonding, which is inherently weaker and more reversible compared to coordination or covalent bonds. The distinctive characteristics of hydrogen bonding connections result in intriguing differences between HOFs and MOFs or COFs. Notably, HOFs exhibit advantages such as solution processability, facile synthesis and characterization, and the ability to undergo easy healing and purification through simple recrystallization. The design of molecular building blocks plays a crucial role in determining the porosity, topology, and stability of HOFs, thereby serving as the fundamental principle for their construction. In this regard, a great number of building blocks can be utilized for HOFs construction which take advantage of the fundamental motifs, including carboxylic acid, pyrazole, pyridine, 2,4-diaminotriazine, benzimidazolone, imide, imidazole, amide, boronic acid, etc. These building blocks enable the formation of H-bonding interactions within the HOF structures. Chen et al. [47] summarized several approaches for designing and synthesizing HOFs that exhibit high stability and framework rigidity: (i) Enhance intermolecular interactions during molecular assembly to strengthen HOF formation. (ii) Utilize rigid organic ligands with a stereoscopic backbone to construct robust HOF structures. (iii) Introduce appropriate interpenetrations within HOFs. (iv) Incorporate additional intermolecular interactions such asπ-π stacking and vdW forces to promote stable HOF construction. (v) Prevent the formation of any terminal hydrogen-bonding donor and acceptor moieties. These strategies aim to optimize the stability and rigidity of HOFs for various applications.

Herein, and after a quarter-century of dedicated research, we will summarize the critical accomplishments made by experts within the scope of reticular chemistry. From the creation of innovative materials with unprecedented properties to the design of sophisticated techniques for their synthesis and characterization, the advances made in this area and their impact on materials science engineering are highlighted in this contribution. In this cutting-edge review, we explore the fascinating world of reticular materials and their anticipated uses have far-reaching implications across a multitude of disciplines and industries. In this Outlook, we anticipate that it is not only a broad overview of the latest technologies related to these materials, but also outlines the inherent challenges and opportunities associated with their use. By delving into these exciting developments, we hope to inspire future research in this young dynamic field paving the way for new multivariate reticular structures.

*Reticular chemistry’s potential for functionalizing structures, combined with its remarkable precision in synthesis and modification, leads to an infinitude of artistry possibilities of material design in a controlled fashion.*

## Quarter-Century of Reticular Chemistry

### A Journey through the World of Coordination Chemistry: From Single-Metal Nodes to SBUs

**Fig. 5:**
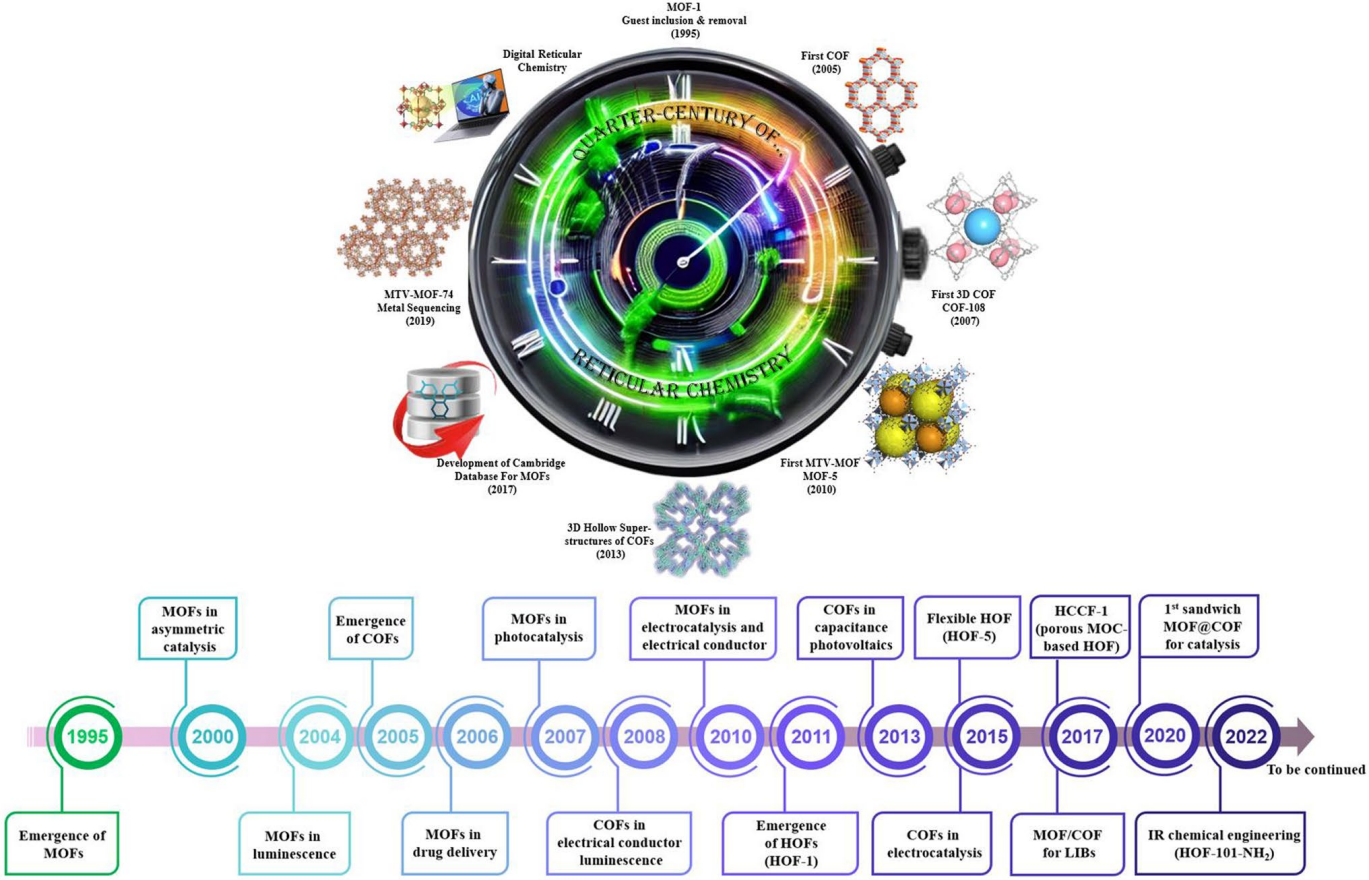
From early breakthroughs to recent advancements. Illustrative chronology highlighting key milestones in the field of reticular frameworks over time

The expedition starts in 1995 with the synthesis of the first reticular framework (Fig. 5), when it was discovered that metal ions could be bound to charged bonding agents, such as carboxylates, leading to the formation of crystalline 2D structures. Then by ingeniously interlacing inorganic clustered SBUs with shape-defining connectors through formidable molecular bonds, a significant breakthrough was made [51, 52]. During the initial stages of their development, the primary objective was centered on synthesizing new MOF structures. However, as the field progressed, there was a gradual transition toward studying the performance and applications of MOFs, which gained momentum with the emergence of stable MOFs like UiO, MIL, and ZIF. In 1999 [53], a notable advancement was achieved in synthesizing MOF-5, a highly porous and robust MOF. MOF-5 is constructed by connecting octahedral Zn_4_O(CO_2_)_6_ clusters with ditopic 1,4-benzenedicarboxylate units to form a cubic framework. The inherent structural strength of MOF-5 enables convenient gas sorption measurements, greatly aiding the advancement of this burgeoning research field in the chemistry and materials science communities. Yaghi et al. [54] utilized MOF-5 as a starting point and employed modifications in the length and functional groups of aromatic carboxylic acid ligands to successfully synthesize a range of MOFs (referred to as IRMOF-n, where n ranges from 1 to 16 and IR denotes isoreticular) with the same topological structure as MOF-5, resulting in the creation of ultra-large-pore MOFs. Furthermore, the synthesis of MOF-74 (Zn_2_(dhbdc), where dhbdc represents 2,5-dihydroxy-1,4-benzenedicarboxylate) was first reported in 2005 [55]. Subsequently, extensive research has been conducted on utilizing the dhbdc linker with various metals, leading to notable advancements in this area. Chen's group reported the successful synthesis of a coordination polymer known as [Zn(bim)_2_]∙(H_2_O)_1.67_ (also referred to as MAF-3 or ZIF-7), which incorporated zinc(II) ions and the bridging benzimidazole anion [56]. This structure exhibited a hexagonal space group and displayed a sodalite (SOD) topology. Subsequently, the research group described three different MOFs with zeolitic topologies through the implementation of substituent modulation and a mixed-ligand strategy. Nevertheless, it was Yaghi's group that bestowed these materials with a distinctive naming convention, highlighting their contribution to the field. In 2006, the same group proposed the systematic name of “zeolitic imidazolate frameworks” and introduced the detailed structure of ZIF-1 ~ ZIF-12 [57]. ZIF-20 ~ ZIF-23, ZIF-68, ZIF-69, ZIF-70, ZIF-ZIF-95, and ZIF-100 were reported in subsequent studies in 2007 and 2008, respectively [58, 59].

On the other hand, COFs have been undergoing a progressive development trajectory since their initial report in 2005 [44], following a path similar to that of MOFs. As time has passed, they have steadily moved from the synthesis stage to practical applications, marking a significant achievement in their overall advancement. COF-1 and COF-5, both featuring the hcb topology, were synthesized through the self-condensation of p-phenylenedicarboxylic acid or the co-condensation with hexahydroxytrimethylene. In 2007, Yaghi and coworkers [60] made notable progress by introducing two new COF materials. They successfully synthesized COF-105, featuring the ctn topology, and COF-108, with the bor topology. These advancements marked a significant milestone in the field as they demonstrated the evolution of COF materials from 2 to 3D structures. Notably, the connecting bonds in COF-105 consisted of B_3_O_3_ rings, while COF-108 featured C_2_O_2_B rings, showcasing the diversity and versatility of COF structures. Following their previous achievements, in 2009, the research group achieved another important milestone by creating the first imine-based COF, named COF-300. This COF showcased a unique fivefold interpenetrated dia topology, which was obtained through the reaction between tetra-(4-aminophenyl)methane and terephthaldehyde [61]. In 2020, Yaghi et al. [62] made an ingenious breakthrough by developing cubane-like linkers using 1,4-boronophenylphosphonic acid. These linkers exhibited a valency of 8 and could undergo condensation reactions to form COFs. This innovative approach significantly broadened the range of connectivity and topological variations achievable in COFs, opening up new avenues for exploring their structural diversity. In 2021, a notable breakthrough was accomplished by Wei et al. [63] as they achieved the synthesis of 2D COF single crystals in a remarkably short time frame of 2 to 5 min using sc-CO_2_. The resulting single crystals exhibited a remarkable size of up to 0.2 mm. This novel synthetic protocol marks a significant improvement in the efficiency of COF single crystal synthesis, offering promising prospects for scalability and practical applications of COF materials. Finally, a remarkable advancement in COF synthesis was achieved in 2021, marking the most recent milestone in the field. This significant achievement showcases the continuous progress in the development of COFs, demonstrating their potential in producing nanoscale structures with unique properties and expanding the exploration of COF architectures with higher valencies.

HOFs have also been proposed as potential porous materials more than two decades ago. Despite early attempts to create HOFs with included guests in the early 1990s, it wasn't until 2011 that HOFs with confirmed permanent porosity, validated through gas sorption isotherms, were first documented. Chen et al. [64], building upon the groundbreaking research by Wuest et al. [65, 66], made a significant contribution in 2011 by reporting the first example of a microporous HOF with permanent porosity and exceptional selectivity for C_2_H_2_/C_2_H_4_ separation, referred to as HOF-1. Thereafter, the establishment of permanent porosity for HOFs marked a transformative milestone in the progression of this area of research. In subsequent studies, the same research group utilized different DAT-functionalized building blocks to create a series of HOFs featuring open pores. This set of HOFs, namely HOF-2 [67], HOF-3 [68], HOF-5 [69], HOF-6 [70], HOF-9 [71], and HOF-10 [72], demonstrated successful construction and pore accessibility. When considering the labile nature of hydrogen bonds, which are considerably weaker than covalent and coordinative bonds, it is well understood that the activation of HOFs poses a more formidable task compared to other porous frameworks (zeolites, MOFs, COFs). Thus far, HOFs have demonstrated immense promise as a versatile platform for investigating new porous materials in a wide range of applications, including gas storage and separation, conductive and optical applications, heterogeneous catalysis, and biomedicine.

### Art of Design: Leveraging the Power of Ligand Diversity and Derived Nets for RF-NPs Construction

**Fig. 6:**
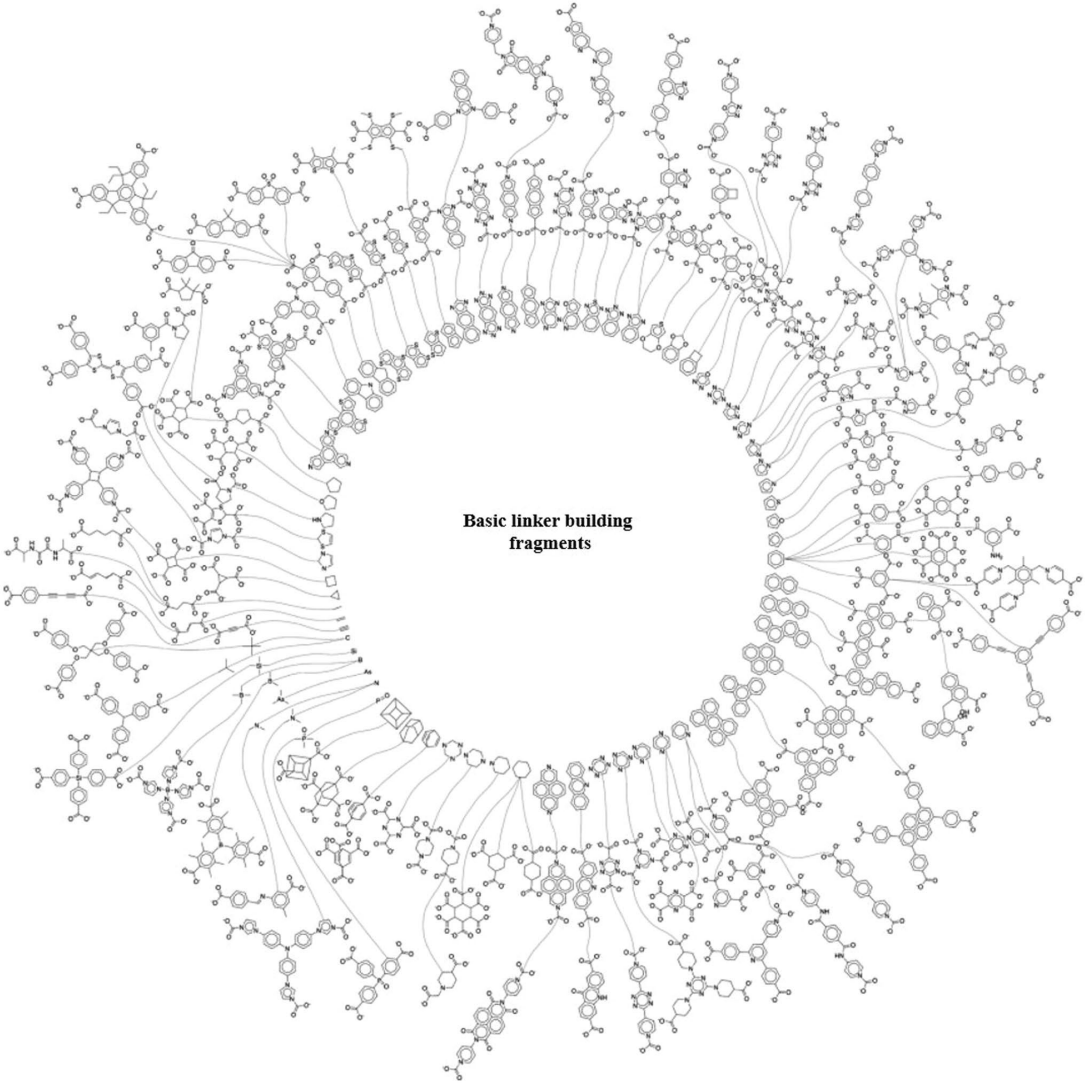
Power of ligand diversity. An analysis was carried out on the linkers present in all reticular framework structures from the CoRE database. The results of this analysis have been represented in a scaffold tree plot, which highlights the basic building fragments of the latest RF-NPs that have a high occurrence rate. These building fragments are depicted in the inner circle of the plot. The second and third circles of the plot illustrate the linkers that have been derived from these building fragments. The plot provides a comprehensive visual representation of the linkers and building fragments present in state-of-the-art RF-NPs, which can be useful for further research in this field. Reproduced with permission from Ref. [74]. Copyright 2021, Springer Nature

**Fig. 7:**
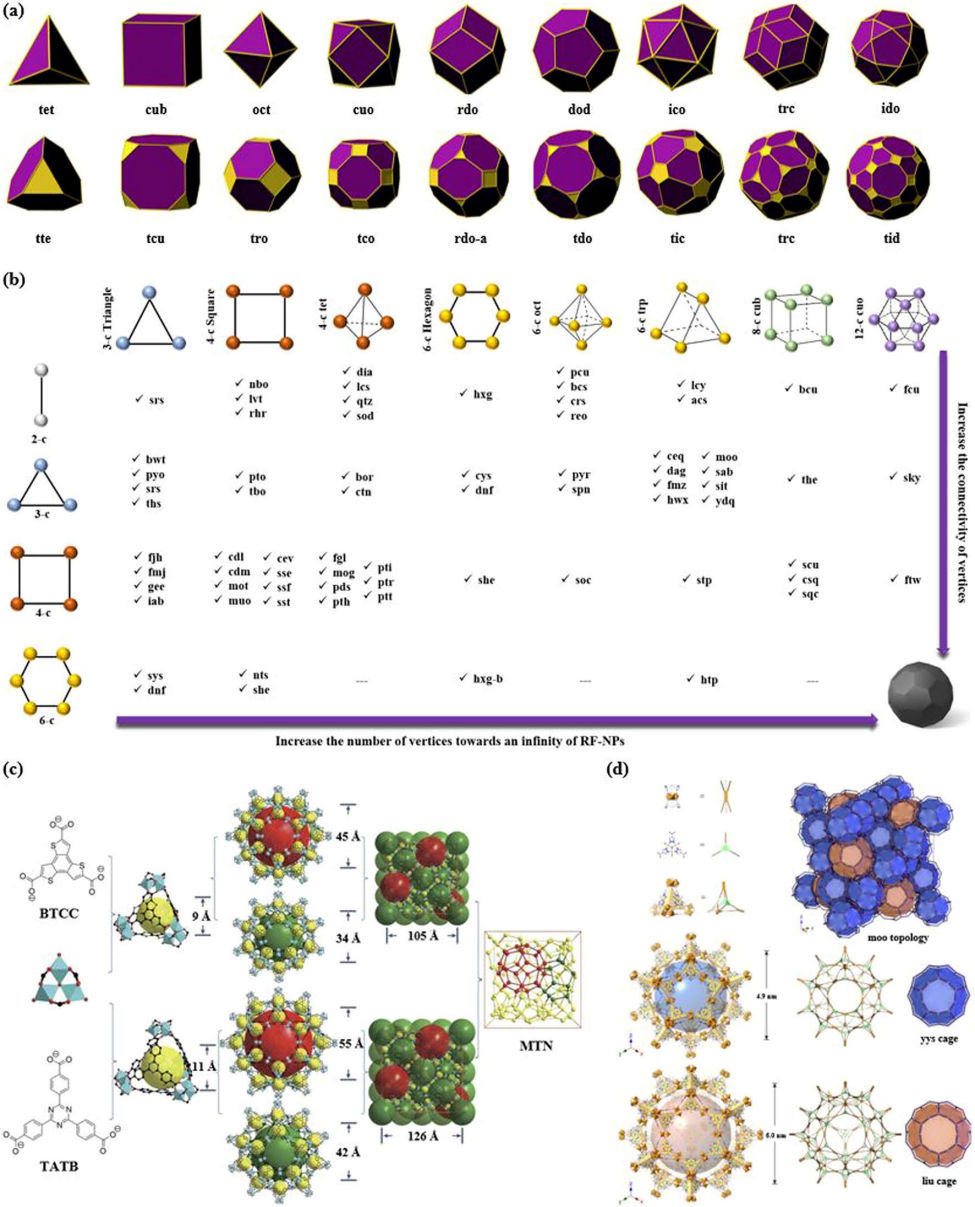
Overview of the opportunities in derived nets for RF-NPs construction. **a** Illustrative comparison of nine edge-transitive polyhedra (top) and their corresponding augmented (truncated) equivalents (bottom) (reproduced with permission from ref. [80]. Copyright 2014, Royal Society of Chemistry). **b** Synergistic interplay between various design concepts of nets used to represent binary frameworks, as produced by the principles of reticular chemistry. The results obtained do not modify the discernible pattern that indicates that larger polyhedra can be produced by utilizing fewer vertex connections, particularly when the number of vertices is comparable. **c**, **d** The meticulous design and successful construction of two highly porous materials, c HP PCN-332/333, composed of BTTC/TATB linkers and 6-connected Al clusters, (reproduced with permission from Ref. [78]. Copyright 2015, Springer Nature), and d HP MOF-919-Sc, constructed from building blocks with reduced connectivity (reproduced with permission from Ref. [79]. Copyright 2014, American Chemical Society)

Herein, an emphasis is on the notion of “Reticular polymorphism” (depicted in Figs. 6 and 7), a term we have coined to convey the range of variations in structures and composition of framework assemblies toward an infinity of RF-NPs. Reticular polymorphism is a core concept in the field of POFs chemistry that has expanded the horizons of synthesizing frameworks with a wide range of geometries, compositions, complexities, metrics, and degrees of order [73]. Through the amalgamation of these diverse facets, a singular, exceptionally advanced substance emerges, representing a paradigm shift in design with unique properties and functions.

It is a remarkable fact that there are only nine polyhedra which possess the property of being edge-transitive. The particular aspect is of great significance in the field of solid-state engineering, as the modified or altered version of these polyhedra can serve as an ideal blueprint for the construction of associated designs, which can then be assembled into related reticular frameworks. Figure 7a displays the nine polygons with the property of edge-transitivity, in conjunction with their corresponding truncated polyhedra. This information is critical for designing and synthesizing new materials with specific properties, particularly in the area of porous materials for encapsulation [75–77]. Upon closer examination of these fundamental structures, it becomes evident that among the total of 9 architectures, 7 can be crafted using only two basic triangular and square structures, or a blend of both, leaving only a minority that require different node combinations. These nodes, namely tetrahedron, cube, octahedron, cuboctahedron, rhombic dodecahedron, rhombicuboctahedron, and icosidodecahedron, form the basis of the majority of these edge-transitive polyhedra. Interestingly, the three edge-transitive polyhedra (octahedron, cuboctahedron, and icosidodecahedron) that are based on 4-c nodes share a homogeneous square point arrangement, making them prime candidates for the implementation of reticular materials in crystal chemistry. It is worth noting that the arrangement of vertices within a specific polyhedron determines the spatial coordinates where the Molecular Building Blocks (MBBs) are connected for the creation of the corresponding reticular frameworks. Furthermore, there exist merely three distinct methods of connecting squares with a single type of edge to form a polyhedron, whichever further highlights the significance of these edge-transitive polyhedra in the construction of reticular materials. Figure 7b presents a visualization of the primary edge-transitive nets that result from combining ligands and SBU patterns commonly used in reticular chemistry. This representation supports the assertion that the vast structural polymorphism observed in reticular materials is largely attributable to the broad range of available SBU and cage geometries. In this setting, Feng et al. [78] developed a unique structure PCN-333, made up of M_3_(μ_3_-O)(OH)(H_2_O)_2_ units connected by organic linkers along the faces of supertetrahedra (Fig. 7c). By doing so, they were able to create two distinct mesoporous cages that could potentially be used as host matrices for enzyme encapsulation. The smaller dodecahedral cage is formed by connecting 20 supertetrahedra at their vertices to create a cavity with a pentagonal window that is 25.9 Å in diameter. In contrast, a sphere is formed by the arrangement of 24 supertetrahedra around the larger hexacaidecahedral cage, featuring windows of both pentagonal and hexagonal shapes measuring 30 Å in diameter. These cages have varying inner diameters: the supertetrahedral cage measures approximately 11 Å, the dodecahedral cage spans about 34 Å, and the hexacaidecahedral cage has a size of around 55 Å, as shown in Fig. 7c. In 2019, Deng's research team [79] has reported the successful synthesis and characterization of MOF-919, a metal–organic framework with a moo topology (Fig. 7d). The MOF is composed of two types of cages, yys and liu, with 50 and 70 vertices, respectively. It was constructed using Fe/Sc/Al-SBUs and Cu-SBUs, which form the 6-c and 3-c nodes, respectively, leading to the formation of distinct iso-reticular frameworks, each displaying unique attributes. Using powder X-ray diffraction and 2D-small angle X-ray scattering patterns the angles and sizes of the super tetrahedra, yys and liu cages are measured, providing a detailed characterization of the crystal structure of MOF-919 and offering insights into the construction of MOFs using a moo topology. Furthermore, it suggests that targeted structures can be achieved by selecting building units with appropriate shapes and sizes. Essentially, when building units with the same shape and connection are arranged in different ways, it can create frameworks that have the same structure but different properties. This is known as “isoreticular” chemistry” [54].

**Fig. 8:**
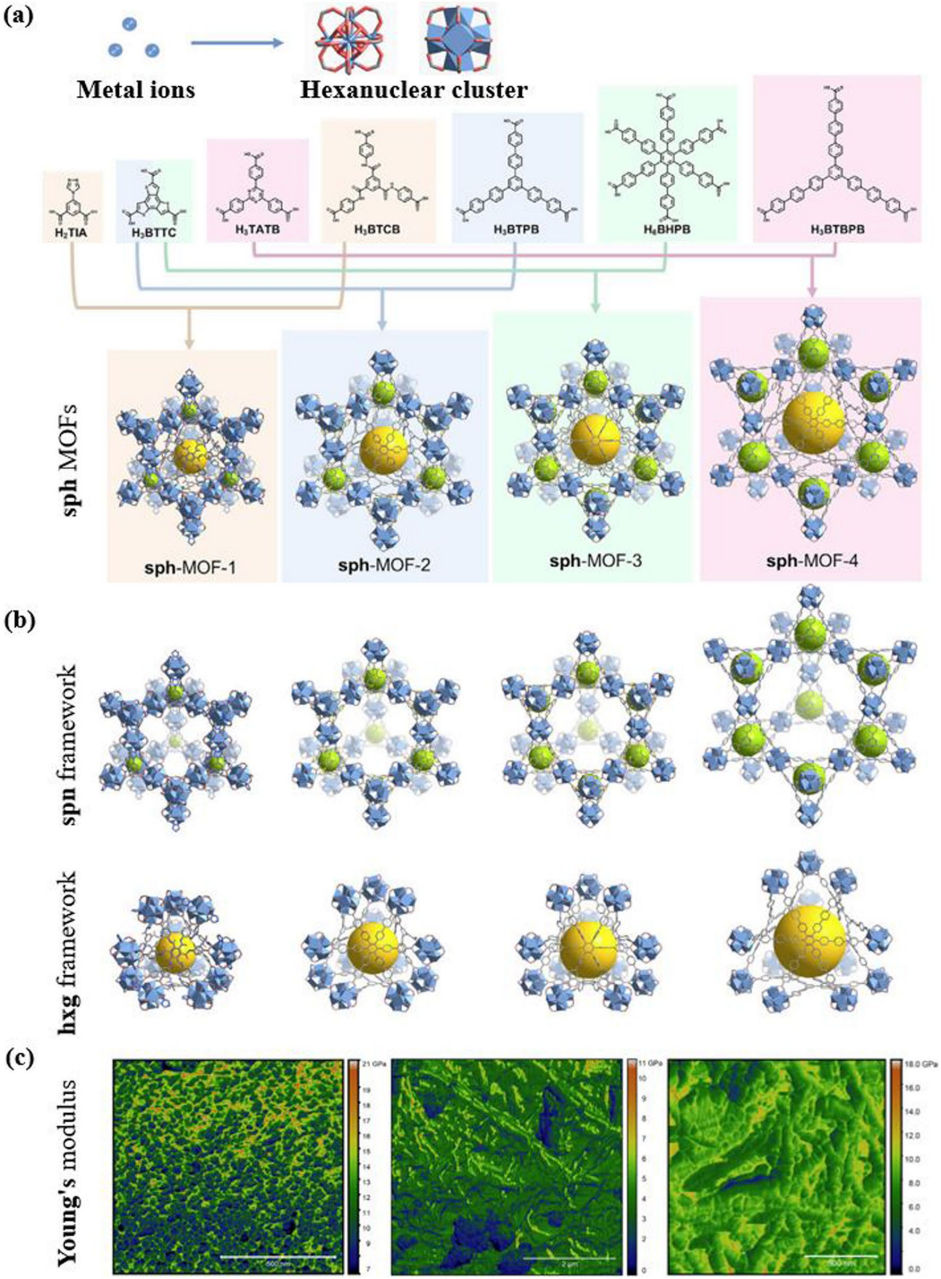
Illustration of the crystal structures and mechanical properties of sph-MOFs using various organic linker combinations. **a** Crystal structures of sph-MOFs-1 to -4 constructed with different organic linkers. **b** Depiction of the spn and hxg portions in the corresponding sph-MOFs. **c** Bimodal atomic force microscopy (AFM) characterization used to determine the mechanical properties of sph-MOFs (From left to right: Eu-sph-MOF-3, Eu-sph-MOF-4, and Eu-sph-MOF-5). Reproduced with permission from Ref. [81]. Copyright 2023, Elsevier Inc

In the current year-to-date, Jiang et al. [81] presented a novel approach to creating highly porous and mechanically stable metal–organic frameworks only by varying lengths of linkers (Fig. 8a, b). The approach involves merging two frameworks with a relatively delicate structure to form more exceptionally resilient particles. The study computationally evaluated the mechanical properties of sph-MOFs with different linker lengths, highlighting the importance of triangular rigidity for robustness in large-pore MOFs. Based on these findings, the researchers achieved a remarkable feat by designing and synthesizing a sph-MOF-5 based on rare-earth elements. This achievement involved the use of hexanuclear RE clusters, tritopic linkers, and large planar hexatopic linkers containing 19 phenyl rings. The mechanical properties of the combined-network MOFs were meticulously examined using amplitude-frequency modulation bimodal atomic force microscopy, revealing valuable insights into their structural robustness (Fig. 8c). The sph-MOF-4 has a Young's modulus ranging from 1 to 6 GPa, with an average of 3.7 GPa. Similarly, the sph-MOF-5 has a Young's modulus ranging from 3 to 12 GPa, with an average of 7.8 GPa (as shown in Fig. 8c). Interestingly, despite having similar pore environments and structure sizes, sph-MOF-5 exhibits higher mechanical stability than sph-MOF-4. This improvement is attributed to the hexatopic linker present in sph-MOF-5, which gives rise to triangles instead of parallelograms in the structure. These recent findings prove that even small changes in the molecular architecture of MOFs can significantly affect their mechanical properties. To sum up, isoreticular chemistry has emerged as a highly effective and adaptable technique in the field of materials science, with significant potential for advancing the creation and utilization of porous materials with precise and customized features. The versatility of this approach has already been demonstrated in various applications, and its influence is expected to expand as researchers continue to investigate novel strategies for designing and employing organized and porous materials with specific functionalities.

### Modulator, A “Key Pillar” for Complex System Design

**Fig. 9:**
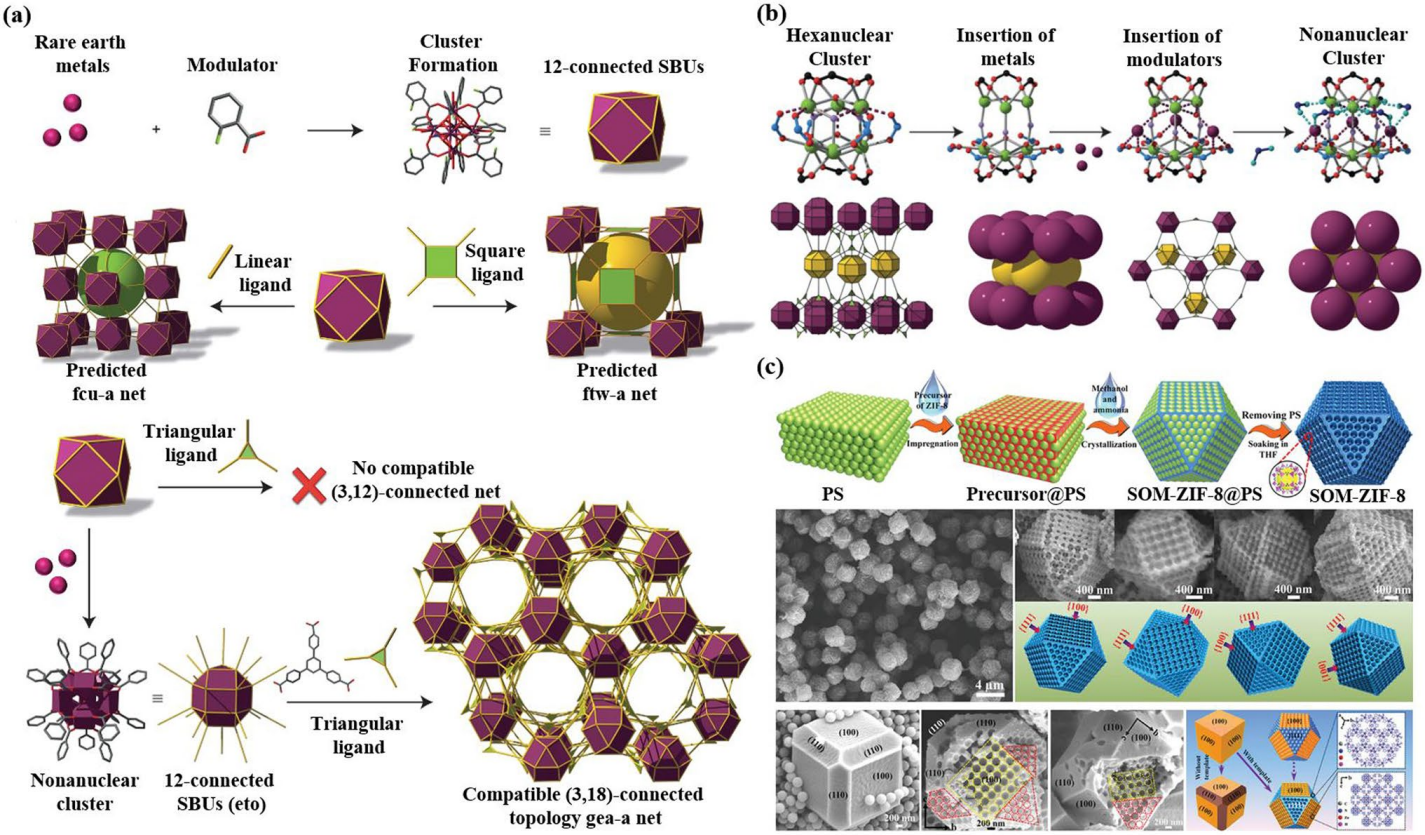
The role of modulators as a crucial cornerstone in the synthesis of complex systems. **a** Illustrative presentation describes how the incorporation of RE metal ions in combination with Benzoic acid, 2-fluoro as a modulator has been found to promote the formation of a hexanuclear RE cluster. This synergistic combination acts as a facilitator, influencing the self-assembly of the metal ions and ligands into a specific hexanuclear structure. The RE metal ions, known for their unique electronic properties, interact with the fluorobenzoic acid modulator to orchestrate the precise arrangement and coordination of the metal atoms in the cluster. This cooperative effect enables the formation of a stable hexanuclear RE cluster with distinct chemical and structural properties, which holds great potential for various applications in fields such as catalysis, magnetism, and luminescence. **b** A proposed route outlining the development from the hexanuclear to the nonanuclear cluster with their illustrations (bottom) showing the hexagonal close packing of the MBBs in gea-MOF-1. The packing is represented by two layers which are respectively colored in purple and yellow. (reproduced with permission from Ref. [82]. Copyright 2014, Springer Nature). **c** Polystyrene templated technique to create ordered macro-microporous MOF single crystals, which includes the use of a modulator (Adapted with permission from Ref. [90]. Copyright 2018, American Association for the Advancement of Science)

Modulated synthesis has become a common technique in the crystallization process of high-performance RF-NPs. This method involves the use of modulators that can affect the balance between formation and disintegration of structures, leading to controlled reorganization of structural elements in the synthesis process. Eddaoudi’s research team reported the successful synthesis and characterization of a rare-earth-MOF using a RE cluster as 18-connected inorganic metal building block (Fig. 9a) [82]. The discovery of this previously unknown cluster, which could only be accessed via the controlled strategy employing fluorinated benzoic acid, resulted in the identification of a non-self-dual (3,18)-connected gea net. Upon analysis of the [Y_9_(μ_3_-OH)_8_(μ_2_-OH)_3_(O_2_C–)_18_] cluster, it was found to have a strong similarity to the [Y_6_(μ_3_-OH)_8_(O_2_C–)_12_] cluster, but with the incorporation of three different metal ions and with the assistance of moderator molecules during the insertion process (Fig. 9b). The gea-MOF-1, which was isolated and characterized, exhibited potential for storing CH_4_. Notably, in the context of catalyzing the coupling of CO_2_ and epoxides, the gea-MOF-1 compound, which is yttrium-based, demonstrated remarkable catalytic prowess. The distinctive composition of this reticular framework enabled its remarkable catalytic performance, showcasing its potential as a catalyst for this coupling reaction. This finding represents a unique discovery, diverging entirely from any existing knowledge, and underscores the significant impact of the nanoparticle as an efficient catalyst in this specific chemical transformation. In the same orientation, Shen et al. were able to create an impeccably structured ZIF-8 material in the form of a single crystal, characterized by the presence of meticulously arranged and aligned macropores. The unique crystalline structure of this ZIF-8 material allows for precise control over the arrangement and distribution of its macropores, opening intriguing possibilities for diverse applications. To achieve this, they used a polystyrene nanosphere monolith as a template and combined it with ZIF-8 precursors (Fig. 9c). The resulting mixture underwent a crystallization process in methanol + NH_3_ solution. Upon the successful formation of the single-crystalline ZIF-8, a deliberate process was employed to remove the template of nanospheres. This meticulous procedure resulted in the creation of a hierarchical structure characterized by a well-defined and regular arrangement. To demonstrate the broad applicability of this method, the researchers successfully prepared several other types of POFs, such as DUT-5, MOF-808, MIL-53, UiO-67, and numerous others. The ability to generate such a diverse range of materials exemplifies the broad scope and adaptability of the technique. Each of these reticular materials possesses distinct structural and functional characteristics, enabling their utilization across a wide range of scientific and technological applications. By encompassing these notable examples, it becomes evident that the technique offers a robust and versatile approach for creating a vast repertoire of reticular materials with diverse properties and potential uses [83–89].

*Reticular chemistry, since its inception, has been a captivating realm that defies conventional boundaries. It thrives on the collaborative prowess of brilliant minds hailing from diverse scientific backgrounds, blurring the lines between organic and inorganic chemistry, materials science, and engineering. The progress made in reticular chemistry owes much to the contributions of scientists with diverse backgrounds, who work together and bring unique perspectives to the table. Today, the involvement of experts from different fields remains critical to the advancement of this area of research.*

### Polycatenated Reticular Frameworks: Interlocking Structures for Multifunctional Materials

Polycatenated reticular frameworks represent a fascinating class of multifunctional hybrid materials with diverse applications. These frameworks are constructed through the intricate coordination of multidentate organic ligands, leading to the formation of extended structures. While threaded molecules like catenanes and rotaxanes have primarily been limited to two-dimensional interlocking rings or polygons, the catenation of polyhedral cages has been relatively scarce. However, there have been suggestions that an infinitely extended architecture could be achieved through the polycatenation of discrete cages utilizing such threading principles [91]. Polycatenated RFs present a promising avenue for the design and fabrication of novel materials with unique properties. The concept revolves around linking together multiple discrete cage units through interlocked molecular threads. This approach allows for the creation of extended structures with interconnected porosity, providing opportunities for diverse applications.

The synthesis of polycatenated RFs is a complex endeavor that requires careful design and coordination of the ligands. The ligands need to possess specific geometric features and coordination preferences to ensure successful threading and subsequent interlocking of the cages. By choosing appropriate building blocks and optimizing reaction conditions, researchers have managed to achieve some remarkable examples of polycatenated frameworks. The exploration of polycatenated RFs is still in its early stages, and researchers are actively investigating new strategies for their synthesis and exploring their potential applications. By expanding the repertoire of ligands, refining synthetic methodologies, and studying the fundamental properties of these frameworks, scientists aim to unlock the full potential of polycatenated RFs in areas such as nanotechnology, materials science, and beyond. In this context, Liu’s research group has made a significant discovery regarding the self-assembly of ligands and its impact on the structural complexity of honeycomb motifs [92]. By examining the self-assembly of a rigid and planar ligand, the researchers were able to observe the formation of flat hexagonal honeycomb motifs, which further developed into undulated 2D layers. Interestingly, this undulation phenomenon was not observed in 2D layers constructed from a closely related, yet nonplanar ligand. This observation highlights the profound structural consequences that arise from even a slight torsion in the ligand. The structural changes induced by the ligand's torsion led to the generation of three polycatenated HOFs with unparalleled complexity. These HOFs exhibited unique stepwise adsorption behaviors under specific pressures, stemming from the intricate movement between interwoven hexagonal networks. Moreover, the HOFs demonstrated remarkable chemical stability, underwent phase transformations, and displayed a preference for adsorbing aromatic compounds. The findings presented in this research offer valuable insights into the self-assembly behaviors of HOFs, expanding our understanding of their formation and properties. The implications of these findings extend to the rational design of HOF materials for various practical applications. By harnessing the knowledge gained from this study, scientists can explore innovative strategies to engineer RF-NPs with tailored structures and functionalities. Recently, Cong's research team [93] reported a study on a fluorescent polycatenated reticular framework (MEP-HOF). This framework stands out due to its carefully designed macrocyclic building blocks, which enable the formation of supramolecular interactions and the creation of 3D polycatenated networks with excellent regenerability. Furthermore, the framework exhibits fluorescent properties that make it an effective sensor for aqueous nitrobenzene, with an impressively low detection limit. These remarkable findings not only contribute to the diversity of functional HOFs in terms of their topology but also serve as an inspiration for further exploration of macrocycle-based supramolecular materials in future studies. In addition, Chen et al. [94] conducted a study where they successfully synthesized two distinct polycatenated MOF materials, each containing a different metal center. These materials displayed significant framework phase transitions when the solvents were removed, and showcased a unique temperature-dependent adsorption and desorption behavior toward CO_2_ around room temperature. Importantly, a noteworthy finding from their research was the substantial impact of changing the metal ion within the MOF structure on its dynamic sorption properties. This observation holds promise for gaining fresh insights into the development of novel MOFs that can be employed in temperature and pressure-driven processes for the capture and storage of CO_2_.

## RF-NPs Synthesis

### Various Methods for Synthesizing RF-NPs: An Overview of the Key Routes

**Fig. 10:**
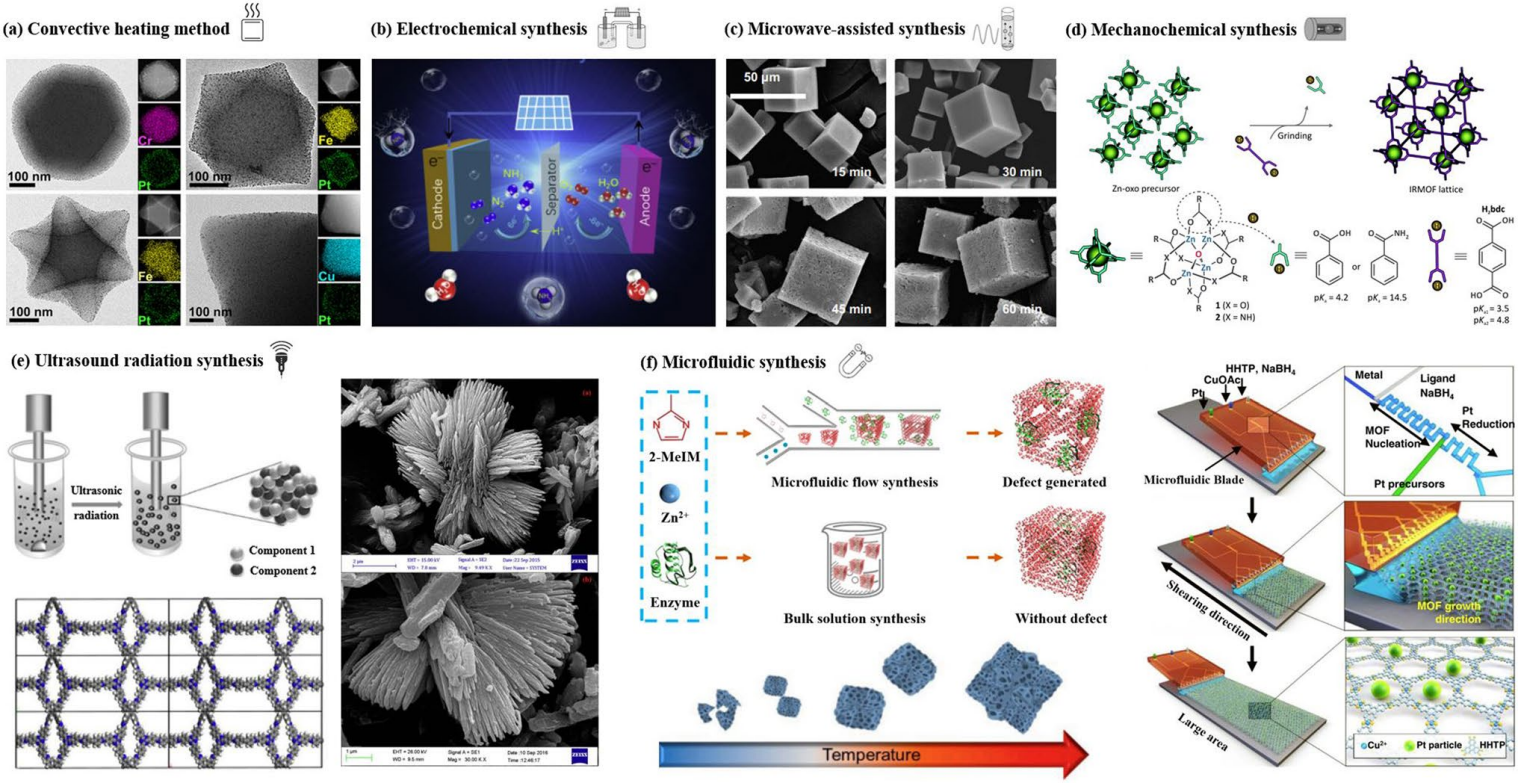
Overview of synthesis methods for RF-NPs. Direct synthesis approaches using: **a** the conventional solvothermal technique (reproduced with permission from Ref. [103]. Copyright 2021, Chinese chemical Society), **b** electrochemical method, **c** microwave-assisted synthesis route (reproduced with permission from Ref. [104]. Copyright 2011, Royal Society of Chemistry), **d** mechanochemical strategy (reproduced with permission from Ref. [105]. Copyright 2015, Royal Society of Chemistry). **e** Schematic illustration of ultrasonic system used in RF-NPs synthesis and FE-SEM data of MOFs structures using the Sonochemical technique [106, 107]. Copyright 2022, Springer Nature, 2018 Elsevier Inc. **f** Schematic illustrating the distinctions between microfluidic laminar flow synthesis and bulk solution synthesis techniques in the creation of host–guest composites using RF-NPs [95, 108]. Copyright 2020, American Association for the Advancement of Science and 2021, Springer Nature

**Fig. 11:**
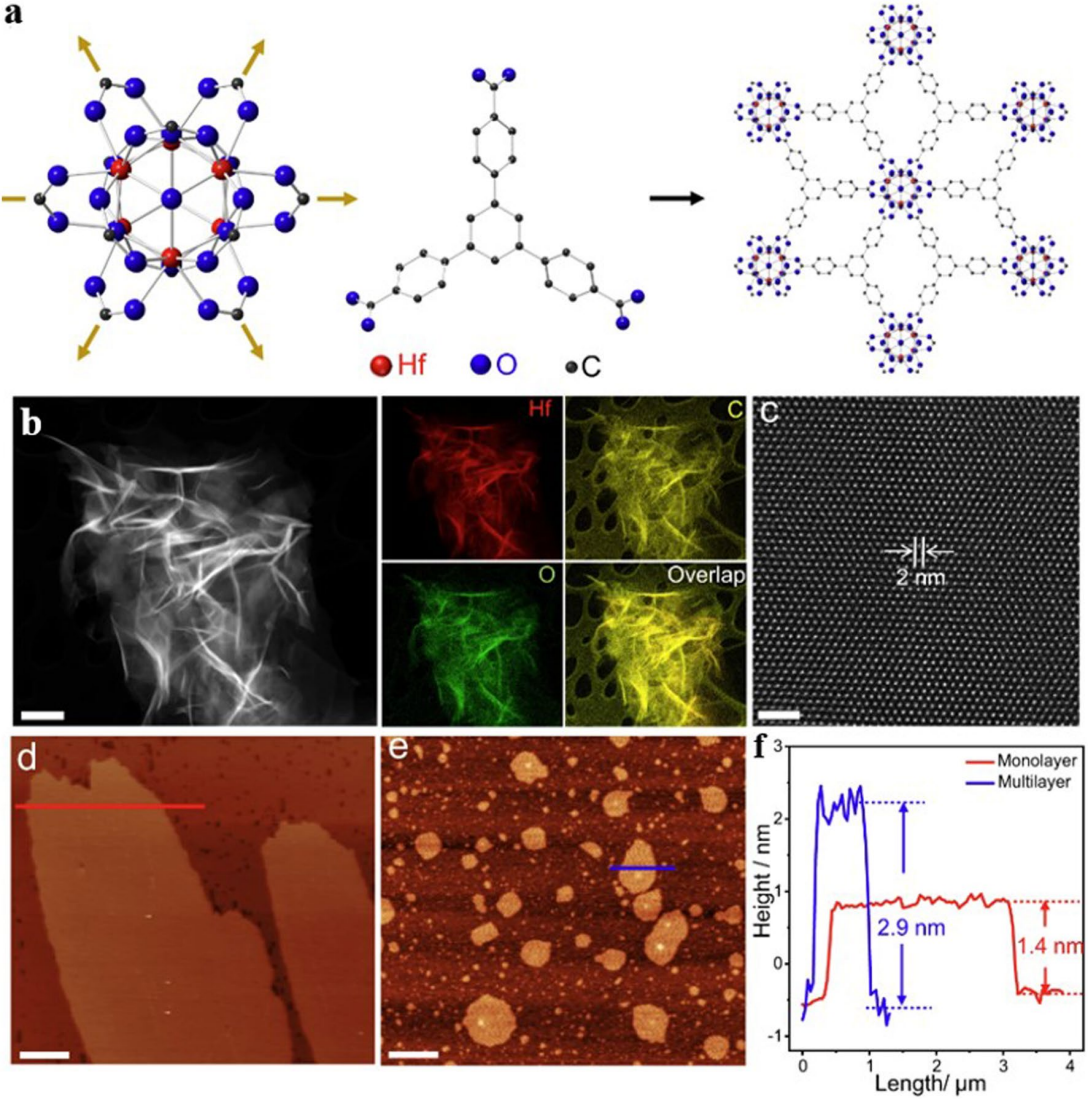
Recent synthesis of a metal–organic layer using solvothermal method. **a** Outlines the process of building a metal–organic layer using 6 connected Hf-clusters and 3 connected benzene-1,3,5-tribenzoate ligands. The connectivity between the two components is indicated by yellow arrows in the schematic. **b** Displays a representative HAADF-STEM image, along with the corresponding elemental maps. The elemental maps were generated using Super-X EDS at cryogenic temperature. **c** Exemplary high-resolution HAADF-STEM image of MOLs displays a hexagonal pattern of clusters arranged in a regular manner. **d, e** Metal–organic layer topography with distinct regions was analyzed using tapping-mode atomic force microscopy (AFM), resulting in height profiles along **d** a red graph in image and **e** a blue one in image. The resulting data is presented in **f**. Reproduced with permission from Ref. [102]. Copyright 2022, Springer Nature

The synthesis of reticular materials has gained significant interest as it offers an extraordinary opportunity to generate a wide array of visually captivating architectures, holding immense potential for valuable applications across numerous disciplines linked to porous frameworks (Table 1). Different synthesis methods are necessary for the production of distinct POFs due to the potential for the formation of different reticular frameworks from the same reaction mixtures [95]. The use of distinct synthesis methods has the capacity to make a substantial impression on various aspects of these materials production, including reaction time, yields, particle size and morphology, and the suitability of the POFs for large-scale implementation [96–100]. Figure 10 provides a comprehensive overview of the diverse methodologies that have been used over the past quarter-century to synthesize porous reticular frameworks. Figure 10a depicts a widely used conventional method for synthesizing materials called solvothermal/hydrothermal synthesis. This approach involves a self-assembly process where the crystals of RF-NP are grown from solutions containing metal node sources and organic linkers. The reaction takes place under high temperatures, which are higher than the solvent's boiling point, and high-pressure conditions within closed vessels [101]. For instance, Peng et al. [102] have recently conducted research into a detailed synthesis of the growth processes and intricate local structures exhibited by MOFs using a solvothermal synthesis route. Their approach involved using a combination of liquid-phase transmission electron microscopy, cryogenic electron microscopy, and electron ptychography, as shown in Fig. 11. Through their experiments, they have uncovered a complex, multistep formation process for MOFs. In the initial stages, precursor clusters undergo formation within a solution, subsequently interacting with ligands to generate amorphous solids. Following this, interaction between the precursor clusters and linkers leads to the formation of a complex, which subsequently undergoes self-assembly to generate organized crystalline sheet, while further expansion and development occur through subsequent growth stages transpires through the incorporation of clusters at the periphery. The utilization of advanced imaging techniques enabled the researchers to discern a diverse array of characteristics within the MOFs, including absent clusters, dislocations, loop and planar surface terminations, as well as ligand connectors. These insightful observations have contributed significantly to our understanding of strategies for controlling MOF crystal morphology, defect engineering, and surface modification. This research could have significant implications for the design and synthesis of novel MOFs, using the solvothermal technique, offering new avenues for exploration and development in this field.

**Table 1:** General overview of synthesis methods for RF-NPs

Synthesis method ^a^	Advantages	Disadvantages	Examples of RF-NPs^b^	Synthesis conditions		References
				Solvent ^c^	Conditions	
HS	Simple operation process Crystal growth can be controlled Complete crystal growth of RFNPs	High energy consumption Long reaction time Morphological control challenges Produce the RFNPs materials as powders, which may limit their applications under certain circumstance	MOF-5 HKUST-1 Fe-MIL-100 ZIF-8 UiO-66 SOF-7 TPT-COF-1 TPT-COF-2	DMF/chlorobenzene H_2_O/EtOH H_2_O (add HF + HNO_3_ aq.) DMF DMF DMF EtOH/CH_3_COOH Dioxane/Mesitylene/CH_3_COOH	120 °C, 24 h 180 °C, 12 h 150 °C, 6 days 85 °C, 72 h 120 °C, 24 h 90 °C, 72 h 120 °C, 72 h 120 °C, 72 h	[54] [120] [121] [59] [122] [123] [124] [124]
MW	More energy efficient Produce a better temperature profile Ease of controlling reaction conditions Higher synthesis rate and shorter reaction times	Obtaining single crystals is highly challenging Challenging to implement in industrial settings	MOF-5 COF-5 MIL-101(Fe) TpPa-COF MIL-100(Cr) IRMOF-1 ZIF-8 Co-MOF-74	NMP Dioxane/Mesitylene DMF Dioxane/Mesitylene DMF DEF H_2_O DMF/EtOH/H_2_O	105 °C, 30 min, 800 W 100 °C, 20 min, 200 W 150 °C, 10 min 100 °C, 60 min 179 °C, 3 h 25 s, 150 W 120 °C, 30 min 130 °C, 60 min, 300 W	[125] [126] [127] [128] [128] [129] [130] [131]
MC	Green and sustainable approach Solventfree Faster reaction rates Can be easily scaled up for largescale production	Less control over crystallinity of RFNPs Limited precursor compatibility Single crystal acquisition for diffraction studies is challenging	Cu(INA)_2_ ZIF-8 ZIF-4 Cu_3_(BTC)_2_ (HKUST-1) TpPa-1 (MC) TpPa-2 (MC) PFC-1 PFC-76-NH_2_	No solvent DMF DMF No solvent/MeOH Dioxane/Mesitylene/ SR No solvent/SR H_2_O/THF/dioxane H_2_O/THF/MeOH	25 Hz, 10 min 30 Hz, 5–60 min 30 Hz, 5–60 min 25 Hz, 15 min RT, 30 min RT, 60 min 85 °C, 12 h 85 °C, 12 h	[119] [132] [132] [133] [134] [135] [136] [136]
EC	Ability to tailor the electrochemical conditions and select suitable precursors Short synthesis time Facile synthesis of RFNPs Easy to control and mild reaction conditions	Limited availability of precursors Electrolyteprecursor incompatibility can cause adverse reactions	HKUST-1 ZIF-8 MIL-100(Fe) MIL-53(Al) NH_2_-MIL-53(Al) TpEB Eu@HOF-TCBP Cu_3_(HHTP)_2_	H_2_O/EtOH MeOH H_2_O/EtOH H_2_O/DMF H_2_O/DMF EtOH DMF/ EtOH H_2_O/ EtOH	50 °C, 25 V, 25 s RT, 0.7 mA, 20 min 110–190 °C, 0–20 mA KCl, 90 °C, 10 mA KCl, 90 °C, 10 mA RT, 50 V RT, 90 V, 5 min RT, Ag/AgCl as RE	[137] [138] [139] [114] [114] [116] [140] [141]
SC	Can achieve homogeneous distribution of nucleation sites Can be used to isolate pure phase Can facilitate the formation of welldefined and highly crystalline RFNPs Synthesis time is very short	Limited scalability Obtaining consistent and reproducible results can pose challenges Cost considerations	MOF-5 HKUST-1 Mg-MOF-74 ZIF-8 IRMOF-9, IRMOF-10 COF-1, COF-5 sonoCOF(1–9) MA-BTC HOF, HOF-to-MOF	NMP DMF/EtOH/H_2_O DMF/EtOH/H_2_O (add TEA) DMF (add TEA + NaOH aq.) DEF Dioxane/Mesitylene Dioxane/Mesitylene/AcOH MeOH (add MA + BTC)	200 W, 30 min 60 W, 60 min 500 W, 60 min 500 W, 60 min 150–300 W, 60 min 500 W, 1–2 h 550 W, < 60 min 500 W, 2 h	[142] [143] [144] [145] [146] [147] [106] [148]

^a^*HS* hydro/solvothermal, *MW* microwave-assisted, *MC* mechanochemical, *EC* electrochemical, *SC* sonochemical synthesis

^b^*Tp* Triformylphloroglucinol, *IR* isoreticular, *INA* isonicotinic acid

^c^*DMF* N,N-dimethylformamide, *NMP* N-methyl-2-pyrrolidone, *DEF* N,N-diethylformamide, *BTC* 1,3,5-benzenetricarboxylate, *RT* room temperature, *SR* schiff base reaction, *THF* tetrahydrofuran, *EB* ethidium bromide, *HHTP* 2,3,6,7,10,11-Hexahydroxytriphenylene, *RE* reference electrode, *TEA* triethylamine, *NMP* N-methyl-2-pyrrolidone, *MA* melamine

In these chemical reactions typically require an input of energy, synthesizing RF-NPs usually occurs in a solvent at temperatures between room temperature and around 250 °C. Conventional electric heating is commonly used to introduce energy, where heat transfers through convection from a hot source, such as an oven. Alternatively, energy can be introduced through electric potential [109], electromagnetic radiation [110, 111], mechanical waves (ultrasound) [112], or mechanical means [105, 113] (Fig. 10b–e). While numerous synthetic approaches are advancing, the commercialization of RF-NPs remains a significant challenge. However, the application of electrochemical synthesis has emerged as a viable solution, employing a continuous principle for synthesizing RF-NPs. By utilizing this synthetic methodology, several RF-NPs, namely HKUST-1, Al-MIL-53-NH_2_, ZIF-8, Al-MIL-100, and TpEB have been effectively produced via polarization or electrodeposition [114–116]. Microwave-assisted synthesis of RF-NPs has emerged as a valuable technique, offering several benefits such as accelerated crystallization, precise control over phase and morphology, and uniform particle size distribution. Due to its cost-effectiveness, efficient energy utilization, and precise regulation of reaction conditions, the microwave synthesis method is widely regarded as the best approach for synthesizing RF-NPs [104]. Microwave irradiation enabled the successful synthesis of MIL-100, marking it as the pioneering RF to be effectively produced using this method [117]. As previously mentioned, COF-5 achieved the distinction of being the inaugural boronate ester COF synthesized, and it also became the first one obtained under microwave irradiation [118]. Moreover, to address challenges associated with solvents and achieve rapid reaction times while obtaining measurable yields, a mechanochemical synthesis approach was proposed for the fabrication of RF-NPs materials. Pichon et al. [119] succeeded in the mechanochemical synthesis of RF-NPs by utilizing a ball mill as a grinding apparatus to successfully fabricate [Cu(INA)_2_]. Over the past years, microfluidic flow reactors have gained significant popularity owing to their remarkable capability to offer precise control over reaction time and temperature. This level of control is particularly important in ensuring accurate crystallization conditions. Microfluidic reactors operate by passing reaction solutions constrained pathways, be it within tubular structures or on microfluidic chips, with diameters as small as a few hundredths of a micrometer (Fig. 10f). Using this key route, Hu et al. [95] successfully accomplished the fabrication of enzyme-incorporated metal–organic frameworks using microfluidic laminar flow methodologies. By employing gradient mixing on a chip, the continuous variations in MOF precursor concentrations resulted in the formation of structural imperfections within the synthesized materials. Consequently, this introduced a diverse range of pore sizes in the MOFs, enabling improved substrate accessibility to the encapsulated enzymes while simultaneously safeguarding their stability. Notably, the enzyme-MOF composites obtained through this approach exhibited significantly heightened biological activity compared to those prepared using conventional bulk solution synthesis techniques.

### Precise Manipulation of RF-NPs Crystals: Achieving Controlled Morphology and Size

**Fig. 12:**
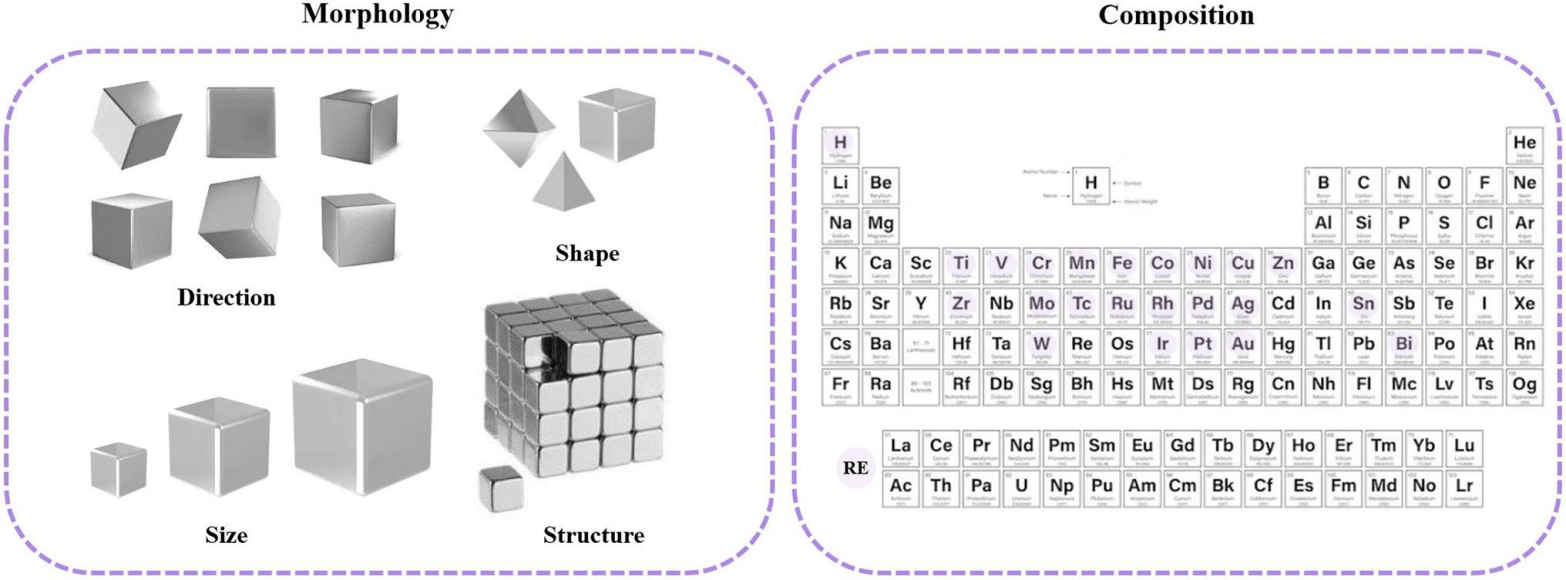
Typical modulation forms for RF-NPs crystals control

**Fig. 13:**
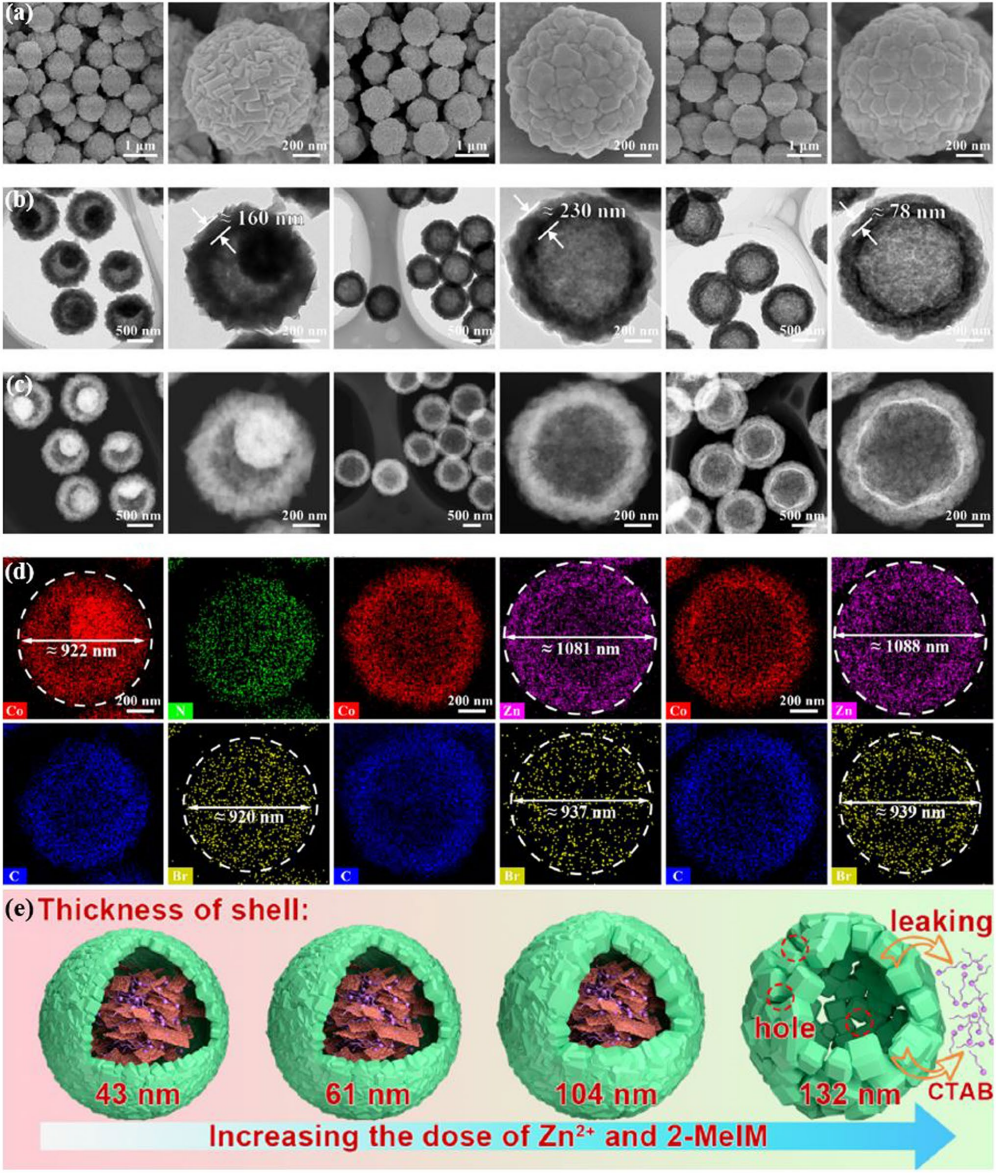
Analyzing the structural features of zeolite imidazole frameworks with different shell thicknesses. **a** SEM, **b** TEM, **c** HAADF-STEM, **d** EDS mapping images, and **e** schematic illustration of ZIF-8-HS obtained by Modulating 2-Methylimidazole and Zn^2+^ concentrations. Reproduced with permission from Ref. [152]. Copyright 2022, Elsevier Inc

In the field of constructing RF-NPs, the design of their structures and morphologies is undoubtedly crucial. However, an additional critical key to successful construction is the ability to control these structures and morphologies (Fig. 12). While designing the initial structure is important, it is equally important to be able to manipulate and fine-tune these structures to achieve the desired properties and functionality. Control over the structure and morphology of RF-NPs can be achieved through various methods such as the choice of precursor materials, reaction conditions, and post-synthesis treatments. For example, varying the temperature, pressure, and pH during synthesis can influence the size, shape, and surface chemistry of the particles [149, 150]. The performance of RF-NPs is primarily influenced by their composition, and therefore, it should be the foremost factor to consider [151]. Shen research team [152] conducted an investigation into how the thickness of a shell can affect the catalytic activity of nanoreactors. To accomplish this, they created a range of ZIF-8-HS nanoreactors with differing shell thicknesses by precisely controlling the concentrations of Zn^2+^ and 2-methylimidazole during the epitaxial growth process (Fig. 13a–d). As anticipated, the research group was successful in finely adjusting the thickness of the shell within a range of 43 to 132 nm with a comparable spherical shape and hollow structure, irrespective of the variations in their shell thicknesses, as illustrated in Fig. 13e. These findings prove the possibility to control the size of RF-NPs crystals with a high degree of precision, using only specific reaction conditions, which potentially lead to the development of more efficient and effective reticular materials. The ability to manipulate and control the size of nanoparticles is a critical aspect of nanotechnology research, and these results represent a significant step forward in this field.

**Fig. 14:**
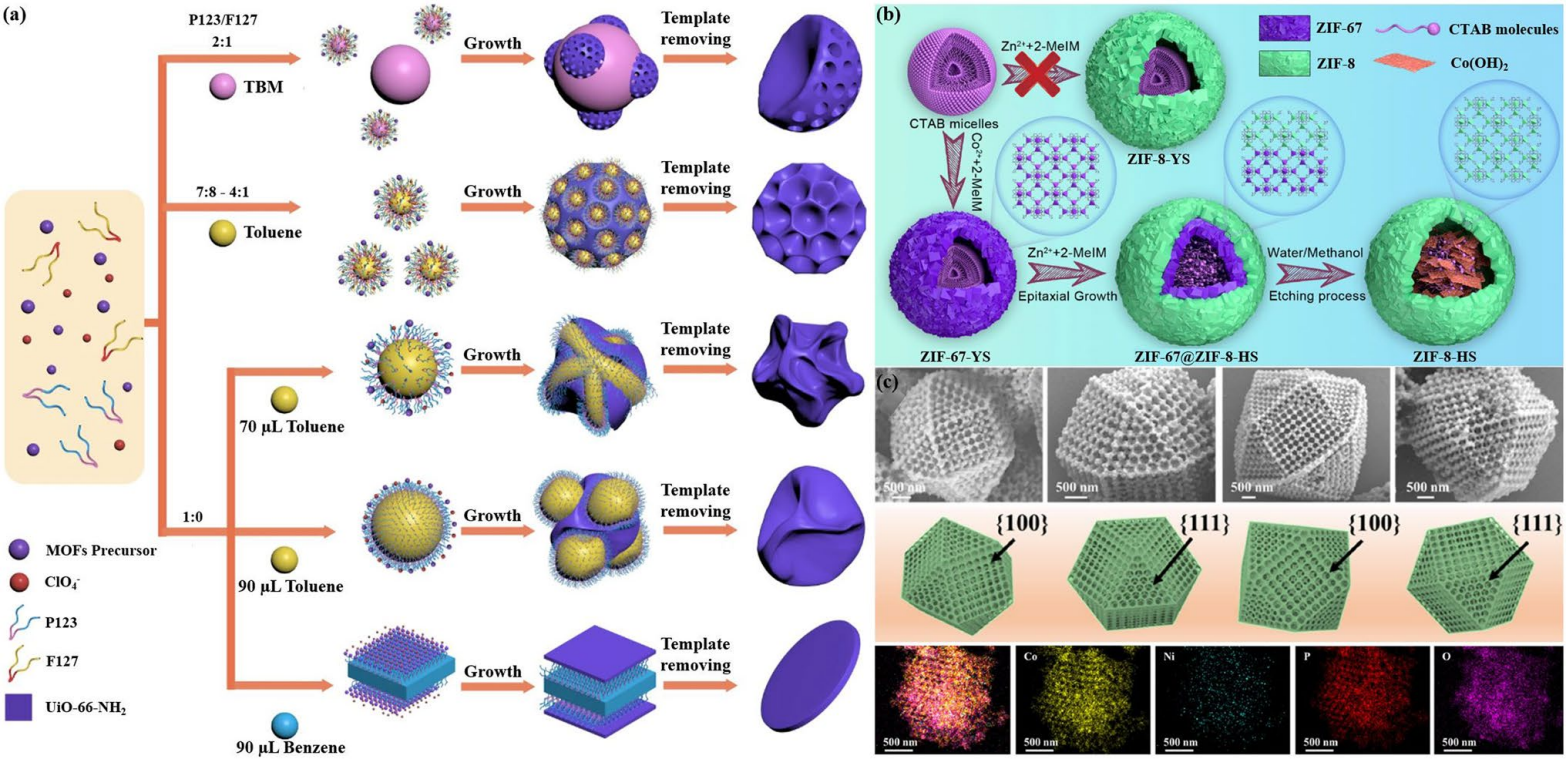
Schematic illustrations for **a** nanoemulsion-guided assembly strategy for forming hierarchical Zr-based MOFs with diverse architectures, **b** fabrication of size-tunable hollow nanoreactors (ZIF-8-HS) using Br-containing surfactants as templates. **c** SEM images of ordered macroporous Ni(II)/MSC-ZIF-67 single crystals from different perspectives, accompanied by EDS mapping using polystyrene (PS) template [152, 153, 162]. Copyright 2022 Springer Nature, 2022 Elsevier Inc

Moreover, soft templates can be used to form additional mesopores or macropores in POFs architecture, similar to how they are used in the fabrication of conventional porous materials like silica and metal oxides. By incorporating soft templates, POFs can be designed to have more complex and tailored pore architectures, which can be useful for various applications. Currently, the use of template-assisted methods involving soft materials in the synthesis of RF-NPs is primarily focused on ionic surfactants, nonionic surfactants, and emulsions. By carefully selecting these templates, the pore size of RF-NPs can be precisely customized and optimized. As one of the highly favored approaches for synthesizing RF-NPs with diverse structures, emulsion-driven approaches have been utilized to synthesize well-designed RF-NPs. Ke et al. [153] put forward an original methodology employing salt-assisted nanoemulsion-guided assembly for the seamless progression of hierarchical Zr-based MOFs' architectural transitions. This approach involves regulating the morphology of the nanoemulsion by adjusting the proportion of dual-surfactant and the quantity of compatible hydrophobic substances incorporated. By doing so, MOFs with diverse structures, including concave mesoporous particles resembling bowls, nanospheres with dendritic branching, particles resembling walnuts in shape, nanosheets with crumpled morphology, and nanodisks with distinct geometries can be assembled, as shown in Fig. 14a. Notably, the dendritic nanospheres that have been created exhibit expansive and porous mesochannels, which render them exceptionally suitable frameworks for simultaneously immobilizing coenzymes and their corresponding enzymes. This unique characteristic enables the convenient and localized regeneration of NAD^+^ in a heterogeneous environment. This innovative strategy provides a promising avenue for the exploration of sophisticated hierarchical MOFs that can be applied in practical settings involving bulk molecules. Besides emulsions, ionic surfactants represent another viable approach to control the morphology and the pore size of RF-NPs. Figure 14b represents a schematic illustration for the fabrication of size-tunable hollow nanoreactors (ZIF-8-HS) using cetyltrimethylammonium bromide (CTAB) surfactant as a template. In this study, Qin et al. [152] introduced a pioneering strategy to enhance the effectiveness of CO_2_ capture by employing encapsulated Br-based cocatalysts within stable and reusable ZIF-8-based hollow nanoreactors (referred to as ZIF-8-HS). Their method entails the controlled growth of a precisely regulated ZIF-8 layer onto hollow ZIF-67 spheres, which are initially synthesized using Br-containing surfactants as templates. Subsequently, the unstable ZIF-67 shell is selectively etched. The Br-containing surfactants integrated within the ZIF-8-HS function as efficient cocatalysts, facilitating the activation of substrates. Additionally, the microporous ZIF-8 shell supplies an abundance of intrinsic active sites and creates a confined, homogeneous environment for the cocatalysts. Remarkably, ZIF-8-HS showcases remarkable activity, exceptional size selectivity, and outstanding recyclability in the CO_2_ fixation reaction with epoxides. This approach harnesses the unique advantages of both homogeneous CTAB and heterogeneous ZIF-8, amplifying their respective strengths in this reaction. In addition, nonionic surfactants, which include procurable from commercial sources, amphiphilic block copolymers such as P123 and F127, belonging to the pluronic poly(ethylene oxide)-poly(propylene oxide)-poly(ethylene oxide) family, have garnered significant attention due to their versatile properties and diverse utility (Fig. 14a), have been also employed as soft templates for the synthesis of reticular framework nanoparticles with tunable pore structures and large pore sizes [154, 155]. For instance, Li et al. [156] described a new method for synthesizing metal–organic frameworks with ordered mesoporous structures that have uniform pore sizes of up to 10 nm and thick microporous walls. The process involves using triblock copolymer templates and Hofmeister salting-in anions to promote nucleation and in-situ crystallization of MOFs in an aqueous environment, resulting in microcrystals with large, periodically arranged mesopores. These MOFs have improved mass transfer capabilities due to their large-pore channels and robust crystalline structures, making them ideal nanoreactors for digesting biogenic proteins with high efficiency. This approach could serve as a guide for designing new mesoporous POFs with diverse compositions and functions using nonionic surfactants, opening possibilities for their use in applications involving biomacromolecules. In conjunction with these outcomes, the Gu research group [157] has managed to create HMUiO-66(Ce), a specific variant of cerium-based MOFs, demonstrates the presence of expansive channels featuring macroporous structures with sizes of up to 100 nm. The walls of these macropores exhibit a thickness range of approximately 11–12 nm. This unique characteristic of HMUiO-66(Ce) allows for enhanced permeability and accessibility within the material, enabling potential applications in various fields [158–161]. They achieved this through use of a microemulsion-guided assembly strategy, where they formed a columnar microemulsion with the help of surfactants P123 and F127. This new strategy allowed them to uncover the formation of desired structures influenced by the interplay between the assembly mechanisms of spherical stacking and epitaxial growth modes, which they hope will provide a useful conceptual guideline for designing a wide range of POFs. Furthermore, through their investigation, the researchers made a notable discovery regarding HMUiO-66(Ce): the presence of abundant Ce-OH sites on the macroporous walls and the unhindered mass diffusion within the macroporous channels. These distinct characteristics render HMUiO-66(Ce) an excellent candidate for mimicking nucleases, especially in the hydrolytic cleavage of robust phosphodiester linkages found in diverse DNA molecules. This finding underscores the potential of HMUiO-66(Ce) in applications related to DNA manipulation and opens exciting prospects in the field of biotechnology.

Despite the efforts made in the development of super-controlled structures for well-defined purposes, the comprehension of how the structure of RF-NPs influences their performance is still in its early stages. Consequently, there is an urgent demand for comprehensive research to gain a profound understanding of RF-NPs, which is crucial for designing multi-level architectures and exploring their potential applications in various fields. Exploring the mysteries of RF-NPs holds the key to unlocking their full potential, and could lead to exciting new advancements in nanotechnology.

## Advanced Characterization of RF-NPs

**Fig. 15:**
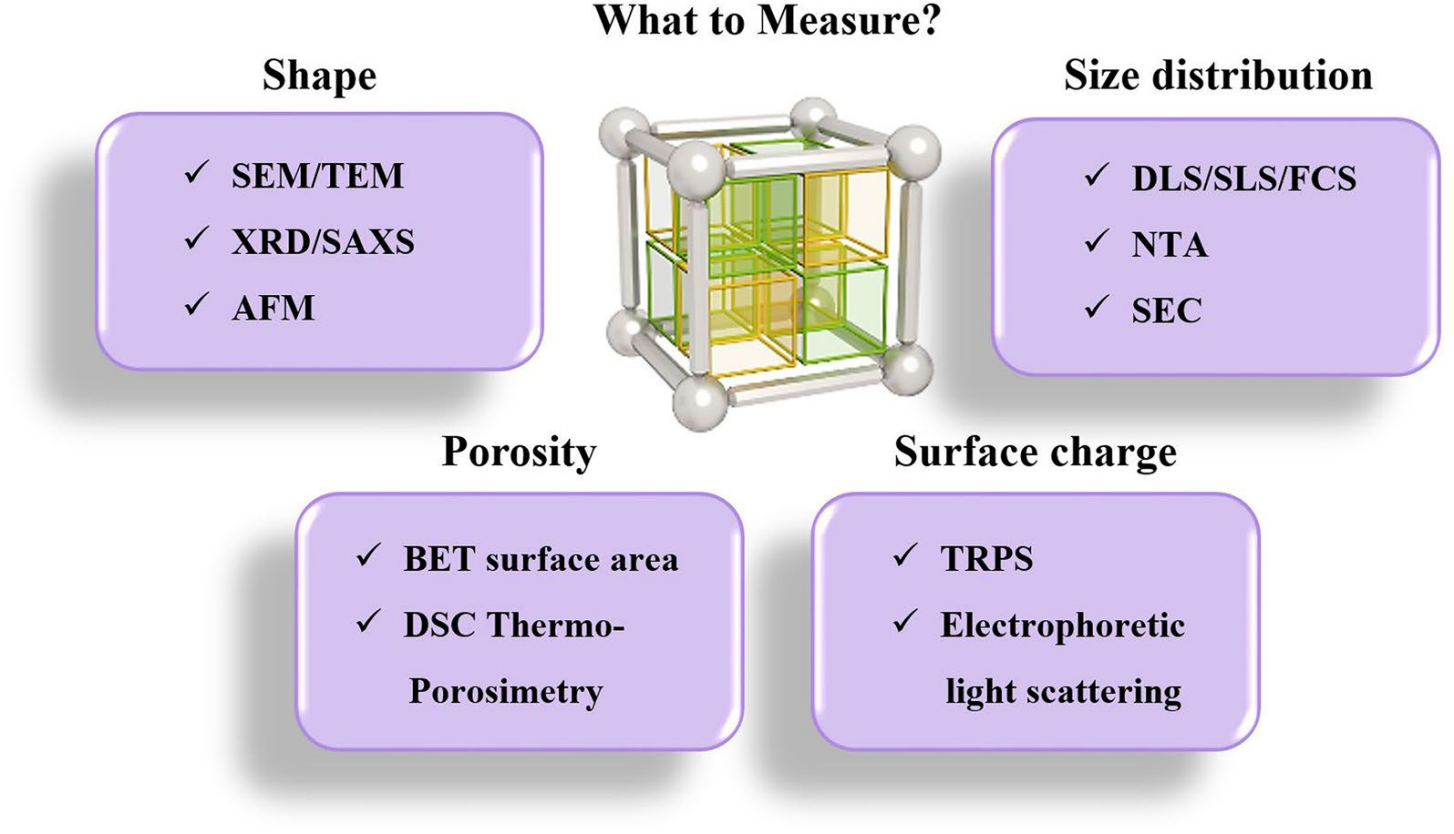
Survey of various analytical approaches for assessing the morphology, size distribution, porosity, and surface charge of RF-NPs

The synthesis of RF-NPs typically results in a polydisperse product that is highly sensitive to even minor changes in the reaction mixture [136, 163]. A multitude of factors can affect the outcome, leading to variations in the properties related to the physical and chemical characteristics of reticular frameworks. Within this section, our focus shall be directed toward providing a comprehensive summary of the most advanced techniques currently available for analyzing the configuration, dimensions, dispersion of proportions, electrical charge on the surface, and porosity of RF-NPs (Fig. 15).

**Fig. 16:**
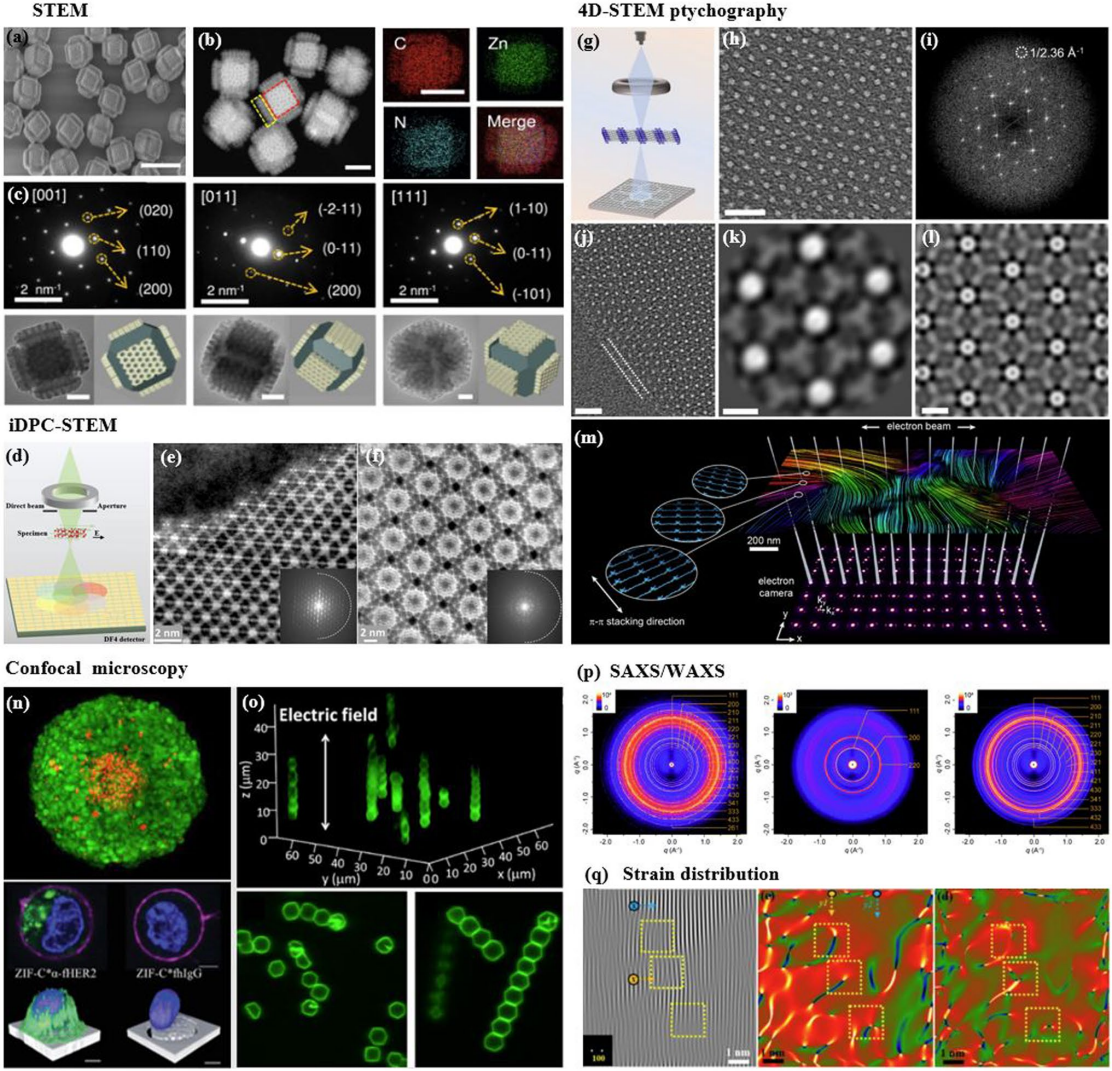
Advanced characterization of RF-NPs. **a-c** Characterization of MOF p-ANHs nanoparticle using **a** SEM, **b** HAADF-STEM with EDX Elemental Mapping, and **c** TEM imaging in zone axis analysis along [001], [011], and [110] with their corresponding models and SAED patterns (reproduced with permission from Ref. [187]. Copyright 2023, Springer Nature). **d**–**f** Atomic-level characterization of RF-NPs through iDPC-STEM imaging: d experimental setup for iDPC-STEM, **e**, **f** high-Resolution iDPC-STEM images and their corresponding FFT patterns of UiO-66 and MIL-101 respectively (reproduced with permission from Ref. [188]. Copyright 2020, Springer Nature). **g**–**m** 4D-STEM method: **g** schematic illustration for 4D-STEM electron ptychography imaging, **h** high-Resolution ptychographic reconstruction of RF-NP using 4D-STEM data, **i** Fourier transform intensity visualization of reconstructed phase image obtained via ptychography, **j** detailed surface/interface phase image of RF-NP obtained via ptychography reconstruction. The nanoscale ligand layer is highlighted with a white dashed line, **k** averaged image of 70-unit cells demonstrates clustered structure with interconnected ligands in the reconstructed phase image, **l** ptychographic reconstruction of a simulated dataset with four layers for comparative analysis, **m** illustration of focused beam scanning process over organic nanoparticles [102, 174]. Copyright 2022 Springer Nature, 2021 American Chemical Society. **n**, **o** 2D and 3D confocal microscopy imaging of n zinc-based MOF presented in either 2D (top) or 3D (bottom), **o** RD-ZIF-8 particle alignment in the direction of 1 MHz electric field using confocal microscopy [189, 190]. Copyright 2021, John Wiley & Sons Ltd., 2013 American Chemical Society. **p** Given information pertains to three 2D small-angle X-ray scattering (SAXS) images for MOF-217, activated MOF-217, and 28% Im-in-MOF-217. In each image, the blue circles represent the experimental data, while the red lines represent the calculated data (reproduced with permission from Ref. [191]. Copyright 2020, Royal Society of Chemistry). **q** Strain distributions of exy and exx in the (110) planes of NiMn-MOF. The analysis of these strain distributions aims to provide a better understanding of the structural properties of the material, particularly with regard to how the crystal lattice of the NiMn-MOF is deformed along the (110) planes (reproduced with permission from Ref. [192]. Copyright 2022, Elsevier Inc.)

RF-NPs shape and morphology are commonly studied using high-resolution microscopy techniques (Fig. 16a–c). This allows for analysis of individual particles at sub-nanometer scale in a two-dimensional plane and provides insight into the interconnection between the underlying crystal lattice and the overall external form through electron diffraction [164, 165]. However, these methods require an oxide-free, electrically conductive surface and are performed in a vacuum. Atomic Force Microscopy (AFM) in tapping mode offers the ability to accurately assess the three-dimensional configuration of nanoparticles present in a solution. Other techniques such as Small-Angle X-ray Scattering (SAXS) and Analytical Ultracentrifugation (AUC) can also be employed to characterize the shape of nanoparticles when they are immersed in a liquid medium [166]. The integrated differential phase contrast (iDPC-STEM), shown in Fig. 16d–f, is a STEM technique that utilizes incident electrons more efficiently than other Dark-field electron microscopy, as widely employed technique. This increased efficiency, in tandem with the integration process, yields improved signal-to-noise ratio under low-dose conditions, which is particularly essential for electron beam-sensitive materials [167]. Researchers have successfully used iDPC-STEM to image various reticular materials, such as MOFs, COFs, and HOFs [168–171]. Shen et al. [172] reported a groundbreaking advancement in imaging beam-sensitive materials using low-dose imaging and an integrated differential phase contrast technique. Specifically, they successfully imaged the beam-sensitive material using a Cs-corrected imaging setup in scanning transmission electron microscope. The resulting images provided a high-resolution view of the coordination between the framework's Cr nets and ligands, with distance of approximately 1.8 Å. Furthermore, the iDPC-STEM technique allowed the researchers to explore and reveal the local structures of the MOF, including its surfaces, interfaces, and defects. These findings represent a significant step forward in the field, as they provide a valuable method for imaging various beam-sensitive materials at an ultra-high resolution. This approach may also pave the way for future research on defect and surface engineering of MOFs to achieve tailored functions. To gain a more advanced understanding of the properties of RF-NPs, a state-of-the-art technique known as four dimensional (4D)-STEM was utilized (Fig. 16g–m). This technique involves using a tiny probe with a size of approximately 1 nm to examine a large geometric area of up to 1 × 1 mm^2^ in size [173]. The purpose of using this approach was to observe the local strain behavior and intricate electronic structure of the RF-NPs in greater detail. By employing this advanced characterization technique, researchers were able to obtain a more holistic comprehension of the characteristics exhibited by the RF-NPs. Should one seek a deeper understanding of the technical aspects of 4D-STEM, we highly recommend consulting two recently published review articles [174, 175]. These articles provide a comprehensive overview of the subject matter and delve into the intricacies of the underlying technology. Moreover, as porous materials effective for encapsulation, RF-NPs are also characterized using confocal microscopy is an essential tool allowing researchers to visualize and study the distribution, morphology, structure, and behavior of specific particles within the matrix material (Fig. 16n–o). Its high-resolution, non-destructive imaging capabilities make it an invaluable tool for the development of functional composite materials based on POF encapsulation. Besides the shape, the degree of porosity in RF-NPs is subject to the influence of various factors, including the size and volume of cavities, pore opening size, and the degree of interconnectivity within the POFs structure. These factors collectively determine the accessibility of the pore network, which can either be limited to surface pores or extend to all pores. The extent of porosity in RF-NPs can be determined through sorption studies using the Brunauer–Emmett–Teller (BET) method [176]. However, obtaining accurate results using this method requires a precise understanding of the crystal structure of the nanoparticles [177–179]. With similar dimensions, there are several methods available to analyze particle suspensions, each with its own advantages and limitations. The most appropriate method to use will depend on the specific characteristics of the particles being studied. For instance, for particles of different sizes, diffusion-based methodologies, including light scattering techniques such as LS (light scattering), SLS (static light scattering), and DLS (dynamic light scattering), along with fluorescence correlation spectroscopy (FCS) and differential centrifugal sedimentation (DCS), as well as size-exclusion chromatography (SEC), can be employed to separate particles based on their size [180]. However, for nanoparticles with a size above 30 nm, two complementary techniques have emerged as particularly useful. The first is nanoparticle tracking analysis (NTA), which can analyze particles ranging from 30 to1000 nm in size. The second is fluorescence-activated nanoparticle sorting (FANS), which can analyze particles larger than 100 nm [181, 182]. Furthermore, the surface charge of RF-NPs is a crucial factor in determining their colligative properties, which ultimately influence how they interact with solvents, electrolytes, and other nanoparticles. The surface charge can be quantified using a variety of techniques, such as tunable resistive pulse sensing (TRPS) [183, 184], electrophoretic light scattering (ELS) [185], and zeta-potential [186] measurements. By utilizing these techniques, the electric potential disparity between the charges affixed to the surface of the nanoparticle and the potential of the encompassing solution can be determined. The measurement of surface charge is essential in understanding the behavior of nanoparticles and can aid in the optimization of various nanotechnology applications. In brief, to comprehensively characterize nanoparticles for specific applications, several physiochemical properties need to be considered. The first step is to conduct a structural characterization using electron microscopy (EM) to precisely assess the dimensions, morphology, and variability of the particles. However, if the specimen cannot be manipulated in a vacuum environment, alternative methods such as AFM, DLS, and NTA should be used. Next, to determine the dimensions of the crystal, the degree of phase purity, and the overall level of crystallinity of the nanoparticles, PXRD should be performed and compared to theoretical data. Another critical step in the characterization process is to utilize gas sorption measurements, which play a vital role in evaluating the porosity and apparent surface area of the synthesized particles. These measurements provide valuable insights into the particle's porous structure and help determine its overall surface properties. For applications in liquids, such as drug delivery, it is crucial to the maintenance of stability in colloidal systems and the propensity for aggregation formation within an aqueous solution as a function of pH. This can be achieved through DLS or NTA. Finally, If the composition of nanoparticles is altered by modifying the building blocks, such as exchanging metal centers, modifying organic linkers, applying surface coatings, or introducing guest molecules via adsorption, a combined methodology comprising MS (Mass Spectrometry), ICP-OES (Inductively Coupled Plasma-Optical Emission Spectroscopy), and NMR (Nuclear Magnetic Resonance) techniques can be employed for characterizing the sample's purity, as well as quantifying the ratios of elements and linkers.

## Hybridization Strategies

### Single-Component MOF/COF Hybrid Materials

**Fig. 17:**
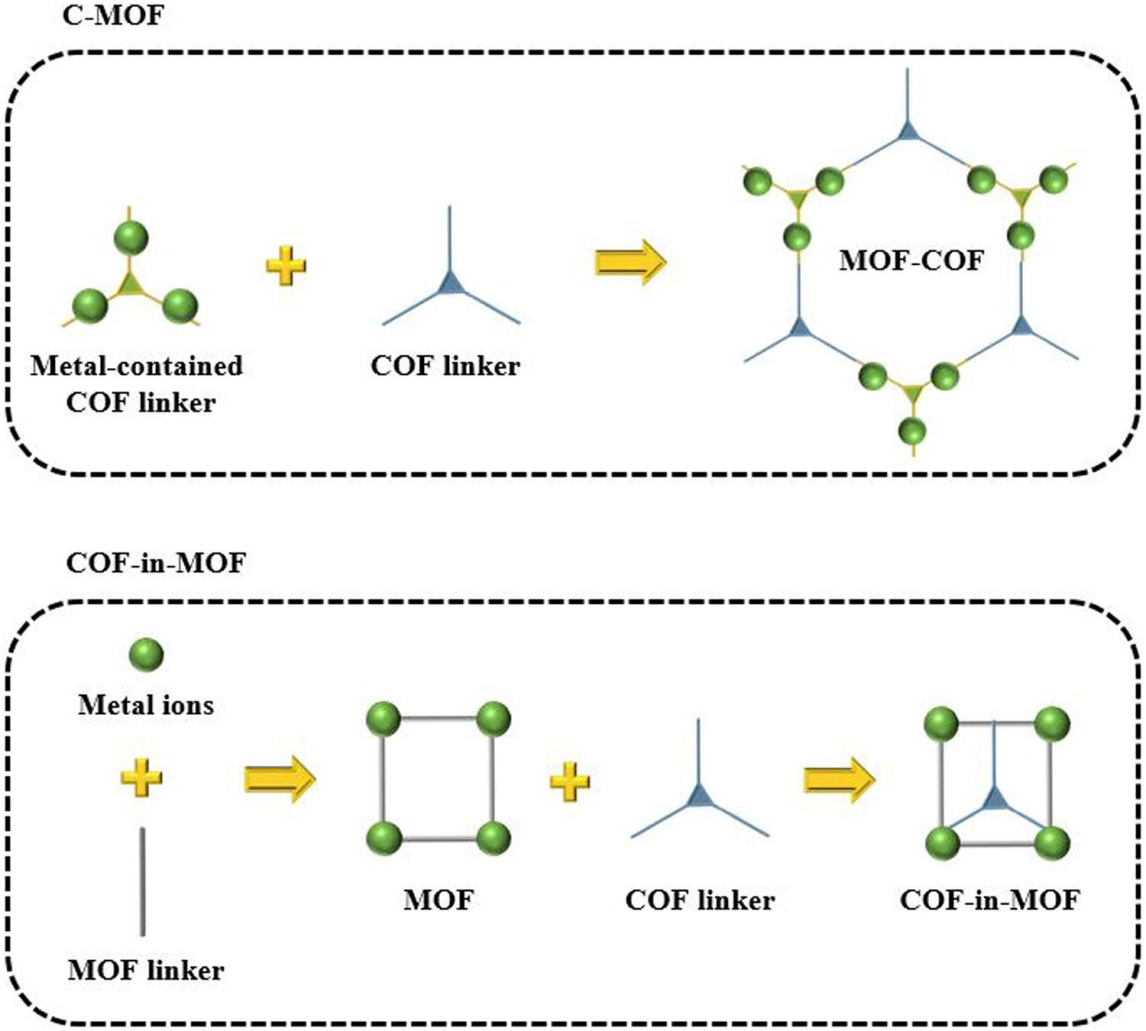
MOF/COF Hybrid Materials Consisting of a Single Component

**Fig. 18:**
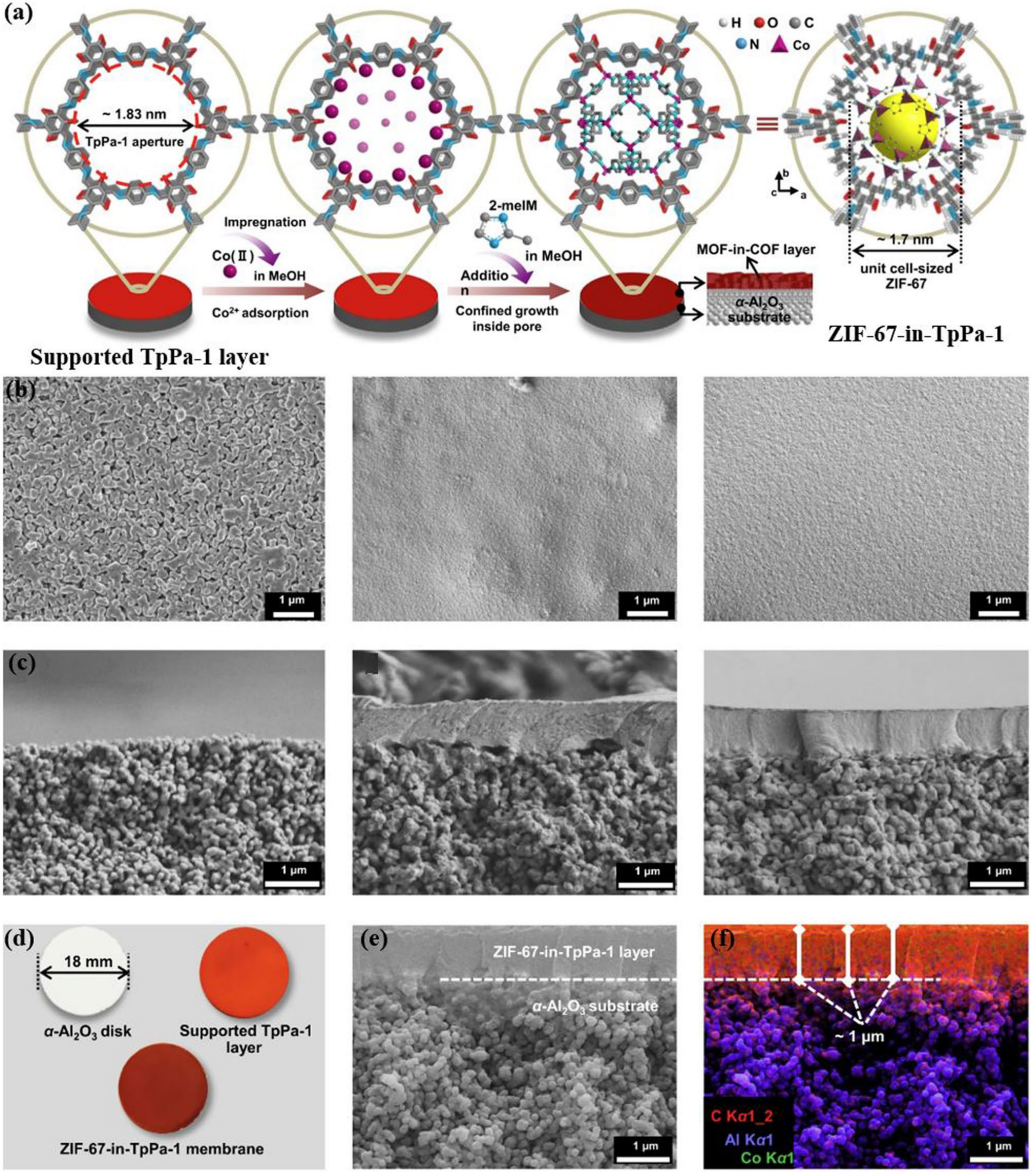
Single-Component MOF-in-COF hybrid materials. **a** Synthesis scheme of ZIF-67 incorporated into TpPa-1 membrane. **b, c** SEM images of porous α-Al_2_O_3_ substrate, TpPa-1 layer, and ZIF-67-in-TpPa-1 membrane **b** in top-view and **c** cross-section. **d** Optical images of the as-prepared membranes. **f** EDXS mapping and elemental distributions corresponding to **e**. EDXS mapping and its corresponding elemental distribution related to image (**e**). Reproduced with permission from Ref. [196]. Copyright 2021, Springer Nature

It is a well-known fact that COFs exhibit superior chemical and structural stability when compared to MOFs. This is primarily attributed to the presence of covalent bond formation within the COFs, which contributes to their robustness and durability. However, during the process of synthesizing COFs, the severe experimental conditions can lead to the breakdown of the MOF structure [193, 194]. As a result, it becomes imperative for researchers to determine the covalent bond orders and strengths of COFs to ensure the integrity and stability of the MOFs core. This is a significant challenge faced by most researchers and requires innovative approaches and techniques to overcome (Fig. 17). Aiming at this issue, Nguyen et al. [195] introduced the first C-MOF, known as MOF-901, which marked a pivotal milestone in the realm of reticular chemistry. MOF-901 was fabricated by merging the chemical properties of both metal and covalent organic frameworks, resulting in a novel material with unique characteristics. This breakthrough discovery opened new avenues for further research and innovation in this area, paving the way for the development of advanced materials with enhanced properties and capabilities. As another strategy, Fan et al. [196] proposed a novel approach called the MOF-in-COF concept, which involves the incorporation of a MOF within a supported layer of covalent organic framework for structural reinforcement (Fig. 18). These membranes showcase a unique network of micro/nanopores stemming from the arrangement of MOFs as a series of unit cells within the one-dimensional channel of two-dimensional COFs. Astonishingly, these MOF-in-COF membranes exhibit exceptional hydrogen permeance, and significantly enhance the selectivity of hydrogen separation compared to other gases. Notably, they outperform the upper limits defined by the Robeson plot for H_2_/CO_2_ and H_2_/CH_4_ separation. This superior performance stems from the synergistic amalgamation of precise size exclusion and rapid molecular transportation facilitated by the MOF-in-COF channels. Furthermore, the versatility of this design approach is demonstrated through the synthesis of diverse combinations of MOFs and COFs, resulting in robust membranes.

### Multicomponent MOF/COF Hybrid Materials

**Fig. 19:**
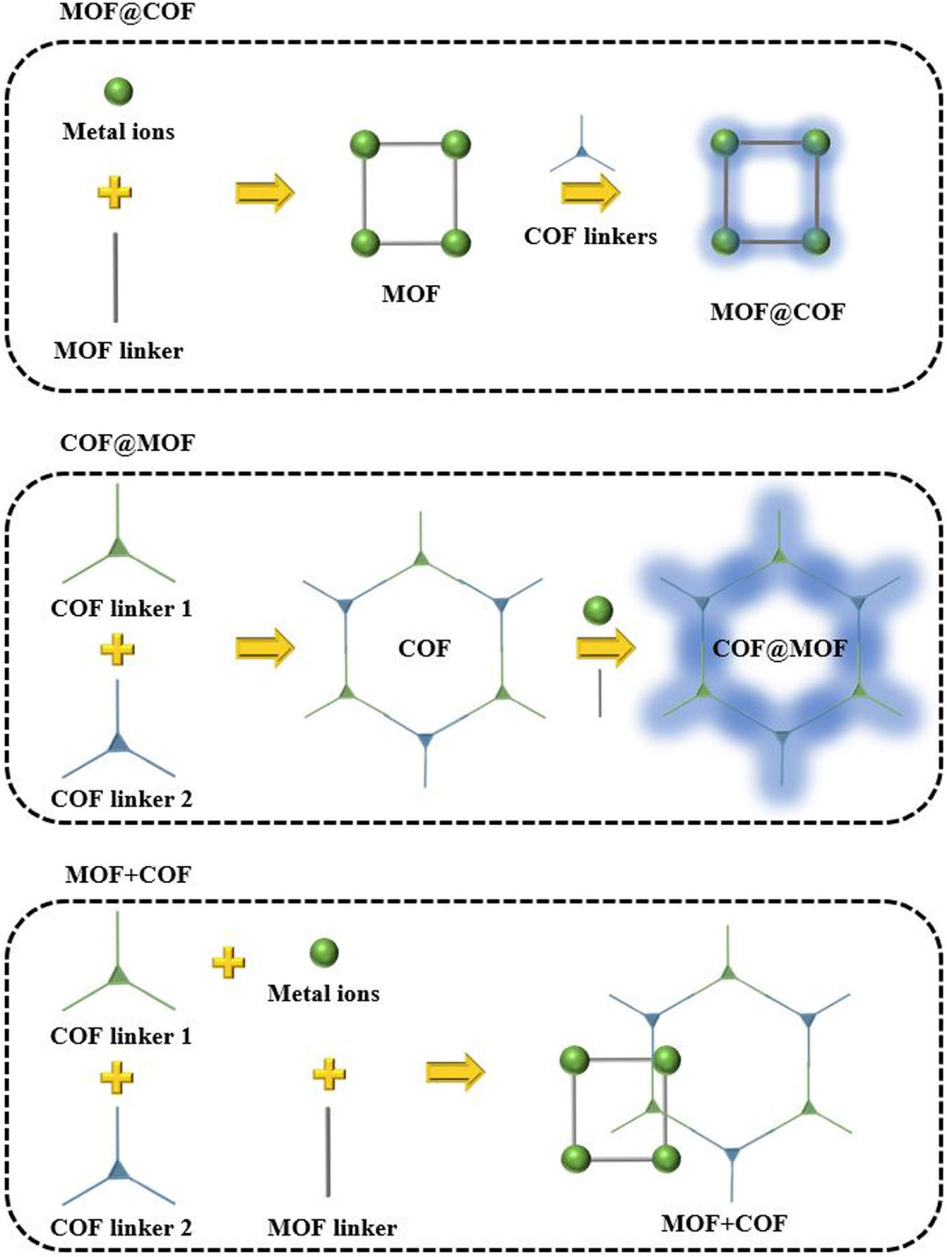
MOF/COF Hybrid Materials with Multiple Components

During the past few years, there has been a notable upsurge in comprehensive investigations and scholarly endeavors in developing various approaches to construct composites consisting of multi-component MOFs and COFs. The encouraging outcome of this research is the successful development of numerous MOF/COF composites. These composites can be primarily categorized based on the formation of either covalent or non-covalent bonds. The three primary composite formations are MOF@COF, COF@MOF, and MOF + COF, as depicted in Fig. 19. In this context, Peng et al. [197] presented an innovative methodology for synthesizing a new type of material MOF@COF core–shell hybrid material. The material is made by integrating MOF and COF, resulting in a high crystalline structure with a hierarchical pore system. The specific material they created, NH_2_-MIL-68@TPA-COF, was tested as a visible-light-driven photocatalyst for the degradation of rhodamine B, showing promising results. The authors suggest that this approach could be used to develop other MOF-COF hybrid materials for other applications.

The fabrication of composites consisting of COFs incorporated within MOFs has been achieved through multiple synthetic approaches, analogous to the methods used for the synthesis of MOF@COF composites. As a case in point, the successful integration of a stable MOF with a 2D COF by Zhang et al. has resulted in the synthesis of a novel hybrid material. This was accomplished by covalently anchoring the archetypal Zr-based MOF onto the surface of TpPa-1-COF. The resulting material exhibits high crystallinity, a porous framework, and a large surface area. When exposed to visible light irradiation, this new hierarchical porous material exhibits excellent performance in photocatalytic H_2_ evolution [198]. Moreover, the modular synthesis strategy has been also employed to prepare hybrid composites consisting of COF and MOF materials. This approach involves the sequential assembly of different building blocks in a modular manner to create complex composite structures. The resulting hybrid composites showcased a synergistic blend of the distinct properties exhibited by both COF and MOF materials, leading to enhanced performance in various applications. As a proof-of-concept, Feng et al. [199] expanded the application of modular synthesis beyond traditional materials to include framework materials, which connect metal- and covalent organic frameworks in a hierarchical manner using reticular chemistry. The method involves assembling fundamental units into complex designs, guided by the strength of coordination or covalent bonds. The researchers successfully produced a range of layered COF-on-MOF systems with intricate structural designs by combining diverse building blocks in a sequence-defined manner. These framework materials can be fine-tuned for spatial apportionment, composition, and functionality. The team's work represents the first report of COF@MOF architecture. The implementation of the modular synthesis strategy offers a rapid approach to discovering multicomponent framework materials. This strategy also enables the development of smart materials with remarkable tunability, thanks to the diverse range of inorganic or organic building units employed. Furthermore, it facilitates a predictable retrosynthetic route, streamlining the design and synthesis process.

In addition to core–shell composites formed between MOFs and COFs, there have been other types of MOF + COF composites that have been successfully constructed. These composites do not necessarily have a core–shell structure, but still involve the integration of MOFs and COFs to create novel materials with enhanced properties. In this vein, the research team led by Zou [200] has introduced a new approach that involves modifying RF-NPs hybrid materials through post-synthetic covalent modification. In this innovative method, the covalent triazine-based frameworks (CTFs) modified with benzoic acid (B-CTF-1) are covalently linked with MOFs based on Titanium and Zircunium clusters. The covalent bonding between B-CTF-1 and the MOFs facilitates the formation of a new hybrid material with combined properties from both components. The results of the study show that the hybrid materials exhibit excellent photocatalytic properties, with a hydrogen production rate of 360 μmol h^−1^ g^−1^. Furthermore, the hybrid materials formed through the covalent bonding of B-CTF-1 with MOFs exhibit higher stability compared to simple heterostructures of MOFs and COFs connected via Van der Waals forces. Researchers have attributed the enhanced photocatalytic performance of these MOF/COF hybrids to the formation of amide bonds between B-CTF-1 and MOFs. These amide bonds facilitate improved charge separation efficiency, leading to enhanced photocatalytic activity. Additionally, the amide bonds contribute to the overall stability of the photocatalyst, further enhancing its performance and longevity, as suggested by photoelectrochemical analyses and controlled experiments.

### Hybrid MOFs and HOFs: Unraveling the Potential of Synergistic Materials

Hybridization strategies for MOFs and HOFs have gained significant attention in recent research endeavors. The goal is to combine the unique properties and functionalities of both MOF and HOF materials to create novel hybrid materials with enhanced performance. Synthesis approaches for achieving this hybridization involve careful design and manipulation of the constituent components. One common strategy involves utilizing MOFs as a host matrix to support the in-situ assembly and growth of stable HOFs, resulting in core–shell hybrid structures. Surface functionalization of MOFs enables precise control over the growth and integration of HOFs, leading to well-defined interfaces and improved stability. By combining the structural diversity and porosity of MOFs with the hydrogen-bonding capabilities and functional groups of HOFs, researchers aim to achieve synergistic effects. These hybridization strategies pave the way for the development of advanced materials with tailored properties and functionalities, opening up new avenues for applications in different areas. In this context, Liu's research group [201] introduced a novel approach to develop an optimized functional core–shell nanostructure based on HOF. This composite structure consists of upconversion nanoparticles (UCNPs) and HOF (PFC-55), enabling efficient conversion of near-infrared (NIR) light into visible light through a process called RET (resonance energy transfer). Consequently, a synergistic platform that responds to NIR light for photothermal and photodynamic applications is achieved. This study encompasses three key innovations: (1) The successful synthesis and characterization of a porous HOF, possessing both photothermal and photodynamic capabilities, marking a pioneering achievement. (2) By employing a stepwise ligand-grafting technique known as the “bottle-around-ship” strategy, the research team successfully fabricates the first-ever HOF-based core–shell composite, introducing a novel synthetic approach for HOF-based composite materials. (3) The resulting composite not only inherits the unique properties of its parent materials but also establishes an effective RET pathway between the two components, thereby enabling the design of HOF composites with enhanced performance beyond that of individual components. The researchers anticipate that the findings presented in this study will open new avenues for functionalizing HOF materials and further enhancing their application potential. The researchers envision that the findings presented in this study will revolutionize the functionalization of HOF materials, unlocking new possibilities for their widespread application and impact. Moreover, the utilization of HOF nanoparticles in photocatalytic applications has been limited due to their inherent weaknesses in stability and activity. However, a potentially effective approach to enhance the photocatalytic performance of HOFs is to create a core–shell composite structure by incorporating stable HOFs into a specific nanoarchitecture. In this vein, Wang et al. [202] explored the utilization of surface-functionalized MOFs as a host matrix to support the in situ assembly and multisite growth of stable HOFs. Through this approach, a core–shell hybrid structure, NH_2_-UiO-66@DAT-HOF, was successfully synthesized. Remarkably, this newly developed core–shell nanostructure demonstrated excellent stability and outstanding photocatalytic performance. For instance, in the degradation of tetracycline, the optimized composite exhibited an apparent reaction rate constant 60.7 and 7.6 times higher than its parent materials, NH_2_-UiO-66 and DAT-HOF, respectively. This significant improvement in photocatalytic efficiency can be attributed to the broader visible-light absorption range compared to the individual parent materials, as well as the effective separation of charge carriers facilitated by the S-scheme heterojunction. Furthermore, it is worth noting that the photocatalytic efficiency of the resulting core–shell nanostructure remained high even after multiple cycles of application. The findings presented in this study introduce a universal approach for synthesizing MOF@HOF core–shell hybrids, highlighting their potential for diverse applications.

## Functionalization Strategies

### Inner Surface Modification: A Path to Tailored Material Functionality

The development of solid-state and materials chemistry hinges on the chemist’s aptitude to tailor the molecular architecture of porous materials with exquisite accuracy at the atomic level by incorporating functional moieties. In contrast to other types of nanoparticles, reticular nanoparticles offer a wider range of options for functionalization due to their versatility [203–206]. Additionally, the unique properties of their internal and external surfaces, along with the variations in the metal-linker composition, create an opportunity for exploiting multiple functionalization techniques within a single framework [207, 208].

**Fig. 20:**
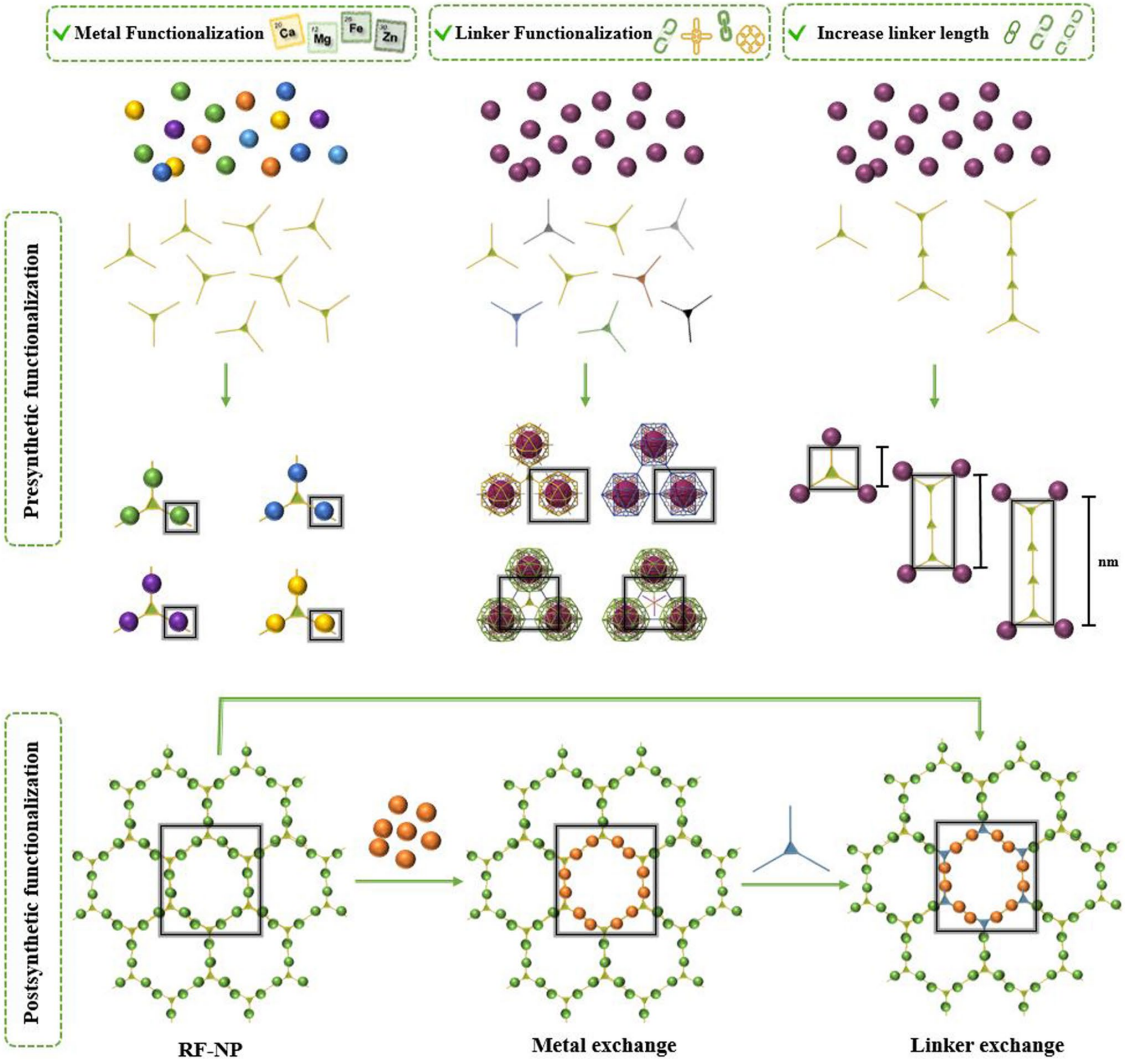
Overview of inner surface functionalization strategies

To enhance the molecular structure repertoire of reticular frameworks by functionalization, we can follow either of the two routes. The first method, known as the “Presynthetic approach”, involves the addition of functional moieties to the building blocks before the molecular backbone is formed, with an accuracy that can reach the atomic level (metal functionalization, linker functionalization, or by increasing linkers length). The second method, known as the “Postsynthetic approach”, also referred to as a secondary modification method, offers an alternative way to introduce functional groups into the structure, as illustrated in Fig. 20. This technique involves the addition of specific chemical moieties or functional units after the initial formation of the molecular backbone. By carefully selecting and incorporating these functional groups, the properties and performance of the resulting material can be tailored to meet specific requirements. This approach provides flexibility and control in fine-tuning the structure, enabling the customization of desired properties without altering the overall molecular framework.

When it comes to functionalize reticular materials using Presynthetic methods, the primary obstacle lies in incorporating functional groups without disrupting the scaffold formation process. RF-NPs consist of two distinct types of building blocks, namely, inorganic and organic building units [209–211]. While MOFs and ZIFs are formed using a combination of both inorganic and organic units, COFs and HOFs are solely constructed from organic units [12, 26, 212–215]. All these unique nanomaterials offer a unique opportunity for the synthesis of multifunctional designs due to their simple crystal engineering, which allows for the incorporation of various functionalities in both the inorganic and organic building units, in addition to their inherent porosity and size-dependent properties. This is made possible through reticular chemistry, which enables the efficient and uncomplicated synthesis of nanomaterials with diverse properties [216–220]. As a result, RF-NPs can be tailored to incorporate a variety of functionalities, making them highly versatile and adaptable for various applications.

### In Situ Encapsulation for Efficient Functionalization

**Fig. 21:**
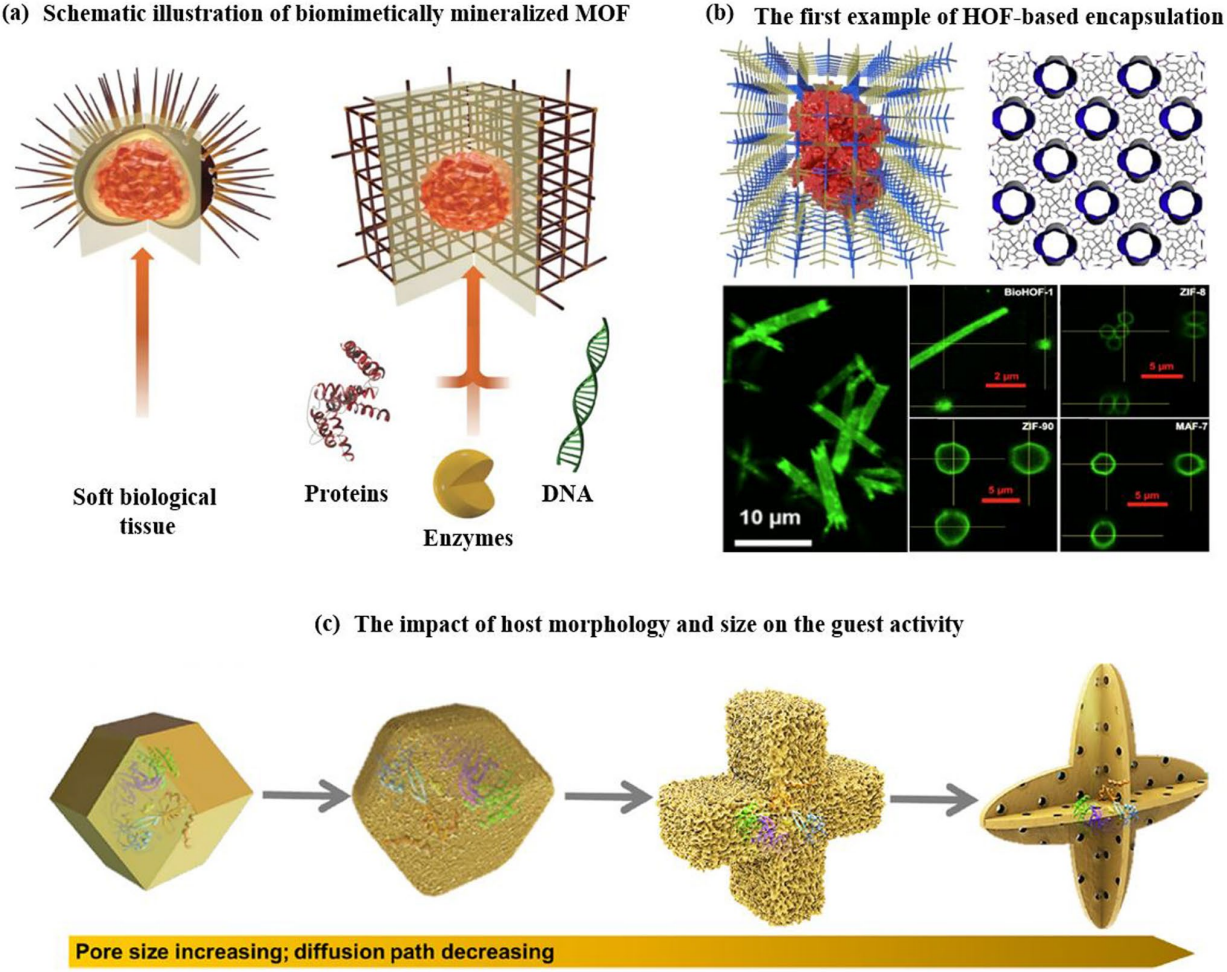
Host–Guest interactions. **a** Schematic illustration comparing between sea urchin as a living organism enclosed in a rigid, porous outer layer that is formed by the biological tissues within, and a bio composite made of a MOF, with a large biomolecule such as a protein, enzyme, or DNA molecule contained within the porous, crystalline structure of the composite (reproduced with permission from Ref. [239]. Copyright 2015, Springer Nature). **b** The first example of HOF-based encapsulation (reproduced with permission from Ref. [236]. Copyright 2019, American Chemical Society). **c** Schematic illustration demonstrating the implementation of glutamic acid-guided assembly approach for the alteration of the morphology of the host metal–organic framework transforming it from a 3D polyhedron to a 2D sheet. This tailored approach results in larger pore sizes and extended diffusion paths for substrates within the 2D sheet, which ultimately leads to enhanced guest activity. Reproduced with permission from Ref. [240]. Copyright 2022, Royal Society of Chemistry

Throughout history, humans have sought solutions to complex problems by studying nature and its various systems. The natural world has been a source of inspiration for inventions and innovations, such as self-healing materials [221], aerodynamic designs [222], solar energy harvesting techniques [223], and catalysts [224]. This is because biological processes have undergone millions of years of evolutionary experimentation, resulting in the emergence of structures and functions with exceptional efficiency. Therefore, humans have often turned to nature's ingenuity to develop sustainable solutions to challenges facing society. Certain biomacromolecules, such as proteins, DNA, and enzymes, have the ability to initiate the formation of POFs and influence the shape and structure of these porous crystals using a process that mimics natural mineralization [225–227]. These biomimetic mineralization processes can take place under physiological conditions and lead to the creation of RF-NPs with high efficiency and specific morphologies (Fig. 21a).

RF-NPs are a thriving class of crystalline materials with exceptional properties, such as extremely customizable and expansive porosity (with up to 90% free volume), exceptional surface area, and a wide range of possible framework compositions [228–230]. These qualities set RF-NPs apart from traditional inorganic porous solids and make them a valuable and versatile option for host–guest interaction [231–235]. In 2019, Liang et al. [236] synthesized and characterized the first class of bio-composites based on hydrogen-bonded organic frameworks as shown in Fig. 21b. Throughout their investigation, the authors referred to this biocompatible HOF as BioHOF-1 and the enzymes encapsulated with this material as enzyme@BioHOF-1. These composites exhibited remarkable stability in a variety of conditions, including water with a pH range of 5 to 10, polar organic solvents, and phosphate buffers. The composites also had relatively large pore dimensions (with a limiting pore diameter of around 6.4 Å), surpassing those of ZIF-based materials of sodalite topology (about 3.4 Å) [237, 238]. Interestingly, the HOF coating was found to provide protection to encapsulated enzymes such as fluorescein-tagged catalase (FCAT) and fluorescein-tagged alcohol oxidase against proteolytic enzymes (such as trypsin), chaotropic agents (such as urea), and high temperatures.

The shape and size of particles, as well as the openings of pores in hosts where the guest biomacromolecules are encapsulated, can have an impact on how efficiently the substrate enters and the product is transported, thus affecting the targeted applications. The Ouyang research group [241] came up with a method of employing a peptide-driven de novo assembly technique to tailor the structure of the host metal–organic framework MOF from its conventional three-dimensional polyhedral form to a flat sheet-like configuration. Through their experiments, they found that the enhanced bioactivity of the two-dimensional sheet-based MOFs can be attributed to the reduced path length and expanded pore structures. This led to an acceleration of the substrate's diffusion, as demonstrated in Fig. 21c. Furthermore, the research findings have shown that the use of newly developed MOF architectures exhibiting a distinctive pivot-rod-shaped morphology in a two-dimensional configuration as a means of entrapping biomacromolecules has resulted in increased activity of MOF biohybrids compared to other similar biohybrids. The unique properties of these systems facilitate intercommunication among the biomolecules, which acts as a shield to protect the biomacromolecules and biominerals hosted within, enabling these architectures to effectively execute their biological functions even within challenging environmental conditions without unfolding.

**Fig. 22:**
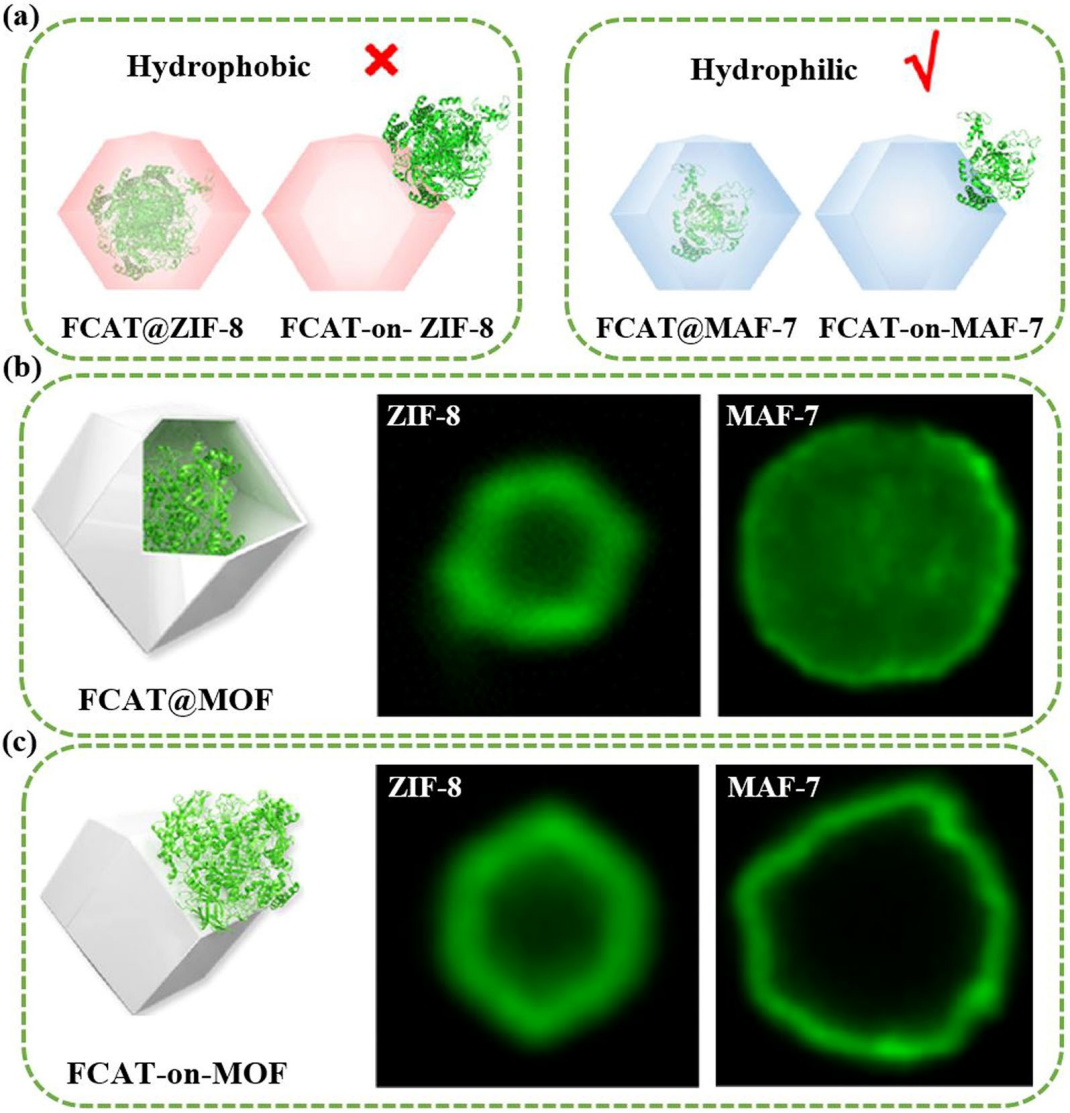
The role of hydrophilic and hydrophobic surfaces in the guest integration mode. **a** Illustration depicting diverse FCAT/MOF composites created by confining enzyme molecules via biomimetic mineralization or surface adsorption onto hydrophilic or hydrophobic Frameworks. Images obtained via confocal laser scanning microscopy exhibiting the fluorescent signals of **b** FCAT@MOF and **c** FCAT-on-MOF. Reproduced with permission from Ref. [247]. Copyright 2019, American Chemical Society

Exploring the fascinating world of host–guest interactions, researchers have dedicated significant efforts toward unraveling the complex mechanisms behind how adsorption on surfaces can modify the structure and activity of these fundamental biomolecules [242–245]. The process of adsorption can be quite complex, but it is commonly understood that biomolecules have a strong attraction to hydrophobic surfaces. Unfortunately, this can lead to conformational changes in the biomolecules, which can ultimately result in a loss of activity [246]. Recent research by the Doonan team [247] has shown that certain enzymes, including catalase, lose their activity when they are encapsulated within ZIF-8 crystals. As a result, the team sought to investigate whether ZIF materials with higher hydrophilicity could be better suited for enzymes, enabling them to retain their catalytic activity. Specifically, the team examined three ZIFs-based frameworks, featuring a well-defined sodalite topology, renowned for their structural integrity and distinct framework characteristics, each with similar structural metrics but chemically distinct ligands that significantly alter the hydrophobic/hydrophilic nature of unique structural frameworks exhibited by each individual ZIF (Fig. 22). Water adsorption isotherms were used to confirm that MAF-7 and ZIF-90 are much more hydrophilic than ZIF-8. The results of their experiments showed that MAF-7 exhibited a significant enhancement in the activity of both enzymes whereas within ZIF-8, the enzymes exhibited negligible activity in the case of urease and no activity at all for catalase.

### Surface Engineering via External Functionalization

**Fig. 23:**
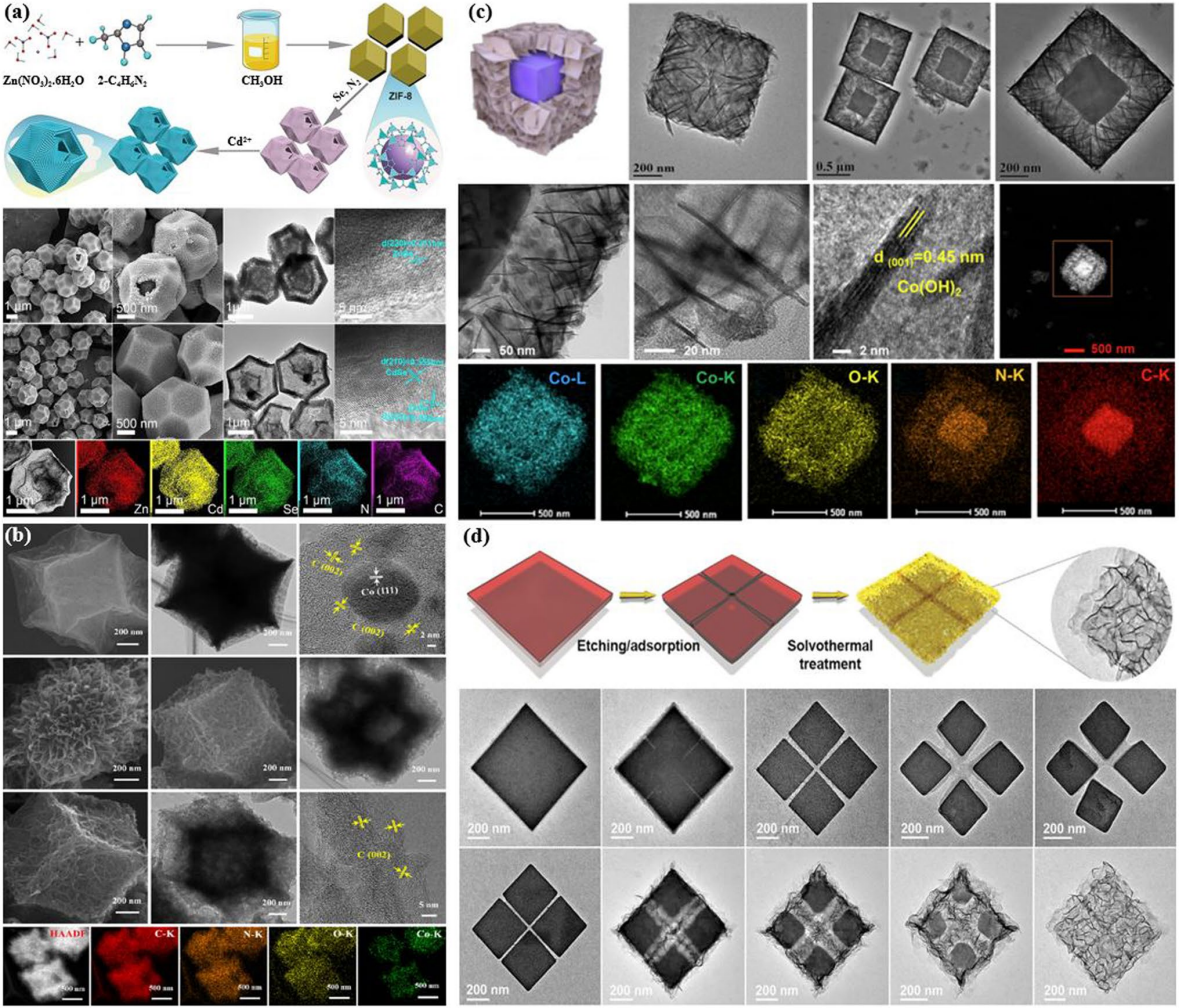
External surface functionalization. **a** Synthesis process of ZnSe-CdSe@NC frame-in-cage particles, which represents an instance of ZIF external surface functionalization, is presented through a schematic illustration. The SEM, TEM/EDS, and HR-TEM images of ZnSe@NC frame-in-cage particles are also provided (reproduced with permission from Ref. [253], Copyright 2022, Royal Society of Chemistry). **b** ZIF-67-derived particles generated through an external surface functionalization process using the direct core–shell strategy on RF-NPs. Several imaging techniques, including SEM, TEM/EDS, and HR-TEM, were utilized to visualize the resulting particles (reproduced with permission from Ref. [254]. Copyright 2020, Elsevier Inc.). **c** Morphological characterization of ZIF-67 functionalized by Co(OH)_2_ shell (reproduced with permission from Ref. [255]. Copyright 2020, Elsevier Inc.). **d** Schematic illustration and TEM images of single ZIF-67 NPs obtained through etching and hydrolysis processes with varied FeCl2 concentrations and duration. The resulting product was analyzed using TEM imaging, which revealed Single ZIF-67 NP at varying concentrations of FeCl2.4H2O/ethanol (ranging from 0 to 1.6 mg mL^−1^) (top), and with varying durations of 0, 3, 6, 9, and 15 min (bottom). Reproduced with permission from Ref. [256]. Copyright 2020, John Wiley & Sons Ltd

It is essential to modify the external surface of RF-NPs to enable their effective use in various applications. The surface properties of nanoparticles dictate their behavior and interactions with other nanoparticles and their environment. Hence, altering the surface characteristics is crucial to achieve desirable properties and functionalities for the nanoparticles [248]. Recently, there has been a notable emphasis on material chemistry research aimed at functionalizing the external surface of RF-NPs nanoparticles. This area of study has garnered significant attention as it is critical to attaining precise control over the properties and functionalities of nanoparticles [249–252]. As proof-of-concept, Han et al. [253] devised a method to create hybrid structures of dodecahedral shape called frame-in-cage (FC) structures composed of heterojunctions of ZnSe-CdSe encapsulated within a carbon matrix doped with nitrogen (Fig. 23a). The procedure encompassed the concurrent selenization and decomposition of a zinc-based zeolitic imidazolate framework, followed by a subsequent cation exchange utilizing ascorbic acid. Completely rephrase the provided sentence while ensuring that there is no similarity with the original sentence. By externally modifying the surface by exchanging cations, the hybrid structure could be converted from ZnSe@NC FC to ZnSe-CdSe@NC FC. The ZnSe-CdSe@NC FC catalyst had a unique frame-in-cage structure that provided efficient mass/charge transfer, active sites, and effective utilization of incident light, due to the ZnSe-CdSe heterojunction. The nitrogen-doped carbon also had strong light-to-heat conversion abilities. A remarkable synergy, harnessed as the photothermal effect, is created, further augmenting the overall efficacy. As a result of these features, the optimized composition of ZnSe-CdSe@NC FC exhibited superior activity and high stability during photo-thermocatalytic CO_2_ reduction, generating a higher CO production rate of 31.62 μmol h^−1^ g^−1^, which is much higher than what was achieved with single photocatalysis or thermocatalysis alone. According to Zhang et al. [254], their research group developed a highly efficient electrocatalyst made of nitrogen doped porous carbon using a sacrificial template in the form of a MOF-derived GC@COF core–shell heterostructure (Fig. 23b). This carbon heterostructure has excellent conductivity, a hierarchical porosity with both micropores and mesopores, and abundant N doping, which gives it a high activity for ORR in an alkaline solution. The resulting GC@COF-NC heterostructure demonstrates exceptional onset and half-wave potentials, a direct four-electron pathway, and good long-term stability. This innovative synthetic approach is anticipated to pave the way for the creation of other COF-derived graphitic carbon heterostructures doped with heteroatoms that have significant potential for electrocatalytic applications. On other hand, Qin et al. [255], reported the process of fabricating a new core–shell nanostructure, namely ZIF-67@Co(OH)_2_ (refer to Fig. 23c). This involved a series of steps starting with a high-temperature etching process, followed by refluxing and etching in a mixed ethanol/water solution. The outcome of this process was a loose Co(OH)_2_ shell, which offered a substantial number of active cobalt sites and improved contact for the oxygen evolution reaction (OER), in comparison to a complete hollow Co(OH)_2_ nanocube. The solid ZIF-67 core present within the ZIF-67@Co(OH)_2_ structure demonstrated favorable characteristics for charge transfer and provided a stable framework for the catalyst. The combination of the Co(OH)_2_ shell and ZIF-67 core exhibited a synergistic effect, resulting in optimized electrocatalysis for OER, particularly when subjected to appropriate etching regulation. Excitingly, Lou research team [256] applied the external functionalization route as an effective method for creating different hollow nanoparticles with exceptional electrocatalytic properties. The researchers utilized a multi-step approach involving the use of a specially designed zeolitic imidazolate framework as a starting material. The ZIF was first subjected to a series of chemical treatments which resulted in the deposition of various metal atoms throughout the structure (Fig. 23d). Following this step, the modified ZIF was subjected to a solvothermal reaction which transformed the structure into hollow nanoparticles composed of ultrathin nanosheets. The resulting nanoparticles were found to possess superior electrocatalytic activity for OER, and a crucial process for energy storage and conversion. Furthermore, the nanoparticles exhibit excellent stability even when subjected to high current densities for extended periods. Using external surface functionalization, this study in turn, provides exciting new avenues for the design and synthesis of highly efficient electrocatalytic materials for energy storage and conversion applications. Additionally, the functionalization of HOF materials has long been a challenge, but recent advancements offer promising strategies for improving HOF stability and expanding their applications. One efficient approach involves integrating functional species into the HOF structure by constructing an ionic framework. Recently, Liu’s research group [257] reported an efficient method for incorporating functional species into the structure of HOF architecture by constructing an anionic framework. They have successfully synthesized HOFs, exemplified by use of PFC-33, which combines a porphyrin photosensitizer as a porous backbone with a commercially available biocide as counterions within the structure. The resulting HOFs exhibit permanent channels and electrostatic interactions between the framework and the counterions, enabling ion-responsive release of the biocide in various physiological environments. This unique characteristic showcases synergistic photodynamic and chemical antimicrobial efficiency. Moreover, the unbonded carboxyl groups on the surface of the HOFs allow for manipulation of the interfacial interaction between PFC-33 and the polymer matrix during membrane fabrication. As a result, a poly-HOF membrane with exceptional stability, desired flexibility, and excellent permeability is obtained. The membrane demonstrates remarkable bacterial inhibition specifically against Escherichia coli. This research not only paves the way for the functionalization of HOF materials but also highlights their immense potential for a wide range of applications with remarkable stability.

## Are RF-NPs Safe? A Toxicity Evaluation

**Fig. 24:**
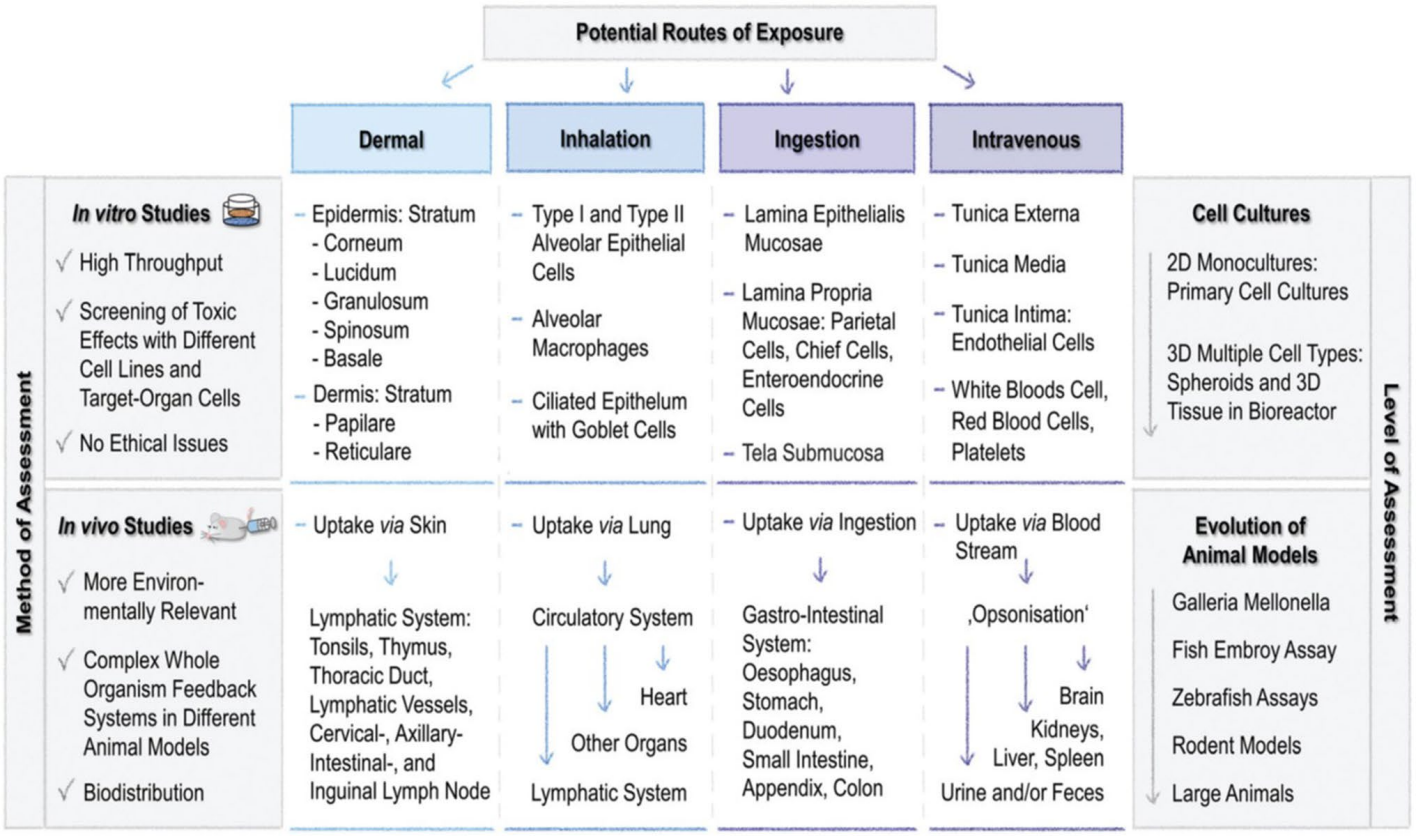
Overview of RF-NPs exposure routes and corresponding toxicity assessment requirements. Reproduced with permission from Ref. [270]. Copyright 2022, Royal Society of Chemistry

As scientists working on developing the next generation of nanoscale reticular frameworks, and considering that these tiny structures have the potential to revolutionize biomedical applications by precisely targeting and delivering drugs to specific cells in the body, as with any new technology, safety is a top concern. Assessing the safety of nanoparticles and comparing results from various studies can be challenging due to the intricate interactions between different processes that lead to toxicity and negative effects. As a result, it is difficult to provide a definitive rating of nanosafety and to make direct comparisons between data presented in the literature. To ensure that toxicity results obtained from nanomaterial studies are reliable, it is crucial to conduct a comprehensive material characterization. Without proper characterization, it is difficult to attribute the observed effects to specific properties of the nanoparticle [258–260]. To achieve this, it is essential to thoroughly investigate the physicochemical characteristics of a nanoparticle suspension, including factors such as chemical composition, size, shape, surface properties, charge, coating, dispersion, agglomeration, aggregation, concentration, and matrix [261, 262]. Figure 24 demonstrates that to accurately evaluate the toxicity of RF-NPs for their intended application, a diverse range of cells and assessment techniques must be thoroughly examined. Upon examination of the current state of hazard assessment for RF-NPs nanoparticles, it is clear that this field is still in its infancy and transitioning from basic research to real-world applications [263–267]. While various toxicity assessments have been extensively studied, exposure assessments have often been overlooked. However, significant progress has been made in the past decade, as the utilization of cell lines in in vitro studies has become increasingly relevant to applications [245, 268, 269]. Similarly, in vivo models should also be refined to ensure that the chosen model is as relevant as possible for an accurate hazard assessment of reticular nanoparticles in the context of their intended application. For developing a roadmap for the evaluation of the toxicity of RF-NPs: firstly, it is crucial to identify the potential routes of exposure for the reticular nanoparticles. Once these have been established, physico-chemical characterization studies should be conducted, including evaluations of the chemical and colloidal stability of the nanoparticles and the formation of a biological corona in contact with simulated biological fluids relevant to each exposure route. Following this, in vitro cytotoxicity screening of both individual components and assembled nanoparticles should be performed. This will provide valuable information on the potential toxicity of RF-NPs and their components at the cellular level. After assessing the cytotoxicity of nanoparticles, in vivo ADME (Absorption, Distribution, Metabolism, and Excretion) studies should be conducted to evaluate how these nanoparticles interact with the body on a systemic level. Finally, in vivo toxicity studies should be performed to assess the overall toxicity of RF-NPs. This will involve evaluating survival rates, changes in behavior, and physiological parameters such as organ weight and biomarkers in blood and tissues. Histopathological examination of organs and tissues will also be crucial in determining any adverse effects caused by reticular nanoparticle exposure. In brief, a comprehensive evaluation of MOF nanoparticle hazard requires a series of stepwise approaches that encompass both in vitro and in vivo studies, and consider multiple parameters to ensure a thorough understanding of their potential toxicity.

## Applications Portfolio: RF-NPs-derived Frameworks as Multifunctional Materials

### RF-NPs for Biomedicine

**Fig. 25:**
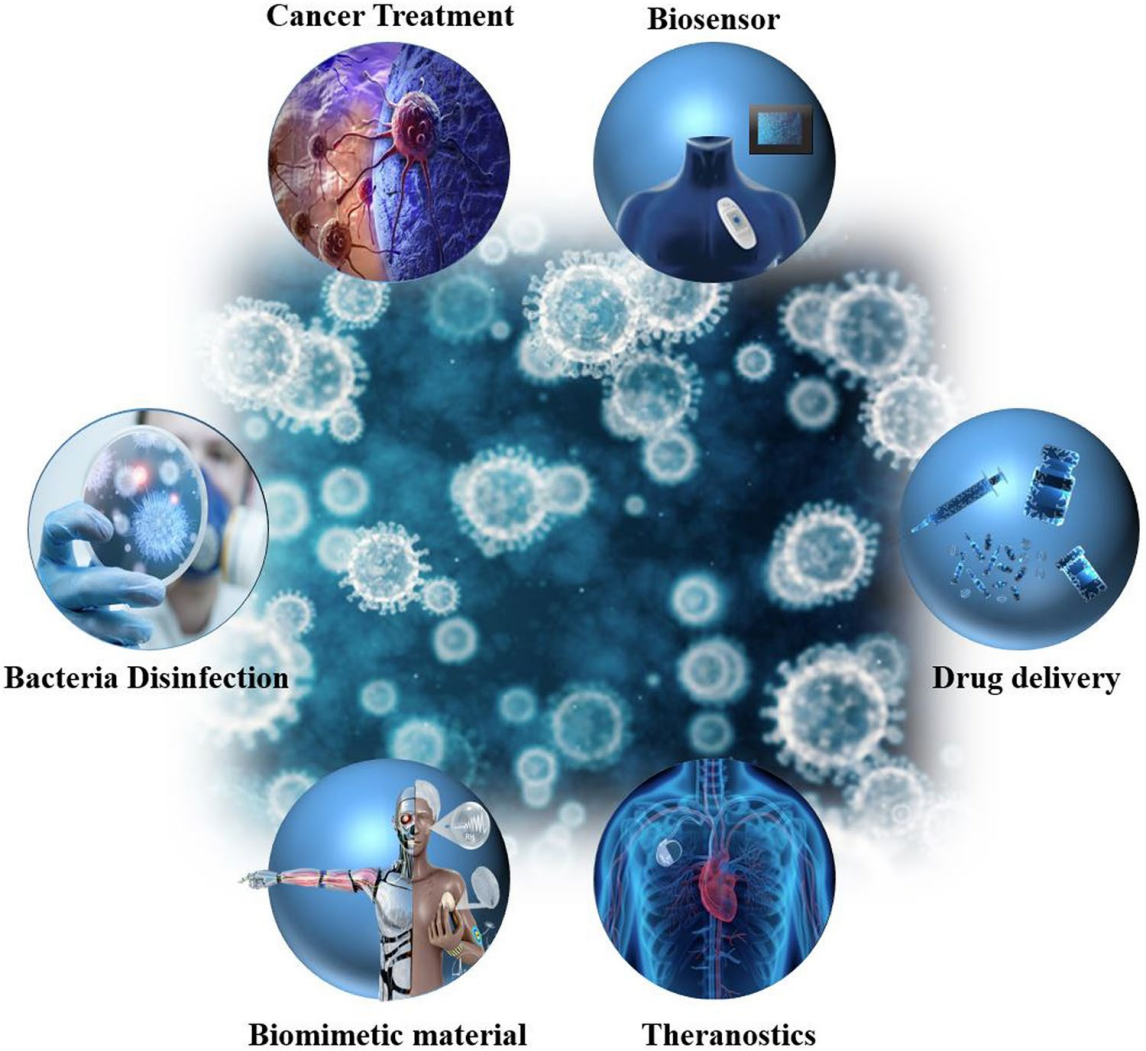
RF-NPs: the nanoparticles that could change the face of biomedicine. A diagrammatic representation of RF-NPs designed for various biomedical purposes

One of the primary uses of RF-NPs in bio-applications is to utilize their pore volume to contain an active molecule and transport it through considerations such as host–guest interactions, diffusion, and degradation [233, 271–274]. Essentially, the porous solid serves as a vehicle for delivering the active molecule to its intended destination [275–278]. Figure 25 illustrates the main developments in the use of RF-NPs for biomedicine applications. In this context, two essential steps are involved. Firstly, it is necessary to ensure that the porous solid possesses an adequate pore size and volume, allowing for optimal customization of the cargo to accommodate a specific biomolecule. Secondly, the cargo loading process entails encapsulating the drug molecule by immersing it in a solution, followed by a subsequent cleaning step. The outcome of the encapsulation process, specifically in terms of successful loading, becomes uncertain due to the interplay between the size of the molecule and its affinity toward the porous matrix. Overall, the impressive attributes of RF-NPs, characterized by their high surface area, abundant and large pores, biodegradability, adjustable pore structure, and easy surface modification, make them highly attractive for diverse different applications. In fact, the choice of excluding a particular composition for biomedicine applications can be influenced by multiple parameters. These include the specific application, the careful evaluation of risk and benefit trade-offs, the degradation kinetics, biodistribution patterns, accumulation tendencies in tissues and organs, as well as the body's ability to eliminate the material. Consequently, while various RF-NPs may find utility in such applications, their implementation would vary based on dose adjustments dictated by the aforementioned criteria (See the precedent section for toxicity evaluation of RF-NPs).

**Fig. 26:**
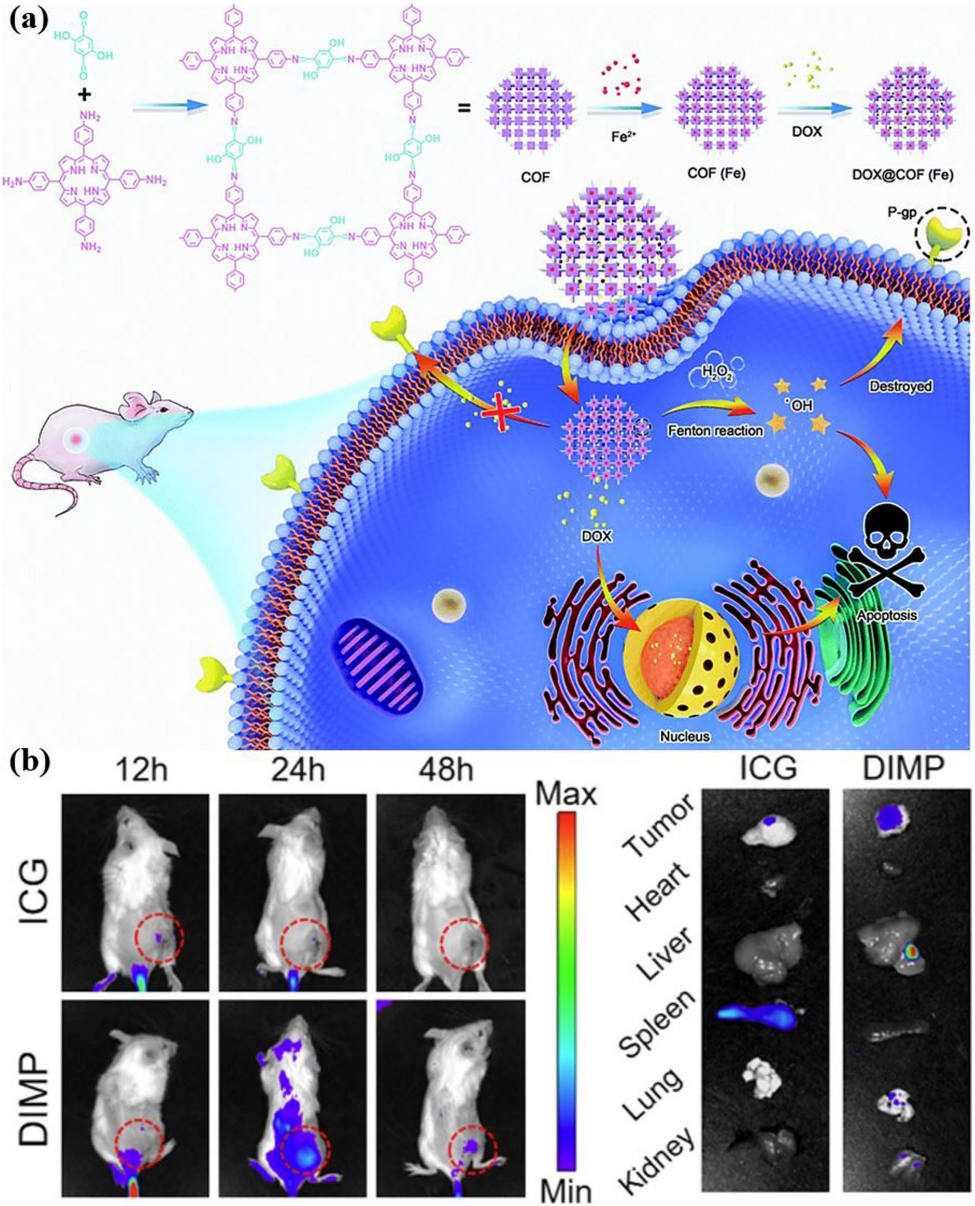
RF-NPs for cancer therapeutics. **a** Schematic illustration of the single-atom enzyme COF(Fe) synthetic procedures and its utilization in tumor multidrug resistance (reproduced with permission from Ref. [279]. Copyright 2021, Royal Society of Chemistry). **b** Fluorescence images captured at various time points to visualize the 4T1 tumor-bearing mice after injection of ICG and DIMP, where the tumors were indicated by red circles. Furthermore, in vivo fluorescence images of the tumors as well as various organs including the heart, liver, kidney, spleen, and lung were also presented. Reproduced with permission from Ref. [280]. Copyright 2021, Springer Nature

The hybrid nanomedicine shows promising results in vivo, effectively inhibiting tumor growth while minimizing systemic toxicity. The use of MOF and gold nanoparticles as building blocks, along with the triggered drug release and cascade catalytic reactions, makes this nanomedicine a potential candidate for future cancer therapies. On other hand, the research conducted by the Tang research group [279] focused on the development and synthesis of Fe-porphyrin COF nanoparticles [referred to as COF(Fe)] designed to address the challenge of multidrug resistance in tumors (as shown in Fig. 26a). The team successfully prepared porphyrin COF nanoparticles with excellent crystalline structure and uniform size distribution. Subsequently, they coordinated these nanoparticles with Fe^2+^ to obtain COF(Fe), which demonstrated both high catalytic activity and drug loading capacity. In their study, the researchers loaded the anticancer drug doxorubicin into COF(Fe) to create a drug delivery system referred to as DOX@COF(Fe). They observed a significant cellular internalization effect of DOX@COF(Fe), with the drug being released gradually in the acidic microenvironment found inside cells. Importantly, the Fe catalytic sites within COF(Fe) were found to convert H_2_O_2_ into OH, leading to the induction of oxidative stress and downregulation of P-gp expression. These effects proved instrumental in effectively overcoming tumor multidrug resistance, as demonstrated in both in vitro and in vivo experiments [279].

**Fig. 27:**
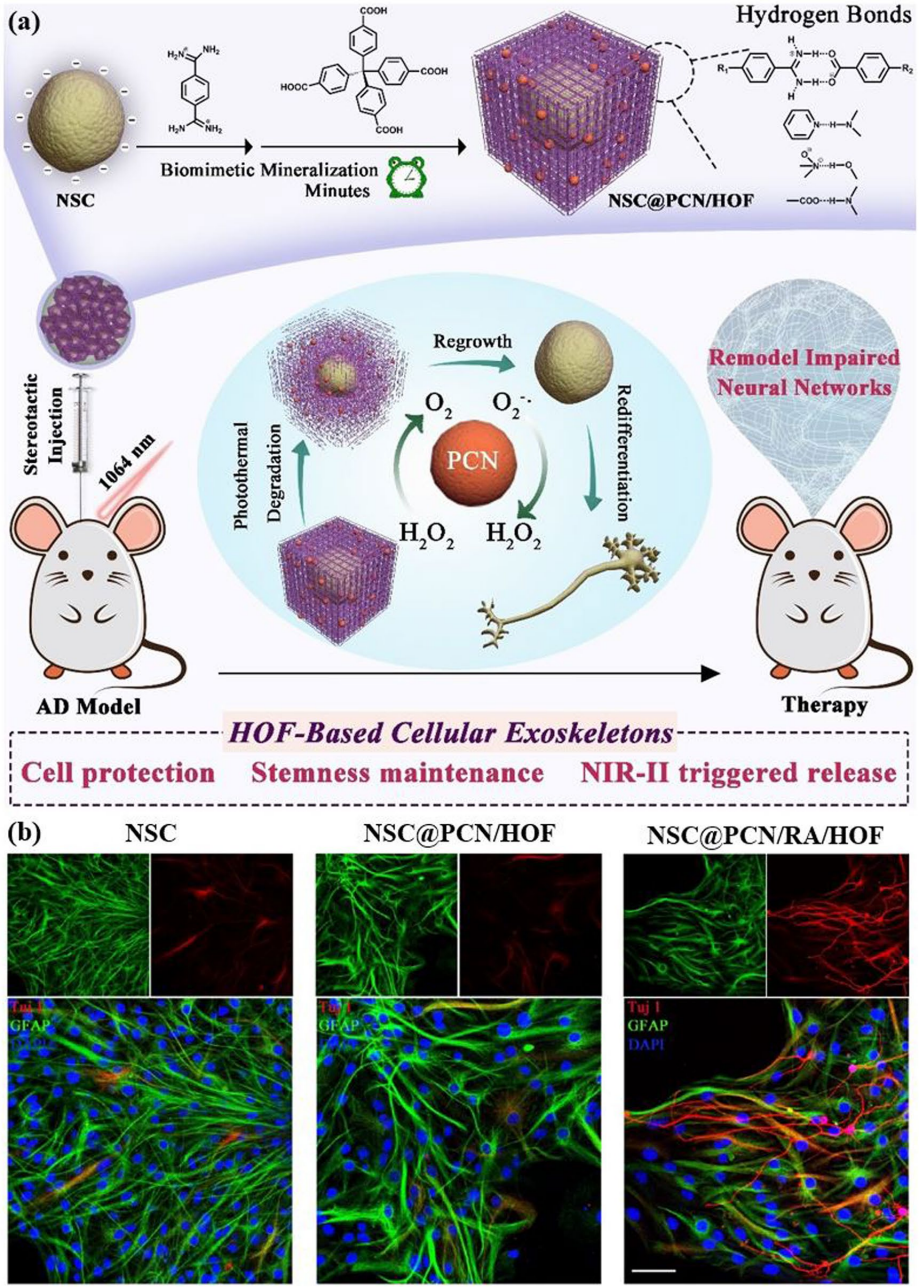
**a** Highly detailed visual representation depicting the process of HOF shell formation and subsequent elimination on individual neural stem cells, alongside an elucidation of the underlying mechanism responsible for the restoration of dysfunctional neural networks through remodeling. **b** Following the removal of HOF shells, neural stem cells (NSCs) underwent neural differentiation, and the subsequent characterization of NSC@PCN/HOF cells was conducted using specific markers such as GFAP and Tuj 1, which enabled the detection of glial cells and neurons. The scale bar in the image corresponds to 50 μm. Reproduced with permission from Ref. [281]. Copyright 2022, John Wiley & Sons Ltd

In addition, Zhang et al. [280], successfully developed a novel nanoparticle called DIMP, which exhibits a remarkable response to the acidic conditions within cells. This innovative polymeric metal organic framework nanoparticle demonstrates great potential for theranostic applications in breast carcinoma treatment. The unique feature of DIMP lies in its ability to co-deliver two essential agents: doxorubicin, a chemotherapy drug, and indocyanine green (ICG), a phototherapy agent. This combination enables a synergistic approach toward breast cancer therapy. DIMP possesses several advantageous characteristics, including its stable and suitable nanoscale size. Moreover, it demonstrates a specific response to the acidic microenvironment typically found in tumor cells. This acidity-responsive behavior allows DIMP to precisely target and deliver therapeutic agents exclusively to the tumor sites, enhancing the efficacy of treatment (Fig. 26b). Furthermore, DIMP triggers specific biological reactions that induce apoptosis, contributing to the eradication of cancer cells. The study conducted both in vivo and in vitro experiments to evaluate the performance of DIMP. The results demonstrated that DIMP exhibits excellent tumor accumulation properties, effectively concentrating within the tumor sites. Moreover, the nanoparticle exhibited a powerful effect in inducing immunogenic cell death, which holds significant promise in stimulating the immune system to mount an effective response against cancer cells. Moreover, Yu et al. [281] presented a novel approach for encapsulating neural stem cells (NSCs) using a biocompatible composite of hydrogen-bonded organic framework (HOF). The HOF-based shells offered distinct advantages, including safeguarding NSC stemness prior to transplantation and minimizing cytomembrane damage caused by syringe needle injection during cell transplantation (Fig. 27). Additionally, porous carbon nanosphere nanozymes (PCNs) were incorporated into the HOF shells during the mineralization process, providing them with enhanced features. These shells exhibited the following characteristics: 1) NIR-II photothermal effect and hierarchical hydrogen bonds for NIR-II triggered degradation of the HOF, 2) resistance to oxidative stress for encapsulated NSCs due to PCNs with SOD and CAT-like enzymatic activities, and 3) serving as a drug carrier with the ability to load RA, thereby promoting NSC differentiation into neurons. Compared to direct transplantation of naked NSCs, stereotactic transplantation of NSC@PCN/RA/HOF into the hippocampus of mice demonstrated improved NSC viability, enhanced neurogenesis, and alleviation of cognitive disorders in aged 3× Tg-AD mice. This study marks the first instance of utilizing HOFs as an artificial exoskeleton for cell transplantation, offering valuable insights into the design and synthesis of HOF-based multifunctional exoskeleton NSCs to ameliorate neurogenesis and cognitive behavioral symptoms associated with Alzheimer's disease.

More strikingly, HOF nanomaterials possess notable attributes of biocompatibility and minimal toxicity, which can be connected to their non-metallic characteristics. As a result, they emerge as exceptionally promising contenders for applications in photodynamic therapy. As a proof of concept, Liu’s research group [282] proposed an innovative approach to the creation of a highly stable HOF (PFC-1), utilizing a rational design strategy. PFC-1 exhibits exceptional chemical stability and boasts a remarkably high surface area. To address the challenge of thermal damage, the researchers have introduced a novel acid-assisted crystalline redemption method, which effectively repairs any structural deterioration in PFC-1. Notably, through the periodic integration of a photoactive pyrene component into the robust framework, PFC-1 demonstrates a remarkable ability to encapsulate Doxorubicin for synergistic chemo-photodynamic therapy. Impressively, this therapeutic approach showcases comparable efficacy to commercially available Doxo treatments while significantly reducing cytotoxicity. The groundbreaking work by Liu’s research team not only tackles the longstanding stability concerns associated with HOFs but also paves the way for the development of robust HOFs with tremendous potential for diverse applications in the future.

**Fig. 28:**
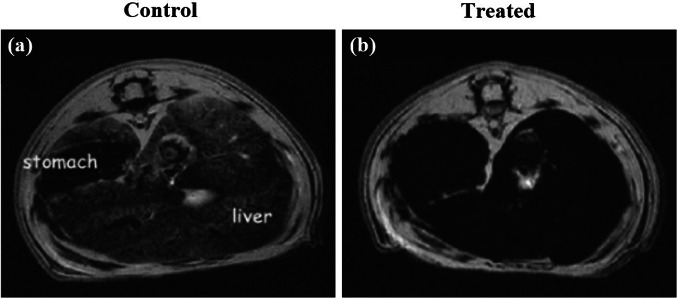
A comprehensive magnetic resonance imaging (MRI) study was conducted, precisely 30 min following the administration of two distinct solutions: **a** an isotonic solution serving as the control and **b** MIL-88A nanoparticles in female Wistar rats. The MRI scans were meticulously carried out using a 7 T horizontal-bore magnet operating at 300 MHz. Subsequently, the rats were humanely euthanized through an isoflurane overdose, ensuring the utmost care and ethical practices were upheld throughout the experimental procedure. Reproduced with permission from Ref. [266]. Copyright 2010, Springer Nature

The considerable specific surface area and extensive porosity of RF-NPs prove advantageous in enhancing the quantity of drug loading. However, “Theranostics” poses a significant challenge in drug therapy, involving the simultaneous tracking of drug delivery in the body using the inherent imaging properties of reticular frameworks and assessing the therapy's effectiveness [283–285]. To address this objective, researchers have undertaken a Postsynthetic modification of the surface of iron terephthalate MIL-101 nanoparticles by incorporating amino terephthalate groups [266]. Subsequently, they loaded an anticancer drug (12.8 wt%) and a fluorophore (5.6%-11.6%) onto the MOF to create a single nanoparticulate system capable of combining optical imaging capabilities with anticancer therapy. Theranostics holds great potential in drug therapy, and a promising avenue involves the utilization of biocompatible porous iron (III) carboxylates [266, 286]. These compounds exhibit exceptional drug-loading capacities, accommodating a wide range of drugs with diverse physicochemical characteristics, including hydrophobic, amphiphilic, and hydrophilic properties. Moreover, their relaxivities can be tailored for effective in vivo applications. By harnessing these features, non-toxic porous iron (III) carboxylates offer a compelling approach for theranostics, enabling the simultaneous delivery of drugs and non-invasive imaging for improved diagnostics and treatment monitoring. MRI measurements were conducted on female Wistar rats, specifically focusing on the liver and spleen, after injecting suspensions of MIL-88A and MIL-100 nanoparticles. The obtained results from both gradient echo and spin echo sequences unequivocally indicated that the treated liver and spleen exhibited a darker appearance compared to the normal organs. This distinct contrast was attributed to the preferential accumulation of the nano-MOFs in these specific reticuloendothelial system organs. Notably, Fig. 28 visually depict the observable dissimilarities in the liver and spleen between the control group and the group that received nanoparticle treatment. These findings present exciting prospects for enhancing the efficacy of anticancer and antiviral medications, as well as for creating customized formulations for pediatric use utilizing RF-NPs, representing a significant advancement in medical treatment.

**Fig. 29:**
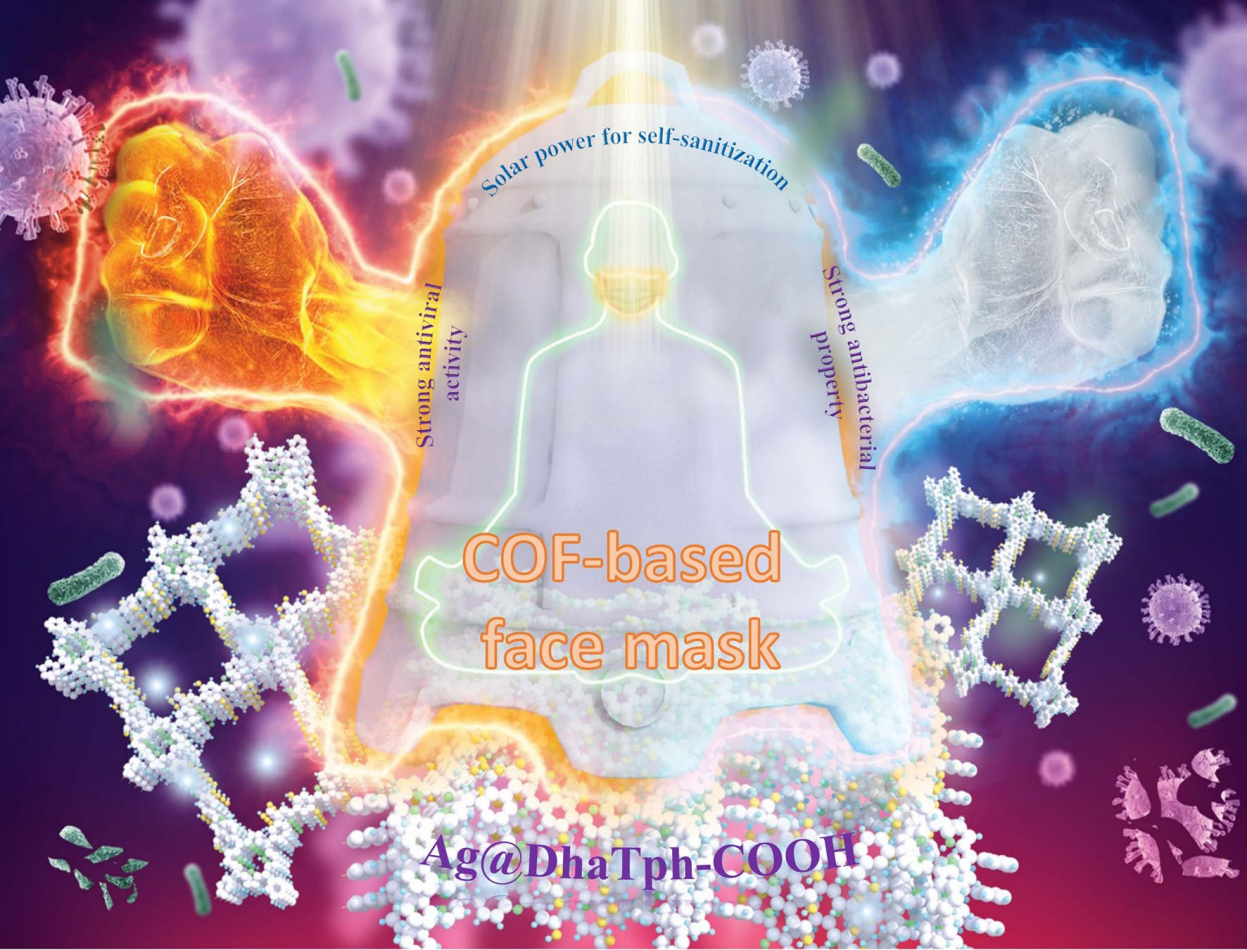
Utilizing Nano-silver-loaded COF as an effective self-sterilizing coating for bioprotective masks targeting bacteria and viruses. Reproduced with permission from Ref. [287]. Copyright 2022, Royal Society of Chemistry

Amidst the swirling chaos unleashed by the COVID-19 virus and the relentless onslaught of respiratory viruses, one humble hero stands firm, ready to defend us in the face of uncertainty: the mighty face mask. Far from a mere fashion accessory, these unassuming protective shields have emerged as the stalwart defenders of our well-being, serving as an essential line of defense during the pandemics. Conversely, the widespread adoption of disposable face masks presents formidable obstacles not only in terms of recycling and sterilization of used masks, but also in terms of exacerbating plastic pollution and resource depletion. Hence, there is a pressing need for the advancement of self-sanitizing reusable face masks, underlining the utmost importance of this development in addressing these challenges. As a proof of concept, Dong research team recently achieved a groundbreaking milestone by successfully creating a novel self-sanitizing reusable face mask (Fig. 29) [287]. Their innovative approach involved the fabrication of a quinoline carboxylic acid-linked and hydroxyl-enriched porphyrin-COF. This remarkable face mask exhibited exceptional biocidal efficacy against bacteria and viruses present in contaminated aerosols. Notably, the mask’s biocidal performance was convincingly demonstrated and its ability to harness solar power further enhanced its functionality. Moreover, the face mask showcased remarkable recyclability, making it a highly sustainable and environmentally friendly solution. This strategy encompasses far-reaching implications, transcending the boundaries of synthetic methodologies and unlocking unexplored avenues for COF applications. Moreover, it harbors the potential to revolutionize the very fabric of solar-powered reusable personal protective equipment manufacturing, presenting an invaluable solution amidst the arduous challenges posed by the pervasive worldwide spread of the COVID-19 virus and other respiratory ailments. Prepare to witness a paradigm shift in safeguarding our collective well-being.

### RF-NPs for Gas Valorization

**Fig. 30:**
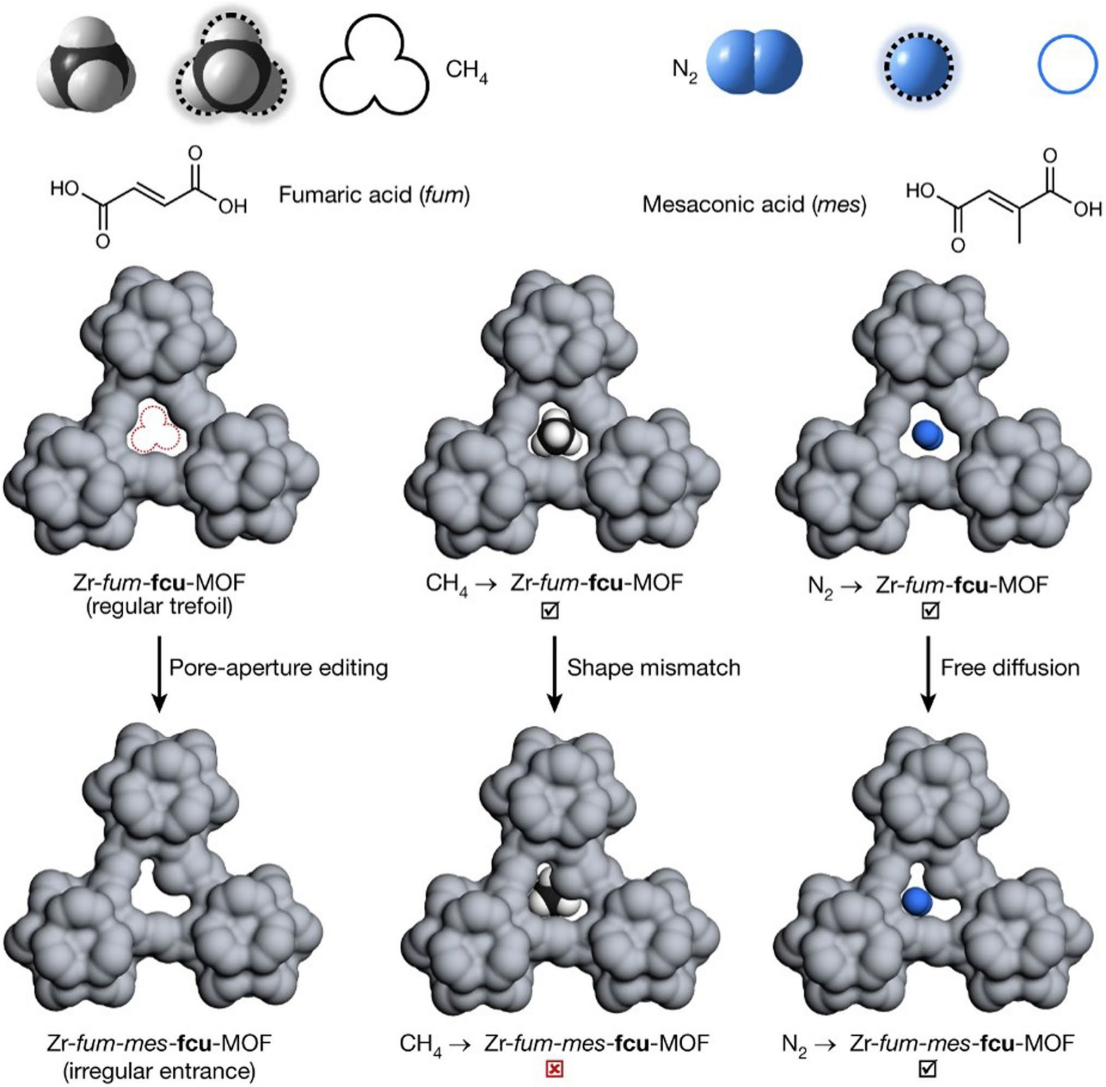
Visual depictions of pore-aperture editing and shape-mismatch induced separation for effective shape-based differentiation. Reproduced with permission from Ref. [151]. Copyright 2022, Springer Nature

The global energy supply relies on natural gas for at least 25% of its needs, and it is projected to surpass coal as the dominant energy source by approximately 2032 [288, 289]. However, this growth poses challenges for traditional methods of purifying natural gas. This is due to the presence of nitrogen (N_2_) and carbon dioxide (CO_2_) contaminants in natural gas reservoirs. In fact, around half of the world's natural gas reserves, referred to as sub-quality reservoirs, exceed the maximum allowable 4% N_2_ specification for pipeline transport. As a result, there is a need to explore energy-efficient and cost-effective technologies capable of effectively separating N_2_ from methane (CH_4_) [290]. Aiming at this challenge, Eddaoudi research group [151] recently developed a novel MOF membrane called Zr-fum67-mes33-fcu-MOF. This membrane utilizes a combination of fumarate (fum) and mesaconate (mes) linkers, and it possesses a pore aperture shape that is specifically designed to effectively remove nitrogen from natural gas (Fig. 30). By intentionally introducing asymmetry into the pore aperture, which is originally trefoil-shaped, the researchers were able to create a shape irregularity that hinders the transport of tetrahedral methane while allowing linear nitrogen molecules to permeate through the membrane. This unique feature results in a remarkable nitrogen/methane selectivity and nitrogen permeance, particularly at practical operating pressures of up to 50 bar. Consequently, the Zr-fum67-mes33-fcu-MOF membrane can effectively remove both carbon dioxide and nitrogen from natural gas. In addition to its exceptional performance, a techno-economic analysis conducted on these membranes demonstrates their potential to significantly reduce the cost of methane purification. Specifically, the membranes offer approximately a 66% cost reduction for nitrogen rejection and about a 73% cost reduction for simultaneous removal of carbon dioxide and nitrogen, when compared to traditional methods such as cryogenic distillation and amine-based carbon dioxide capture. In summary, the Eddaoudi research group mixed-linker MOF membrane, Zr-fum67-mes33-fcu-MOF, exhibits outstanding properties for nitrogen removal from natural gas, surpassing previous records in terms of nitrogen/methane selectivity and nitrogen permeance. Furthermore, these membranes have the potential to substantially reduce the costs associated with methane purification, making them a promising solution for the industry. In addition, COFs chelated with transition metals exhibit promising potential for utilization in hydrogen storage [203, 291, 292]. For instance, Mendoza-Cortes et al. [293] successfully constructed 30 novel COFs. These COFs were subjected to rigorous analysis to determine their hydrogen uptake capacities. The researchers employed advanced computational techniques, specifically quantum mechanics-based force fields and grand canonical Monte Carlo simulations, to accurately evaluate the H_2_ adsorption capabilities of these newly developed COFs. Significantly, this study stands out because, for the first time, it explored a wide range of adsorption pressures, spanning from 0 to 700 bar, at a fixed temperature of 298 K. By investigating this previously unexplored pressure range, the researchers aimed to understand the potential of these COFs in terms of H_2_ uptake and delivery, surpassing the limits of traditional bulk H_2_ storage. The results of the study unveiled that COFs based on cobalt, nickel, and iron exhibited exceptional H_2_ adsorption properties. Compared to bulk H_2_ under the same range of pressures, these metal-based COFs showcased considerably higher H_2_ uptake and delivery capabilities. This discovery highlights the promising potential of Co-, Ni-, and Fe-based COFs as viable alternatives for efficient H_2_ storage and transport systems. In conclusion, the utilization of reticular frameworks nanoparticles RF-NPs presents a compelling prospect for the conversion of gases, offering a multitude of advantages and opportunities to achieve efficient and sustainable gas valorization processes. RF-NPs, characterized by their porous structures and customized functionalities, possess remarkable adsorption and catalytic properties that facilitate the conversion of diverse gas feedstocks into valuable end products. An important benefit of RF-NPs lies in their ability to provide a large surface area and adjustable porosity, thereby enhancing their gas adsorption capabilities. This characteristic enables the efficient capture and separation of gases, enabling selective elimination of impurities and enabling the utilization of various gas sources. Furthermore, RF-NPs can be tailored to match specific gas compositions, thereby enabling targeted and efficient conversion of different feedstocks. Furthermore, RF-NPs exhibit exceptional catalytic properties, offering active sites for gas activation and conversion reactions. These nanoparticles can be modified with specific metal catalysts or organic groups to enhance catalytic performance and selectivity, thereby enabling improved gas conversion processes. Additionally, RF-NPs demonstrate remarkable stability and recyclability, making them highly attractive for large-scale gas valorization applications. Their robust nature allows for repeated use without significant degradation, reducing the need for frequent catalyst replacements and contributing to cost-effective and sustainable processes. Moreover, the modular nature of RF-NPs allows for convenient regeneration and modification, further augmenting their potential for commercial implementation. To summarize, RF-NPs hold tremendous promise in the realm of gas valorization, providing a versatile and efficient platform for sustainable gas conversion processes. Ongoing research and technological advancements in RF-NPs have the potential to revolutionize the field of gas valorization, contributing significantly to the transition toward a cleaner and more sustainable energy future.

### RF-NPs for Energy Storage

With their status as a burgeoning family of porous materials, RF-NPs have sparked significant research enthusiasm aimed at investigating the application of RF-NPs-related materials in electrochemical energy storage and conversion [294]. This subject has garnered considerable attention due to its potential for advancement and innovation [295–298]. Through a comprehensive examination of recent breakthroughs, we aim to propose prospective pathways for the advancement of functional materials associated with RF-NPs, with a particular focus on their application in the field of renewable energy [299–302].

**Fig. 31:**
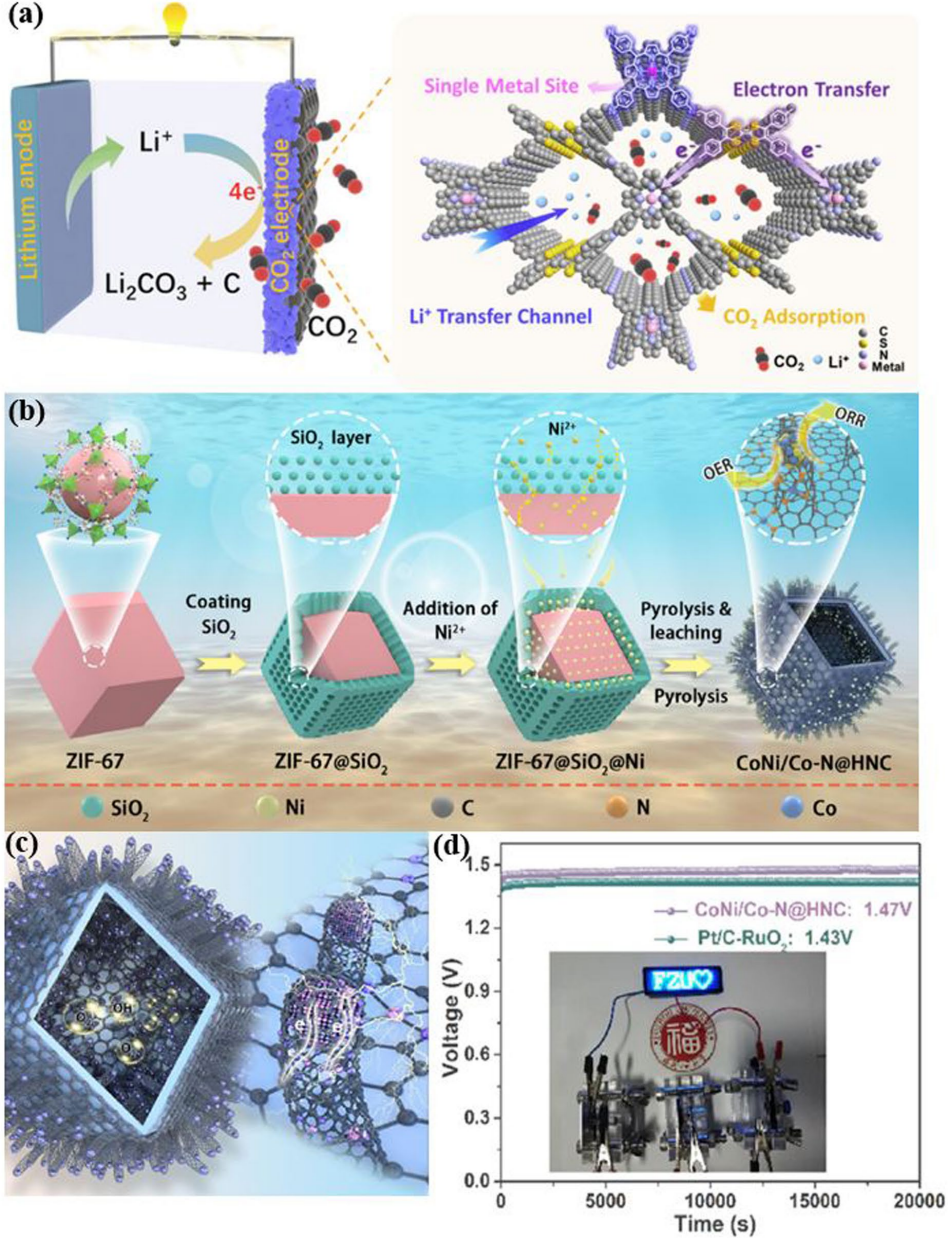
Functionalities of RF-NPs-derived materials for energy storage. **a** Schematic demonstrating the advantages of COF-Metal composites as catalysts for Li–CO_2_ battery cathodes (reproduced with permission from Ref. [307]. Copyright 2020, American Chemical Society). **b** Schematic depiction of the fabrication process for CoNi/Co–N@HNC composite material with its structural characteristics and **c** electronic effects in the conversion of O_2_ to OH−. **d** Electrochemical open circuit potential analysis of CoNi/Co–N@HNC and Pt/C-RuO_2_ catalysts, accompanied by an optical image of a LED panel illuminated by three series-connected rechargeable Zinc-Air batteries. Reproduced with permission from Ref. [308]. Copyright 2022, Elsevier Inc

Aprotic lithium-carbon dioxide (Li–CO_2_) batteries have emerged as a promising technology for both carbon dioxide (CO_2_) fixation and energy storage purposes. This innovative approach harnesses the electrochemical reactions of CO_2_ reduction and evolution, presenting a viable strategy to tackle CO_2_ emissions while offering a versatile energy storage solution. Consequently, Li–CO_2_ batteries have garnered significant global interest and have become a subject of extensive research in recent times [303, 304]. Zhou's group [305] presented a study on the utilization of Ru nanoparticles as a cathode catalyst in Li–CO_2_ batteries. The proposed Li–CO_2_ battery structure involves the transfer of Li^+^ ions and electrons to the cathode, where they react with CO_2_, leading to the formation of Li_2_CO_3_ on the surface of the porous carbon electrode. Upon charging, Li_2_CO_3_ decomposes back into Li^+^ ions and CO_2_. The evenly distributed Ru nanoparticles on the carbon electrode serve as highly active catalytic sites for the oxidation of Li_2_CO_3_ during the charging process. Experimental findings demonstrated that the incorporation of Ru nanoparticles notably decreased the charge overpotential and improved the cycling performance of the Li–CO_2_ battery. This preliminary study primarily focuses on the catalyst application in Li–CO_2_ batteries. Furthermore, it was observed that an irreversible reaction pathway, namely the self-decomposition of Li_2_CO_3_, often occurs during the charging of Li–CO_2_ batteries. The inclusion of Ru as a catalyst offers the possibility of circumventing this reaction and promoting the reversible interaction between Li_2_CO_3_ and carbon. This outcome aids in understanding why enhanced reversibility can be achieved in the presence of a Ru catalyst. However, based on the identified bottlenecks of the Li–CO_2_ battery and the advantages found in the presently documented cathode catalysts, as exemplified in Fig. 31a, it can be inferred that the design principles for cathode catalysts should encompass the following aspects: (i) demonstrating exceptional capability in capturing CO_2_, (ii) possessing uniformly distributed and well-defined catalytic sites for the reduction/evolution of CO_2_, (iii) establishing efficient pathways for the transfer of Li ions, (iv) ensuring effective electron transfer, and (v) exhibiting a distinct and well-structured configuration to enable accurate exploration of the underlying mechanisms [306].

Furthermore, in their pursuit of achieving precise control over oxygen-containing intermediates in oxygen catalysis, Tan et al. [308] have developed a meticulously designed electrocatalyst called CoNi/Co–N@HNC—a hierarchical 1D/3D hollow carbon nanopolyhedron (Fig. 31b). This unique catalyst exhibits a robust electron coupling between the encapsulated CoNi alloy and the Co-Nx sites within nitrogen-doped carbon nanotubes (N-CNTs), accomplished through a silicon protection-Ni infiltration strategy. The optimized CoNi/Co–N@HNC catalyst showcases exceptional performance in terms of reversible oxygen catalysis activity and stability, owing to the advantages offered by its open hollow carbon substrate, closely-integrated hierarchical 1D/3D structure, and the efficient charge regulation of Co-Nx sites facilitated by the CoNi alloy. Notably, the ORR/OER potential gap of CoNi/Co–N@HNC stands at a mere 0.73 V, outperforming the Pt/C-RuO_2_ catalyst, and the catalyst has demonstrated impressive longevity, operating continuously for over 350 h as an air cathode in practical applications. Through DFT calculations, they have discovered that the coupling with CoNi nanoclusters effectively lowers the d-band center of Co adsorption sites within CoN_4_, thereby optimizing the desorption capacity of oxygen-containing intermediates on Co sites. Consequently, this leads to a significant reduction in the kinetic energy barrier of the rate-determining step, thereby accelerating the overall kinetics of the ORR/OER reactions (Fig. 31b). This groundbreaking research provides valuable insights for the regulation and optimization of oxygen intermediates on transition metal-based oxygen electrocatalysts, with the aim of enhancing the performance of Zinc-Air batteries (Fig. 31c).

**Fig. 32:**
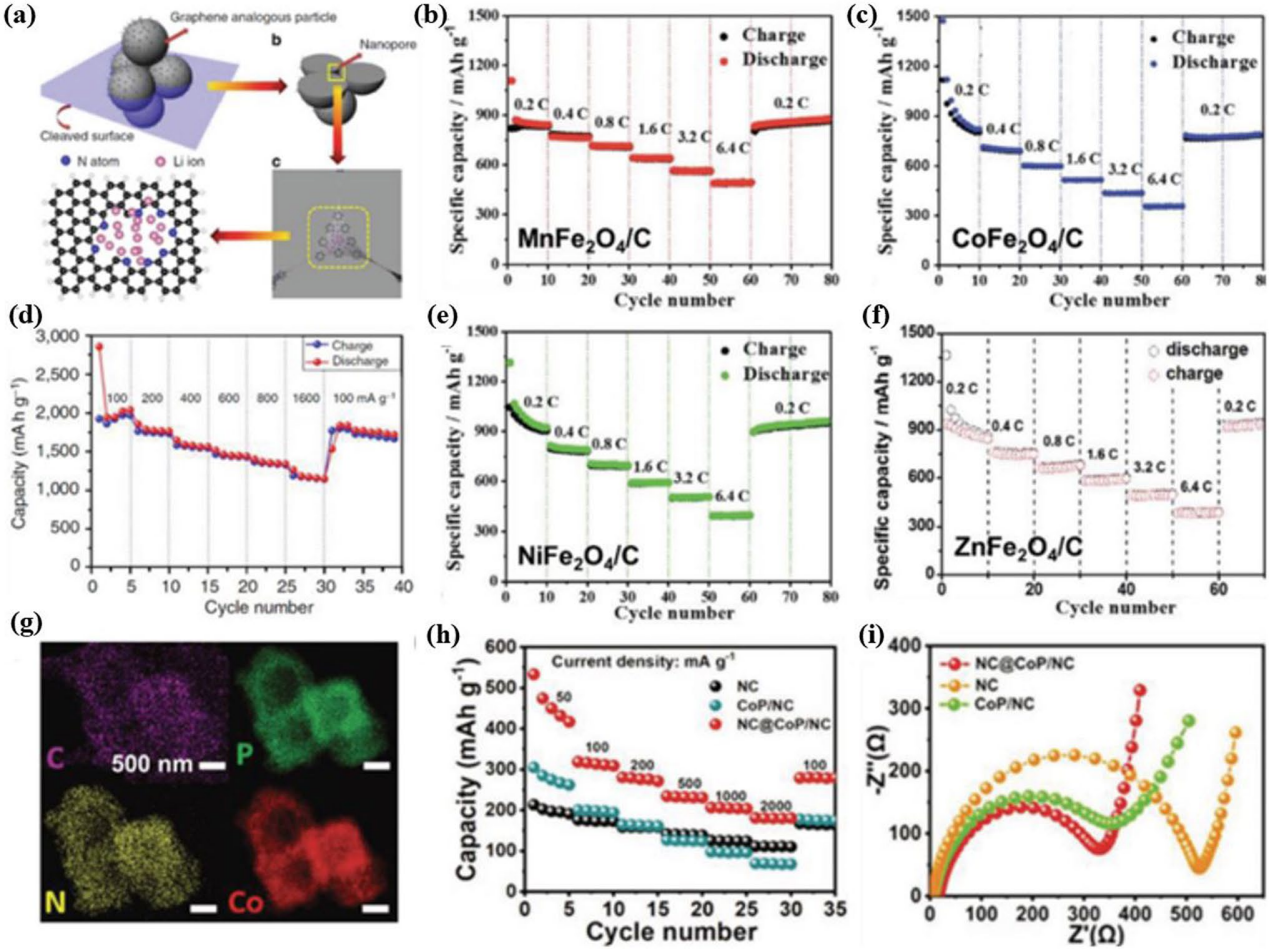
RF-NP-derived materials for advanced metal-ion battery performance. **a** Illustration of enhanced lithium storage in nitrogen-doped porous carbons for anodes in lithium-ion batteries (reproduced with permission from Ref. [315]. Copyright 2014, Springer Nature). **b** Rate performance of N–C-800 Electrode. **c**–**f** Bimetal oxides/carbon composites for LIB anodes: rate performance of MnFe2O4/C, NiFe2O4/C, CoFe2O4/C, and ZnFe2O4/C spindles. Reproduced with permission from Ref. [316]. Copyright 2017, Royal Society of Chemistry. **g** Elemental mapping of nitrogen-doped porous carbon confined CoP polyhedron (NC@CoP/NC). **h** Rate performance and **i** electrochemical impedance spectroscopy spectra of NC, CoP/NC, and NC@CoP/NC electrodes. Reproduced with permission from Ref. [323]. Copyright 2020, John Wiley & Sons Ltd

Lithium-ion batteries (LIBs) have become widely recognized as the primary choice for powering portable electronic devices, and they also hold tremendous potential for electric vehicles and hybrid electric vehicles [309–311]. LIBs are a type of rechargeable battery that operates by facilitating the migration of Li^+^ ions from the anode to the cathode during the discharge process and returning them during charging. This unique characteristic has contributed to their remarkable commercial success and established them as the dominant power source in various applications [312–314]. Chen’s research group has made a significant discovery in the field of materials science [315]. They have successfully developed particles that closely resemble graphene, but with the addition of nitrogen, resulting in nitrogen-doped graphene particle analogues (Fig. 32a). These particles have an impressive nitrogen content of up to 17.72 wt%. The synthesis process involves subjecting a nitrogen-containing zeolitic imidazolate framework to pyrolysis at a temperature of 800 °C. This process is carried out under a nitrogen atmosphere. By doing so, the nitrogen atoms are incorporated into the structure of the particles. These nitrogen-doped graphene analogues exhibit exceptional performance when used as anode materials in lithium-ion batteries. Specifically, they demonstrate a remarkable capacity retention of 2,132 mAh g^−1^ after undergoing 50 cycles at a current density of 100 mA g^−1^. Furthermore, even after 1,000 cycles at a higher current density of 5 A g^−1^, the particles retain a capacity of 785 mAh g^−1^. The outstanding performance of these particles can be attributed to the presence of nitrogen within the hexagonal lattice and edges of the graphene analogues. This nitrogen doping enhances the battery's overall energy storage capacity and improves its cycling stability, making it a promising advancement in the development of high-performance lithium-ion batteries. Moreover, Jiang et al. [316] developed a new technique for synthesizing spindle-shaped MFe_2_O_4_/C structures, where M represents Zn, Co, Ni, or Mn. The synthetic process involves creating mixed metal organic frameworks (Fe_2_M-MOFs) and subsequently subjecting them to calcination (Fig. 32b–f). The resulting MFe_2_O_4_/C spindles possess a fascinating architecture, comprising carbon-coated secondary nanoparticles of MFe_2_O_4_ interconnected by a carbon network. These structures hold great promise as active materials for a wide range of applications, offering enhanced performance.

On other hand, Potassium ion batteries (KIBs) are gaining attention as a promising energy storage system due to their cost-effectiveness and the abundance of potassium, making them an attractive alternative to lithium-based batteries [317, 318]. However, several obstacles hinder their practical application. These challenges include significant volume expansion and slow reaction kinetics caused by the relatively large size of potassium ions [319–321]. Consequently, the search for dependable anode materials that possess high electrical conductivity, an ample number of active sites, and structural stability is crucial for advancing KIB technology [322]. In this vein, Yi et al. [323] have published a significant study on the synthesis of unique anode materials for KIBs. In their work, they developed nitrogen-doped porous carbon confined CoP polyhedron architectures, embedded within ZIF-8@ZIF-67 frameworks, referred to as NC@CoP/NC composites (Fig. 32g–i). These composites exhibit remarkable performance in terms of both rate capability and cycling stability. The rate performance of the NC@CoP/NC anode materials is particularly impressive, as they can achieve a capacity of approximately 200 milliampere-hours per gram (mAh g^−1^) even at a high current density of 2000 milliamperes per gram (mA g^−1^). This indicates their suitability for fast-charging applications in KIBs. Moreover, the cycling stability of the anode is noteworthy, with a capacity retention of 93% maintained after 100 cycles at a lower current density of 100 mA g^−1^. This suggests that the anode materials can endure prolonged cycling without significant capacity degradation. To gain insights into the potassium ion storage mechanism of the NC@CoP/NC anode, the researchers employed both theoretical simulations and experimental characterization techniques. These investigations provided a systematic understanding of how the anode materials interact with potassium ions during the battery's operation. Such knowledge is valuable for further optimizing the design of anode materials and enhancing the overall performance of KIBs, which hold promise for the development of advanced KIB technologies, offering potential advancements in energy storage systems.

On the other hand, the utilization of RF-NPs offers a promising avenue for the exploration of novel electrochromic (EC) materials. The exceptional characteristics of these frameworks extend beyond their ease of use. They exhibit remarkable versatility, as they can be readily modified and possess a tunable structure, along with high porosity. These qualities grant the ability to fine-tune the electrochromic properties of RF-NPs at the atomic level, thereby enhancing their performance in EC applications. Additionally, the high porosity facilitates efficient electron transfer, leading to improved electrochromic responses. Specifically, HOFs enable the controlled assembly of well-established EC components through hydrogen bonding, eliminating the need for unnecessary elements such as polymerization or coordination agents. This approach not only ensures an atom-economical design of EC materials but also simplifies the overall process. Moreover, HOFs possess distinctive attributes, including solution processability and effortless regeneration, which greatly facilitate the fabrication process and allow for repeated utilization. For instance, Feng et al. [324] unveiled the remarkable capabilities of a robust and highly porous HOF named PFC-1. Employing the electrodeposition method, the researchers successfully deposited a thin film of PFC-1 onto transparent fluorine-doped tin oxide (FTO) glass, thus creating an electrochromic smart window. The resulting film exhibited a uniform surface with dense features and exceptional crystallinity, demonstrating its suitability for application on various substrates alongside different HOF materials. The electrochromic performance of the PFC-1 film on FTO glass was reversible, showcasing a captivating transformation from a vibrant yellow hue to a captivating blue-violet shade while reducing light transmittance from 75 to 25%. This impressive outcome can be attributed to the film's porous crystalline structure, nanoparticles with nanoscale dimensions, and excellent adherence to the substrate, all of which facilitated a rapid exchange of ions at the interface and prolonged performance durability. Additionally, the presence of unbound carboxylic acids in structural defects and on particle surfaces allowed for further modification of the film using iron ions, thereby enabling the achievement of multistate electrochromism. This finding serves as a testament to the unique tunability of HOF materials, opening exciting possibilities for tailored color displays and transformative applications that necessitate exceptional performance, extended cycle life, effortless processability, and recyclability.

Overall, the remarkable promise of RF-NPs has emerged in the realm of energy storage and conversion, primarily owing to their ability to undergo controlled transformation into carbon-based materials or diverse metal species. These resultant materials frequently demonstrate exceptional electrochemical performance, positioning them as highly desirable electrode materials for energy storage and conversion applications [325–328]. In addition, the utilization of POFs as precursor materials offers a wide range of possibilities for generating RF-NPs with different pore diameters, shapes, and compositions. This approach facilitates the fabrication of diverse nanostructures with varied microstructures and components. By incorporating different types of POFs, the synthesis of RF-NPs can significantly enrich the diversity of POFs derivatives. Moreover, the synergistic effects arising from the integration of multiple components hold the potential to enhance the electrochemical performance in energy storage and conversion systems. These include various secondary batteries, electrocatalysis of ORR and OER, solar cells, and other related fields.

## Setting the Stage for Digitization

### Digital Tools at the Service of RF-NPs Diversity

**Fig. 33:**
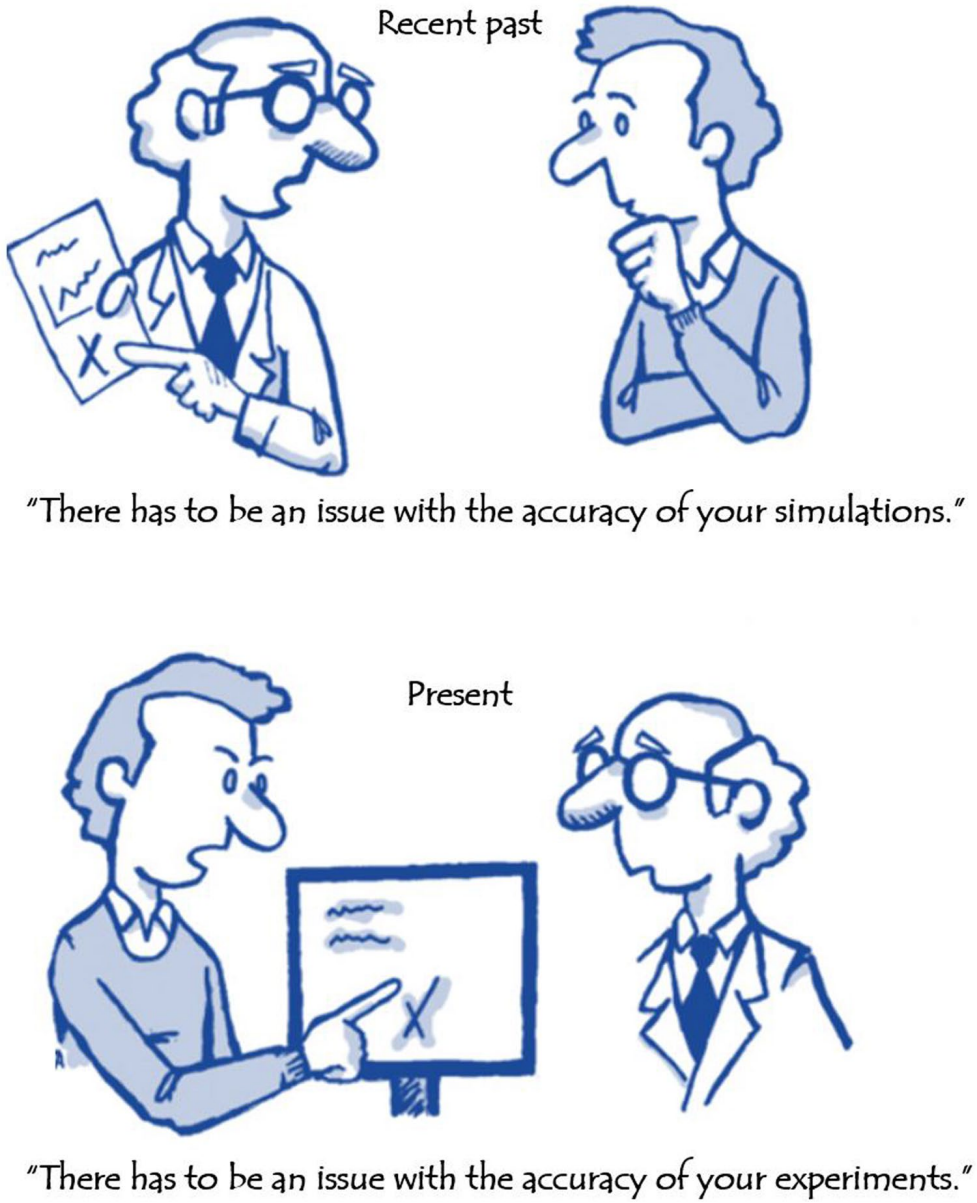
Digital simulations paradigm. From the confirmation of experimental results to the generation of new innovations

It was a pivotal moment where everything hung in the balance. Digital simulations became a method to generate new innovations, and even for the experimental studies, the episode will not be brought to close until a theoretical simulation validated the empirical data. In this stage, there have been numerous instances where theory has been fundamental in comprehending experimental outcomes and even predicting them (Fig. 33) [329].

RF-NPs in turn, the sheer multitude of organic linkers, the vast reticular structural space, along with the vast array of chemical alterations that can be employed to customize pore size and functionality, results in a daunting task that surpasses the boundaries of human capacity for envisioning and assembling such structures in an attainable period of time. Indeed, the realm of scientific knowledge is poised to expand and thrive exponentially as it incorporates and advances through the utilization of digital technologies. The combination of laboratory robotics and artificial intelligence (AI) presents an opportunity for conducting large-scale experiments that involve both the synthesis and characterization of structures within a feasible time frame [330]. By programming the robotic machinery and interfaces to automate the process, high-throughput experiments can be conducted [331–338]. Moreover, the digital tools available for this purpose will encompass data-mining capabilities, which will enable the digitization of text and collation of data. Furthermore, the use of machine learning algorithms will facilitate the making of data-driven decisions [339].

**Fig. 34:**
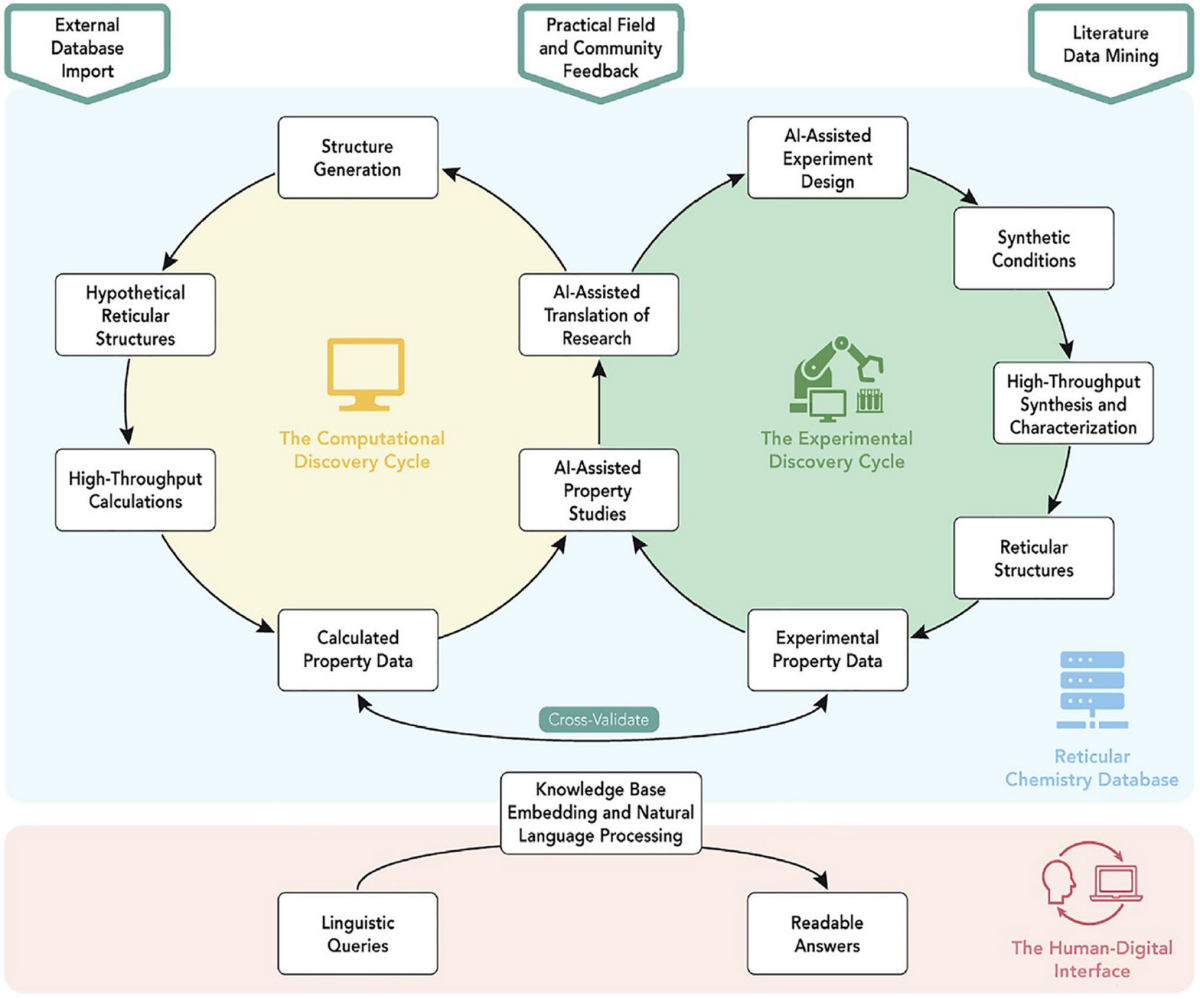
Transforming chemistry through digital reticulation: a comprehensive workflow. Reproduced with permission from Ref. [340]. Copyright© 2020, Cell Press

**Fig. 35:**
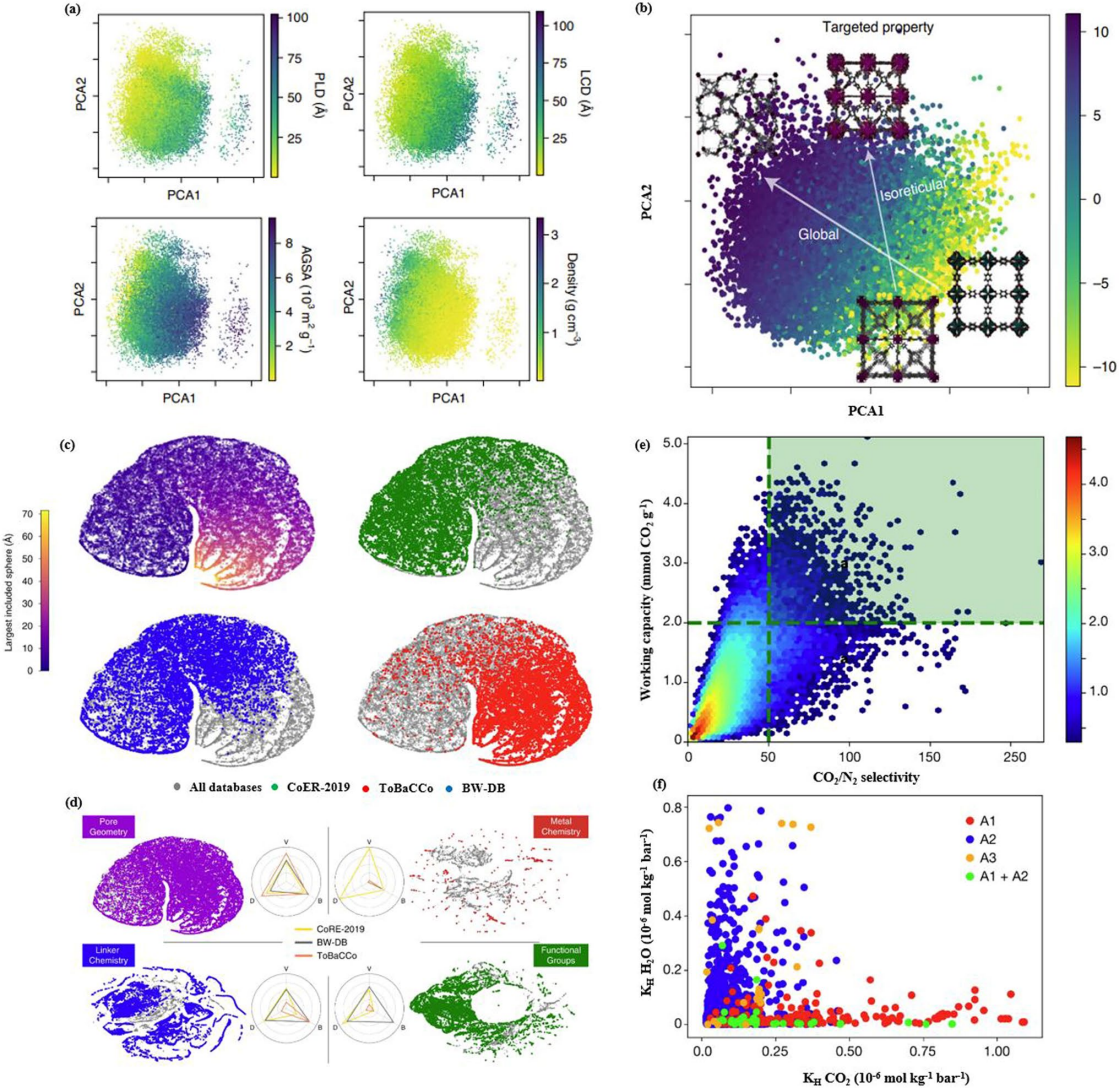
**a** Exploring the latent space of a jointly trained SmVAE through PCA analysis conditioned by MOF properties and exemplified sampling. **b** Comparing two optimized design processes for isoreticular frameworks targeting a specific application using MOF NU-110449 as a starting point. Reproduced with permission from Ref. [74]. Copyright 2021, Springer Nature. **c** Visualizing the pore geometry of MOFs through a 2D map generated by t-SNE Method for geometric descriptor space projection. **d** Exploring diversity metrics and domain-specific maps of MOF Structures: A 2D projection of pore geometry, metal and linker chemistry, as well as functional groups using t-SNE Method. Reproduced with permission from Ref. [341]. Copyright 2020, Springer Nature. **e** Computational screening of hypothetical MOFs for strong CO2 adsorption and selectivity under post-combustion capture conditions, **f** with further refinement and adsorbaphore identification of top-performing materials, including analysis of H_2_O affinity and adsorbaphore types. Reproduced with permission from Ref. [342]. Copyright 2019, Springer Nature

Figure 34 presents the general workflow of digital reticular chemistry, which revolves around four key components. Firstly, there is a database that facilitates the storage and transmission of information. Secondly, a computational discovery cycle utilizes the data generated by both humans and robots to make predictions. Thirdly, an experimental discovery cycle produces real-world experimental data through use of high-throughput methods based on AI suggestions. Lastly, a human-digital interface allows for the resolution of intellectual and practical issues in reticular chemistry. These pillars are integral to the process of digitizing reticular chemistry and will be explored further. For instance, Yao et al. [74] have developed an automated platform for the generative design of nano-porous materials using a supramolecular variational autoencoder (Fig. 35a, b). The platform was demonstrated on a class of MOF structures with the goal of separating carbon dioxide from natural gas or flue gas. The authors found that their model was able to accurately capture the structural features of MOFs and showed promising optimization capability when trained with multiple top adsorbent candidates. The MOFs discovered using this platform were found to be highly competitive against some of the best-performing MOFs and zeolites reported in literature. This new platform has the potential to accelerate the discovery of new materials for gas separation applications and improve the efficiency of separation processes. In addition, Kulik research group [341] has developed a new machine learning approach to quantitatively assess the similarities between MOFs and analyze their chemical diversity. Their diversity analysis technique aims to identify biases in MOF databases and to highlight situations where database composition can lead to erroneous conclusions (Fig. 35c, d). The proposed formalism in this study offers a simple and practical guideline to evaluate whether new MOF structures can provide new insights or merely represent a small variation of existing structures. By employing this methodology, researchers can make more informed decisions regarding which MOFs to prioritize for synthesis and characterization, ultimately leading to a more efficient and effective materials discovery process. The Kulik research group's findings provide valuable insights into how machine learning techniques can be utilized to optimize the discovery of new MOFs for various applications, including gas separation, catalysis, and energy storage. Smit and coworkers [342] have successfully employed data mining techniques on a screening library of more than 300,000 MOFs to uncover distinct classes of potent CO_2_-binding sites which confer CO_2_/N_2_ selectivity and exhibit remarkable effectiveness even in the presence of moist flue gases (Fig. 35e, f). Two water-resistant MOFs containing the most hydrophobic adsorbaphore were then synthesized and subjected to testing, revealing that their carbon-capture performance remained unimpaired by the existence of water, surpassing that of certain commercially available materials. Nonetheless, further exploration of these MOFs in an industrial context and comprehensive evaluation of the entire capture process, encompassing the intended CO_2_ storage destination, such as geologic storage or application as a carbon source for the chemical sector, are imperative to determine the optimal separation material. This investigation's outcomes furnish valuable insights into the potential of data mining and computational screening to discover novel MOFs with superior carbon-capture capabilities, enabling the development of more efficient and effective carbon capture technologies that could help mitigate climate change.

### “FAIR” Data: A Game-Changer Preparing the Research for Tomorrow

Data management, although essential, is not the ultimate destination; it's merely a necessary pit stop on the road to unlocking knowledge and driving innovation. By ensuring the proper management of data, it becomes possible to integrate and reuse this information, thereby facilitating further discovery and advancement within the broader community beyond the initial data publication process. The field of material science, and specifically reticular chemistry, generates an immense quantity of research data on daily basis. This data has the potential to be incredibly valuable in the twenty-first century, but only if it is properly characterized and made accessible. Without these crucial steps, the data may hold little significance. This can be achieved through the establishment of a data infrastructure that is “FAIR”: findable, accessible, interoperable, and reusable. Only with such a framework in place can we effectively derive meaningful insights from the data and drive innovation in these fields.

**Fig. 36:**
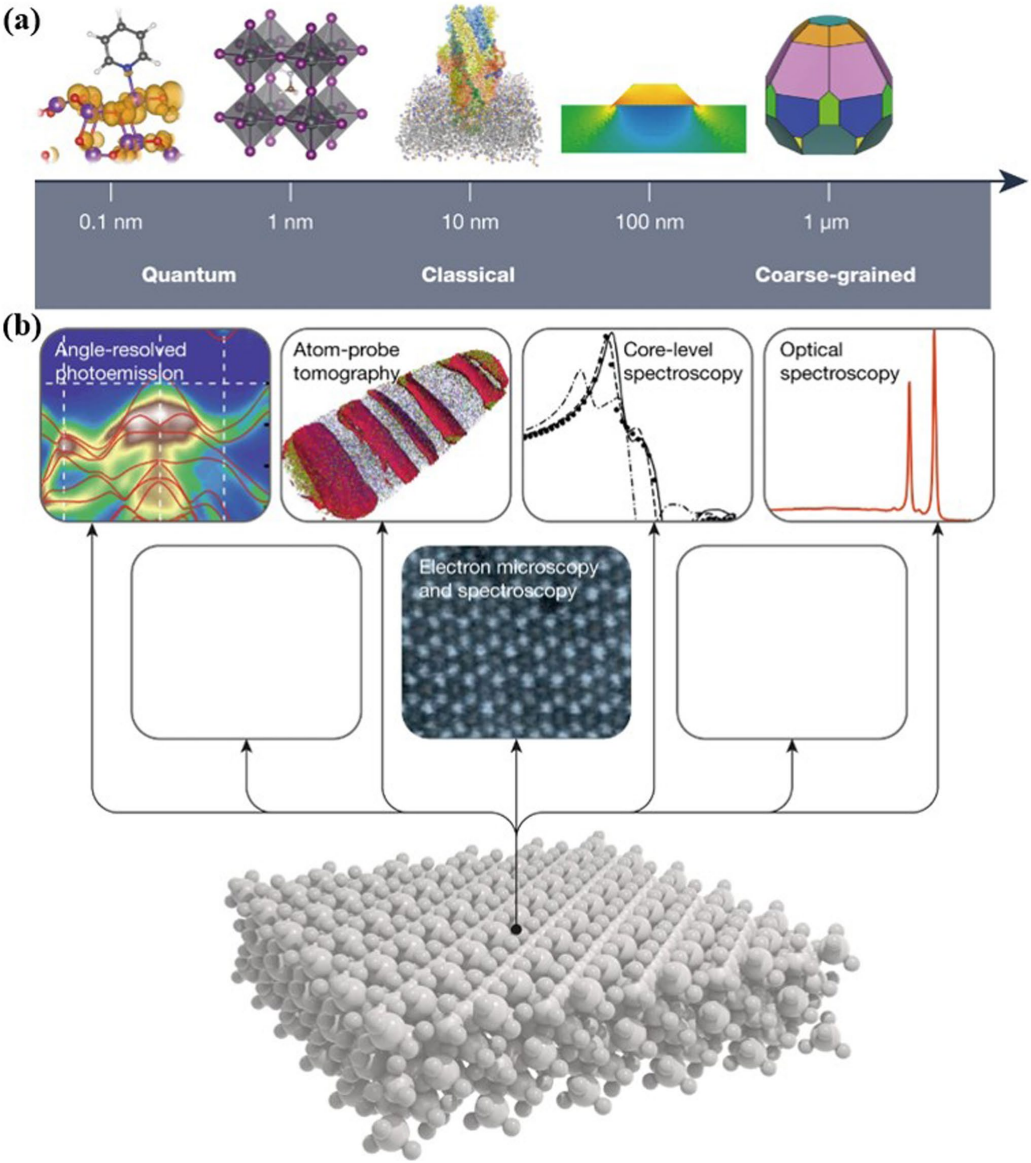
**a** Simulation of materials poses a significant challenge that spans across multiple length scales, from sub-angstrom dimensions to micrometers and beyond. The complexity of this challenge lies in the need to accurately model and simulate the behavior of materials at each of these scales, which requires distinct computational techniques and approaches. Additionally, there is a need to seamlessly integrate these techniques to ensure a consistent and accurate representation of the material behavior across the entire range of length scales. **b** Identifying key experimental techniques and developing digital FAIR data-management workflows for material characterization: Initial focus on five techniques. Reproduced with permission from Ref. [343]. Copyright 2022, Springer Nature

The development of a FAIR data infrastructure is critical to unlocking the vast potential of research data in reticular chemistry. By enabling data to be easily accessed and analyzed using advanced tools like data analytics and artificial intelligence, we can propel scientific exploration to new heights. With a “findable and AI-ready” approach to data management, we can anticipate a revolution in the way that science is conducted, driving greater efficiency and discovery in these critical fields. Exciting new developments are starting to emerge in this area, pointing the way toward a bright future for data management in material science and reticular chemistry. With growing interest and investment in FAIR data infrastructure, we are beginning to see promising signs of progress that could transform the way we approach research. From cutting-edge tools and techniques for data analysis to innovative approaches to collaboration and knowledge sharing, the future looks incredibly promising for those willing to embrace this new paradigm. Scheffler et al. [343] after presenting the current state of the art in the field, the challenges facing it were highlighted, and potential solutions offered by FAIRmat were proposed. Specifically, the existing landscape was described in detail, with a focus on the most significant issues and limitations. Following this, the obstacles that need to be overcome were emphasized, such as the lack of standardization and the difficulty in sharing data between different scientific communities. Finally, FAIRmat's envisioned solutions were presented, which aim to address these challenges by promoting open science practices, developing common data standards, and creating a network of collaborative research efforts. Let us exemplify with the technique chosen by Draxl’s research group to establish digital FAIR data-management workflows [343]. To lay the foundation for digital FAIR data-management workflows for material characterization, the authors focused on a set of key experimental techniques. Five such techniques have been selected for initial focus, with the aim of establishing effective workflows that can be scaled up in the future (Fig. 36). These techniques have been chosen based on their relevance to the specific material properties of interest, as well as their ability to generate high-quality data that can be easily managed and shared in a digital format. By prioritizing these techniques and establishing effective workflows for them, it will be possible to streamline the process of managing and sharing material characterization data, while also enabling future scalability and expansion as needed. Inspired by this study, we would shed light on the enormous potential of FAIR data in the field of RF-NPs, while also revealing the significant gap between our expectations and the current limitations of available tools. Given the scale of this opportunity, it's clear that RF-NPs researchers cannot afford to ignore the possibilities that a FAIR data approach offers. By embracing this cutting-edge methodology, we can unlock new insights, develop more powerful tools, and push the boundaries of what is possible in this exciting field. The stage is set for a thrilling new era of RF-NPs research, and those who are ready to seize the opportunity are poised to make groundbreaking discoveries that will shape the future of science.

*One can best anticipate what lies ahead by actively shaping it.*

## Summary and Future Directions

One of the most exciting areas of research in the field of reticular nanoscience is the ability to design and tailor nanosized reticular materials to suit a wide range of applications. This unprecedented level of design freedom offers tremendous potential for customization and innovation, making it a highly promising avenue for further exploration and discovery. However, while the field of reticular chemistry has made significant progress since its inception, it is still considered to be in a developing stage and has not yet reached a state of full maturity. There are many revelations of possible avenues that can enrich this chemistry repertoire by persistently seeking answers to challenging inquiries: Given the advancements and potential applications of POF chemistry, is there a gap between what's possible and what can be achieved with current methods? How can we balance the advancement of the theoretical foundations of reticular chemistry with a practical emphasis on the implementation and utility of the resulting RF-NPs? And what measures can we take to facilitate the involvement and meaningful participation of new and upcoming researchers in this rapidly developing field? Aiming at these challenges, there is no one-size-fits-all approach to answering these questions, only the potential of digital reticular chemistry is the best future direction to pursue. After a quarter-century of dedicated research, this review provides an overview of the notable progress made by RF-NPs specialists over this period. Their achievements encompass the discovery of new materials that exhibit unprecedented characteristics, the evolution of isoreticular chemistry, and the creation of sophisticated methods for the synthesis and analysis of RF-NPs. Moreover, the review delves into recent advancements in digital reticular chemistry and their influence on the field of materials science engineering.

Finally, RF-NPs have come a long way, but we're not done yet! To keep the ball rolling, we need to delve deeper into the world of chemical synthesis techniques, step up our observation game, make more data-driven discoveries, and deepen our understanding of the subject matter. By doing so, we can unlock even more potential in the field of RF-NPs and pave the way for new and exciting applications while betting on the boundless creativity and imagination of the chemist/material designer. We anticipate that within the next quarter-century, there will be a surge of interest in advancing and refining RF-NPs, using the latest and most cutting-edge technologies in reticular chemistry. This shift in focus is expected to bring about significant breakthroughs and innovations, ushering in a new era of possibilities in the field.
